# Concise and Stereospecific Total Synthesis of Arenastatin A and Its Segment B Analogs

**DOI:** 10.3390/molecules29174058

**Published:** 2024-08-27

**Authors:** Yurina Mihara, Haruki Kadoya, Soki Kakihana, Naoyuki Kotoku

**Affiliations:** College of Pharmaceutical Sciences, Ritsumeikan University, 1-1-1 Noji-Higashi, Kusatsu, Shiga 525-8577, Japan; ph0118hi@ed.ritsumei.ac.jp (Y.M.); k.haruki0507@icloud.com (H.K.); ph0158kx@ed.ritsumei.ac.jp (S.K.)

**Keywords:** arenastatin A, depsipeptide, total synthesis, late-stage diversification

## Abstract

A novel and concise synthetic method for arenastatin A, a cytotoxic cyclic depsipeptide of marine origin, was developed in this study. The convergent assembly of the four segments, including the cross-metathesis reaction, gave a cyclization precursor, and Fmoc deprotection caused simultaneous macrocyclization. The Corey–Chaykovsky reaction using a chiral sulfur ylide afforded arenastatin A with complete stereoselectivity in the longest linear sequence of seven reaction steps from the known compound. Using this synthetic method, some analogs of segment B were prepared through a late-stage diversification strategy. The simple S_N_2 reaction of the thiolate toward the tosylate precursor, prepared using almost the same synthetic method as described above, provided the desired sulfide analogs.

## 1. Introduction

**Figure 1:**
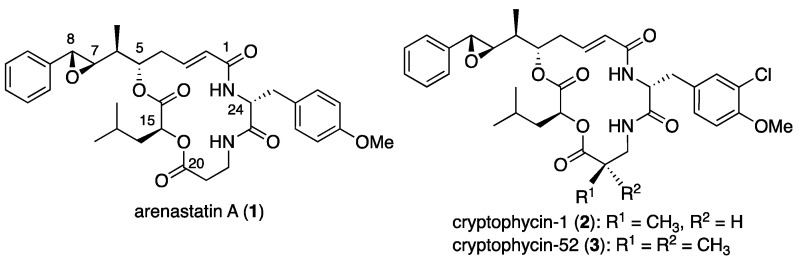
Chemical structures of arenastatin A (**1**), cryptophycin-1 (**2**), and -52 (**3**).

Marine natural products are rich and promising sources of drug candidates for anticancer drug discovery [1,2]. In 1994, arenastatin A (**1**, Figure 1), a cyclic depsipeptide with potent cytotoxicity (IC_50_ = 5 pg/mL against KB cells), was isolated from the Okinawan marine sponge *Dysidea arenaria* [3]. The chemical structure of the compound was determined by NMR analysis and total synthesis [4,5]. Cryptophycins, a family of closely related depsipeptides, are found in the terrestrial cyanobacterium *Nostoc* sp. Some of these compounds, such as cryptophycin 1 (**2**), exhibit potent cytotoxicity [6]. Since then, a number of total synthesis and medicinal chemistry studies have been conducted, resulting in the discovery of a potent analog, cryptophycin-52 [7,8,9]. Although the clinical phase trial of **3** was discontinued, the high potency of the compound has attracted attention in anticancer drug development as a payload for antibody–drug conjugates (ADC) or other conjugate molecules [10,11,12].

**Figure 2:**
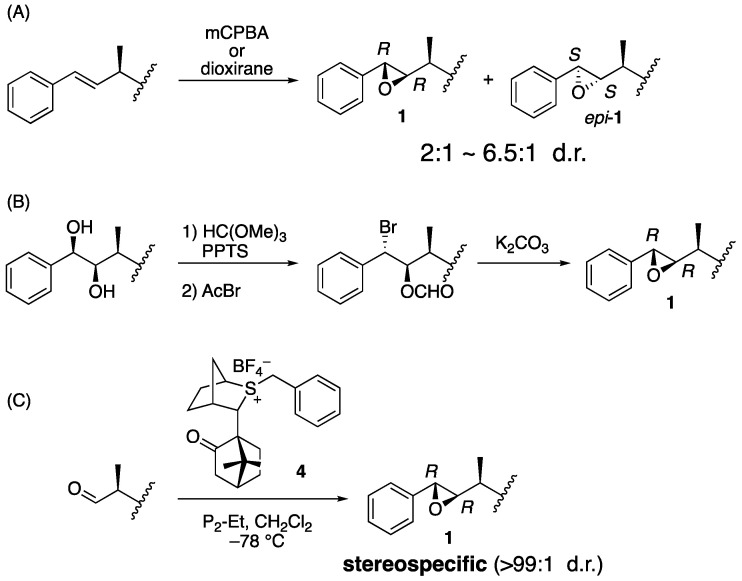
Reported synthetic method of 7,8-epoxide moiety of **1**. (**A**) Oxidation of the alkene precursor. (**B**) Cyclization of bromohydrin derivative. (**C**) Corey–Chaykovsky reaction using chiral sulfonium salt **4**.

Biological evaluation of arenastatin A (**1**) and related compounds or synthetic analogs revealed that the stereochemistry of the compound is important for its potent cytotoxicity. Therefore, stereoselective preparation of the respective asymmetric centers is essential. One of the most important parts is the 7,8-epoxide moiety because its (7*S*,8*S*) epimer has no cytotoxicity [13]. Two major approaches have been used in the reported total syntheses of **1**, **2**, and their analogs: In an earlier general approach, oxidation of the alkene precursor using *m*-chloroperbenzoic acid (mCPBA) or dioxirane in the final stage of synthesis was applied (Figure 2A) [5,14,15,16,17,18,19,20,21,22,23,24]. It gave a mixture of isomers, up to β:α = 6.5:1 by chiral dioxirane [24], and HPLC is needed for the purification. Another reliable approach is cyclization of the bromohydrin derivative, which was prepared from the corresponding 7,8-diol derivative, although relatively long reaction steps are required (Figure 2B) [25,26]. Previously, we developed a stereoselective synthesis of arenastatin A (**1**) (Figure 2C) [27]. The Corey–Chaykovsky reaction using chiral sulfonium salt **4**, developed by Aggarwal et al. [28], was effective in constructing the natural 7*R*,8*R*-epoxide moiety in an almost stereospecific manner (>99:1 d.r.). This avoids HPLC separation of the undesired diastereomer, enabling us to prepare a sufficient amount of **1** and analogs.

Based on the above background, a more efficient and scalable synthesis of **1** and its analogs would be interesting for anticancer drug discovery. In this study, we developed a short-step, stereospecific synthetic method for arenastatin A (**1**). Additionally, some analogs of segment B were prepared using a late-stage diversification approach. The details of this study, together with the bioactivity of the analogs, are described here.

## 2. Results and Discussions

### 2.1. Retrosynthetic Analysis of Arenastatin A

**Figure 3:**
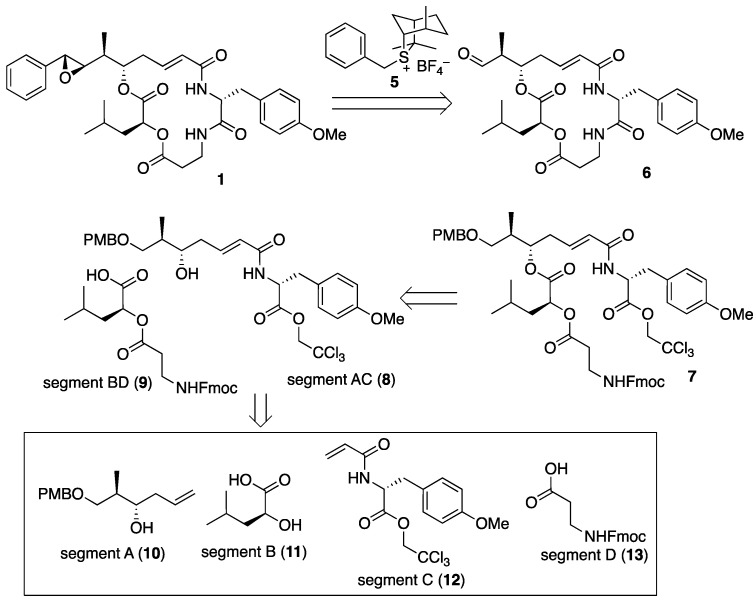
Retrosynthetic analysis of arenastatin A (**1**).

The retrosynthetic analysis of arenastatin A (**1**) is shown in Figure 3. The 7*R*,8*R*-epoxide moiety could be constructed by the Corey–Chaykovsky reaction in the final step, and aldehyde **6** could be prepared through the macrolactam formation of linear precursor **7**. Assembly of the four segments A–D (**10**–**13**) would give **7**. As the four segments were linearly and successively connected (A→AB→ABC→ABCD) in our previous synthetic method of **1** [27], the convergent approach by changing the order of assembly would reduce the linear reaction steps, leading to the increased overall yield.

A remained difficulty in the scalable synthesis of **1** and its analogs might be the synthesis of sulfonium salt **4**, because it requires the hetero Diels–Alder reaction between cyclopentadiene and thioaldehyde, generated by the photoirradiated degradation of phenacyl sulfide [29,30]. Therefore, we decided to use another chiral sulfonium salt, **5** in the reaction. The precursor sulfide isothiocineol can be prepared in only one step from limonene in a scalable manner, and similar stereoselectivities to those using sulfonium salt **5** have been reported [31].

### 2.2. Total Synthesis of Arenastatin A

**Scheme 1:**
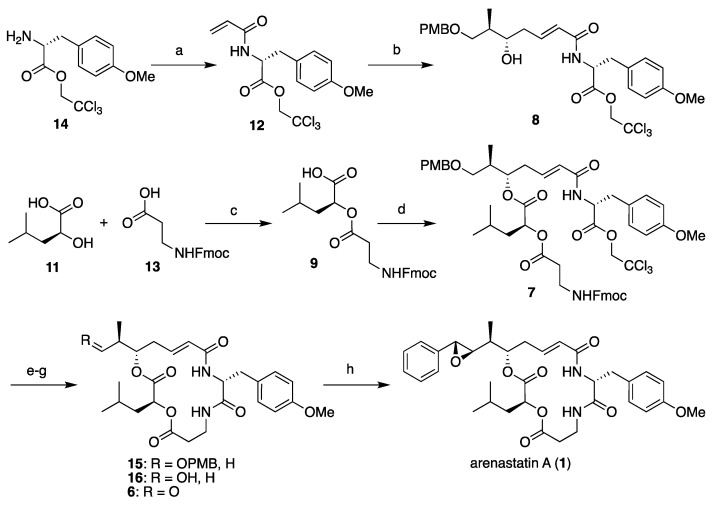
Total synthesis of arenastatin A (**1**). Reagents and conditions: (a) acryloyl chloride, NEt_3_, CH_2_Cl_2_, 0 °C, 77%; (b) **10**, Grubbs II catalyst, CH_2_Cl_2_, reflux, 95%; (c) SOCl_2_, DMAP, CH_2_Cl_2_, quant.; (d) 2,4,6-trichlorobenzoyl chloride, NEt_3_, DMAP, THF, 76%; (e) piperidine, DMF, 84%; (f) TFA, (CH_3_)_2_S, CH_2_Cl_2_, 87%; (g) Dess–Martin periodinane, CH_2_Cl_2_; (h) **5**, Phosphazene P_2_-Et, CH_2_Cl_2_, −78 °C, 81% (2 steps).

First, the four segments were assembled, as shown in Scheme 1. The 2,2,2-trichloroethyl ester of (*R*)-*O*-methyltyrosine (**14**) [14] was converted into the corresponding acrylamide **12** (segment C). Cross-metathesis reaction with segment A (**10**), separately prepared through Keck’s stereoselective allylation [32,33,34], yielded segment AC (**8**) in excellent yield using a Grubbs II catalyst (M-204, Umicore: Brussels, Belgium) [35]. A large excess of either segment is not required in this case. By contrast, commercially available Fmoc-β-alanine (**13**, segment D) was converted to the corresponding acyl chloride using thionyl chloride, and subsequent treatment with l-leucic acid (**11**, segment B) provided segment BD (**9**) in quantitative yield. Then, the two fragments were assembled using the Yamaguchi esterification protocol (2,4,6-trichlorobenzoly chloride, NEt_3_, DMAP) to give cyclization precursor **7**. According to a report by Norman et al. [24], the treatment of **7** with piperidine resulted in the removal of the Fmoc group and simultaneous macrocyclization, affording macrolactam **15** in good yield. By changing the order of segment assembly and the choice of coupling reaction, a shortened linear reaction sequence and a higher overall yield than those of our previous method [27] were realized. Finally, the cleavage of the *p*-methoxybenzyl ether with TFA and subsequent Dess–Martin oxidation gave the desired aldehyde (**6**), which was then subjected to the Corey–Chaykovsky reaction.

**Scheme 2:**
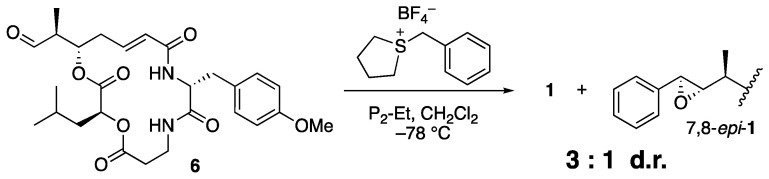
Attempted Corey–Chaykovsky reaction using achiral sulfonium salt.

As expected, the reaction with sulfonium salt **5** proceeded smoothly to afford arenastatin A (**1**) in good yield. Phosphazene P_2_-Et was found to be the best base, and nearly complete decomposition was observed using LHMDS. Under optimal conditions (1.4 equiv. of **5** and phosphazene P_2_-Et in CH_2_Cl_2_ (0.2 M) at −78 °C), 81% isolated yield and complete stereoselectivity were accomplished. None of the other diastereomers were detected by ^1^H NMR or HPLC analyses, and nearly pure products were easily obtained by simple silica gel chromatography on a scale of ~100 mg. The same reaction using an achiral sulfonium salt provided a substantial amount of 7,8-*epi*-**1** with a 3:1 diastereomeric ratio, clearly indicating that the chiral, rigid structure of the isothiocineole skeleton was essential for stereoselectivity (Scheme 2). 

### 2.3. Diverted Synthesis of Arenastatin A Analogs by Late-Stage Functionalization

Because natural products have complex chemical structures bearing various functional groups, the synthesis of natural product analogs is often difficult. This is mainly because a laborious, separate synthesis of the respective analogs from different starting materials/precursor segments might be required. The late-stage diversification strategy is a useful alternative approach in which only a simple derivatization reaction is conducted on a common late-stage precursor. It provides various analogs in a simple manner, enabling effective analysis of the structure–activity relationship (SAR) [36].

**Figure 4:**
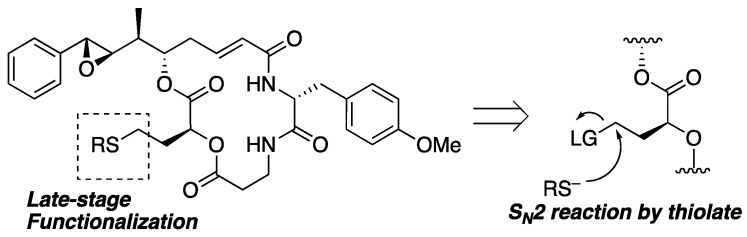
Synthetic strategy of late-stage functionalization.

Among the many approaches, we planned to apply a simple S_N_2 reaction to prepare analogs through late-stage functionalization, as shown in Figure 4. Nucleophilic attack of the thiolate toward the primary sp^3^-carbon with a leaving group at the terminal of segment B could give substitution products in the presence of labile functionalities such as epoxide, ester, and α,β-unsaturated carbonyl moieties. A similar approach for the derivatization of pleuromutilins has been reported, resulting in the discovery of more potent antibacterial analogs than natural products [37]. Because retained cytotoxicity was observed for the 15-epimer of **1** [23], further derivatization at this position could lead to novel analogs or bioconjugates.

**Scheme 3:**
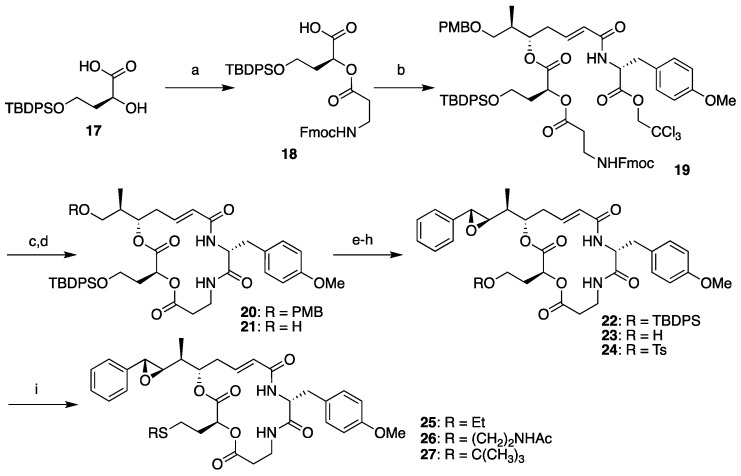
Diverted synthesis of arenastatin A analogs. Reagents and conditions: (a) **13**, SOCl_2_, DMAP, CH_2_Cl_2_, 69%; (b) **8**, 2,4,6-trichlorobenzoyl chloride, NEt_3_, DMAP, THF, 92%; (c) piperidine, DMF, 85%; (d) TFA, (CH_3_)_2_S, CH_2_Cl_2_, 83%; (e) Dess–Martin periodinane, CH_2_Cl_2_; (f) **5**, Phosphazene P_2_-Et, CH_2_Cl_2_, −78 °C, 64% (2 steps); (g) TBAF, CH_3_COOH, THF, 0 °C; (h) TsCl, pyridine, CH_2_Cl_2_, 44% (2 steps); (i) R-SH, NaH, THF or DMF, 0 °C, 49% for **25**; 72% for **26**; 0% for **27**.

The synthesis of segment B analogs was performed as shown in Scheme 3, starting from compound **17** as a surrogate for l-leucic acid. Compound **17** was prepared from l-malic acid according to the literature [38] and coupled with segment D (**13**) to yield **18**. Condensation between **18** and segment AC (**8**) proceeded smoothly, and subsequent Fmoc deprotection-macrocyclization by the treatment of compound **19** with piperidine provided macrolactam **20** in good yield. Subsequent cleavage of the PMB ether and Dess–Martin oxidation of the resulting primary hydroxyl group of **21** afforded the corresponding aldehyde, which was subjected to the Corey–Chaykovsky reaction. Complete stereoselectivity was observed, and (7*R*,8*R*)-**22** was obtained as the sole product. Clean removal of the TBDPS group was achieved by treating **22** with tetra-*n*-butylammonium fluoride (TBAF) in the presence of acetic acid, and subsequent tosylation provided the common derivatization precursor **24** in moderate yield. Objective late-stage functionalization worked effectively by the addition of a thiolate solution to **24**, affording the corresponding sulfide analogs **25** and **26**. The attempted synthesis of *tert*-butyl sulfide analog **27** failed, probably because of steric repulsion.

The cytotoxicity of analogs **25** and **26** together with arenastatin A (**1**) against human epidermoid carcinoma KB3-1 cells was evaluated, as summarized in Table 1. Ethyl sulfide analog **25** exhibited approximately one-tenth of the cytotoxicity of **1** (IC_50_ = 1.8 nM), and less activity was observed for the acetamidoethyl sulfide analog **26** (IC_50_ = 16.0 nM). These results imply that a longer side chain at this position would lead to diminished cytotoxicity, although more detailed SAR information is required. 

## 3. Materials and Methods

### 3.1. General

The following instruments were used to obtain physical data: a JASCO DIP-370 digital polarimeter (L = 50 mm) for specific rotations (JASCO: Tokyo, Japan); a JEOL JNM-ECZ500R/S1 (^1^H-NMR: 500 MHz, ^13^C-NMR: 125 MHz) spectrometer for ^1^H and ^13^C NMR data (JEOL: Tokyo, Japan) using tetramethylsilane as an internal standard; a Waters Xevo G2-XS Q-Tof mass spectrometer for ESI-Q-TOF MS (Waters: Milford, MA, USA). Silica gel (Kanto (Tokyo, Japan) 63–210 μm) and pre-coated thin layer chromatography (TLC) plates (Merck (Darmstadt, Germany) 60F_254_) were used for column chromatography and TLC. The spots on the TLC plates were detected by spraying with an acidic *p*-anisaldehyde solution (*p*-anisaldehyde: 25 mL, *c*-H_2_SO_4_: 25 mL, AcOH: 5 mL, EtOH: 425 mL) with subsequent heating. Unless otherwise noted, all reactions were performed under an N_2_ atmosphere. Hard copies of ^1^H and ^13^C NMR, and HRMS spectra can be found as Supplementary Materials.

### 3.2. Antiproliferative Activity of the Compounds against Cancer Cells

KB3-1 cells were cultured in RPMI-1640 medium supplemented with 10% heat-inactivated fetal bovine serum and kanamycin (50 μg/mL). Cells were plated into 96-well microplates at 2 × 10^4^ cells/100 μL assay medium/well, and various concentrations of test compounds were added to each well as a 10% DMSO/EtOH solution (1 µL). The plates were incubated at 37 °C in a humidified atmosphere of 5% CO_2_ for 72 h, and cell proliferation was determined by MTT colorimetric assay, and the averaged IC_50_ values of the triplicate experiment were shown in Table 1.

### 3.3. Synthesis

#### 3.3.1. 2,2,2-Trichloroethyl (*R*)-2-Acrylamido-3-(4-methoxyphenyl)propanoate (**12**)

NEt_3_ (0.42 mL, 3.1 mmol) and acryloyl chloride (0.25 mL, 3.1 mmol) were added to a solution of **14** (0.77 g, 2.3 mmol) in CH_2_Cl_2_ (12 mL) under N_2_ atmosphere at 0 °C, and the whole mixture was stirred for 4 h. Then, 1 M HCl aq. was added to the reaction solution and extracted with ethyl acetate. The organic layer was dried over Na_2_SO_4_ and was concentrated in vacuo. The crude material was purified by column chromatography (SiO_2_; *n*-hexane/AcOEt = 3:1) to afford **12** (0.69 g, 77%) as a white-yellow solid.

mp: 78.0–79.2 °C. ${\left[\alpha \right]}_{D}^{23}$ = −54.0° (*c* = 1.54 in CHCl_3_). ^1^H NMR (500 MHz, CDCl_3_) δ: 7.07 (d, *J* = 8.5 Hz, 2H), 6.83 (d, *J* = 8.6 Hz, 2H), 6.30 (dd, *J* = 17.0, 1.3 Hz, 1H), 6.09 (dd, *J* = 17.0, 10.4 Hz, 1H), 5.91 (d, *J* = 7.8 Hz, 1H), 5.69 (dd, *J* = 10.3, 1.3 Hz, 1H), 5.10–5.06 (m, 1H), 4.78 (d, *J* = 11.9 Hz, 1H), 4.74 (d, *J* = 11.9 Hz, 1H), 3.78 (s, 3H), 3.23 (dd, *J* = 14.2, 5.8 Hz, 1H), 3.17 (dd, *J* = 14.2, 5.9 Hz, 1H). ^13^C NMR (125 MHz, CDCl_3_) δ: 170.3, 165.2, 159.0, 130.4 (2C), 130.1, 127.8, 127.2, 114.3 (2C), 94.3, 74.8, 55.3, 53.2, 36.7. HR-ESI MS: *m*/*z* 402.0043, calcd for C_15_H_16_Cl_3_NO_4_Na. Found: 402.0032.

#### 3.3.2. 2,2,2-Trichloroethyl (*R*)-2-((5*S*,6*R*,*E*)-5-Hydroxy-7-((4-methoxybenzyl)oxy)-6-methylhept-2-enamido)-3-(4-methoxyphenyl)propanoate (**8**)

Grubbs II catalyst (M204, Umicore: 36 mg, 42 μmol) was added to a solution of **10** (0.39 g, 1.0 mmol) and **12** (0.21 g, 0.85 mmol) in CH_2_Cl_2_ (10 mL) under N_2_ atmosphere at rt, and the reaction solution was heated under reflux at 50 °C for 17 h. The reaction solution was then cooled to rt, and the solvent was removed under reduced pressure below 30 °C. The crude material was purified by column chromatography (SiO_2_; *n*-hexane/AcOEt = 2:1) to afford **8** (0.49 g, 95%) as light-brown waxy oil.

${\left[\alpha \right]}_{D}^{23}$ = −68.1° (*c* = 0.90 in CHCl_3_). ^1^H NMR (500 MHz, CDCl_3_) δ: 7.23 (d, *J* = 8.7 Hz, 2H), 7.07 (d, *J* = 8.3 Hz, 2H), 6.94–6.89 (m, 1H), 6.87 (d, *J* = 8.8 Hz, 2H), 6.83 (d, *J* = 8.7 Hz, 2H), 5.88–5.84 (m, 2H), 5.08–5.04 (m, 1H), 4.76 (d, *J* = 11.9 Hz, 1H), 4.72 (d, *J* = 11.9 Hz, 1H), 4.44 (s, 2H), 3.80 (s, 3H), 3.77 (s, 3H), 3.67–3.62 (m, 1H), 3.58 (dd, *J* = 9.4, 4.0 Hz, 1H), 3.42 (dd, *J* = 9.3, 7.8 Hz, 1H), 3.20 (dd, *J* =14.2, 5.9 Hz, 1H), 3.13 (dd, *J* =14.2, 5.9 Hz, 1H), 2.44–2.39 (m, 1H), 2.33–2.27 (m, 1H), 1.89–1.83 (m, 1H), 0.88 (d, *J* = 6.9 Hz, 3H). ^13^C NMR (125 MHz, CDCl_3_) δ: 170.4, 165.4, 159.4, 158.9, 142.5, 130.4 (2C), 129.7, 129.5 (2C), 127.3, 125.1, 114.3 (2C), 114.0 (2C), 94.4, 75.2, 74.8, 74.7, 73.2, 55.4, 55.3, 53.2, 37.8, 37.6, 36.9, 13.9. HR-ESI MS: *m*/*z* 602.1479, calcd for C_28_H_35_Cl_3_NO_7_. Found: 602.1461.

#### 3.3.3. (*S*)-2-((3-((((9*H*-Fluoren-9-yl)methoxy)carbonyl)amino)propanoyl)oxy)-4-methylpentanoic acid (**9**)

SOCl_2_ (54.3 μL, 0.76 mmol) was added to a solution of *N*-Fmoc-β-alanine (**13**, 0.24 g, 0.76 mmol) in CH_2_Cl_2_ (0.7 mL) under N_2_ atmosphere at rt. After the reaction solution was sonicated for 30 min at rt, DMAP (92 mg, 0.76 mmol) was added and sonicated for another 15 min. Then, this reaction solution was slowly added, over 2 min, to a cocktail containing L-leucic acid (**11**, 50 mg, 0.38 mmol) and DMAP (92 mg, 0.76 mmol) in CH_2_Cl_2_ (1.8 mL) at 0 °C. The mixture was stirred at rt for 2 h, then sat. NaHCO_3_ aq. and *n*-hexane were added to stop the reaction, and the aqueous layer was collected. This aqueous layer was acidified with 1 M HCl aq. and extracted with CH_2_Cl_2_. The organic layer was dried over Na_2_SO_4_ and was concentrated in vacuo. The crude material was purified by column chromatography (SiO_2_; *n*-hexane/AcOEt/AcOH = 60:10:1) to afford **9** (0.17 g, quant.) as colorless oil.

${\left[\alpha \right]}_{D}^{23}$ = −9.2° (*c* = 1.11 in CHCl_3_). ^1^H NMR (500 MHz, DMSO-*d*_6_) δ: 7.89 (d, *J* = 7.6 Hz, 2H), 7.67 (d, *J* = 7.6 Hz, 2H), 7.41 (t, *J* = 7.4 Hz, 2H), 7.37 (d, *J* = 5.5 Hz, 1H), 7.32 (t, *J* = 7.4 Hz, 2H), 4.83 (dd, *J =* 8.3, 3.1 Hz, 1H), 4.29 (d, *J* = 6.9 Hz, 2H), 4.20 (t, *J* = 6.9 Hz, 1H), 3.24 (q, *J* = 6.9 Hz, 2H), 2.52 (t, *J* = 7.0 Hz, 2H), 1.71–1.66 (m, 2H), 1.59–1.54 (m, 1H), 0.89 (d, *J* = 6.5 Hz, 3H), 0.86 (d, *J* = 6.5 Hz, 3H). ^13^C NMR (125 MHz, CDCl_3_) δ: 175.6, 172.1, 156.6, 144.03, 143.99, 141.4, 127.8 (2C), 127.2 (2C), 125.2 (2C), 120.1 (3C), 70.9, 67.1, 47.2, 39.5, 36.8, 34.6, 24.7, 23.1, 21.5. HR-ESI MS: *m*/*z* 424.1760, calcd for C_24_H_26_NO_6_. Found: 424.1742.

#### 3.3.4. (2*R*,3*S*,*E*)-1-((4-Methoxybenzyl)oxy)-7-(((*R*)-3-(4-methoxyphenyl)-1-oxo-1-(2,2,2-trichloroethoxy)propan-2-yl)amino)-2-methyl-7-oxohept-5-en-3-yl (*S*)-2-((3-((((9H-fluoren-9-yl)methoxy)carbonyl)amino)propanoyl)oxy)-4-methylpentanoate (**7**)

2,4,6-Trichlorobenzoyl chloride (70 μL, 0.43 mmol) and DMAP (6.6 mg, 0.054 mmol) were sequentially added to a solution of **8** (130 mg, 0.22 mmol), **9** (101 mg, 0.24 mmol), and NEt_3_ (60 μL, 0.43 mmol) in THF (1.1 mL) under N_2_ atmosphere at 0 °C, and the reaction solution was brought to rt and stirred overnight. Then, sat. citric acid aq. was added to the reaction solution and extracted with CHCl_3_. The organic layer was then washed with sat. NaHCO_3_ aq. The organic layer was dried over Na_2_SO_4_ and was concentrated in vacuo. The crude material was purified by column chromatography (SiO_2_; *n*-hexane/AcOEt = 4:1) to afford **7** (167 mg, 76%) as light brown oil.

${\left[\alpha \right]}_{D}^{23}$ = −13.5° (*c* = 1.71 in CHCl_3_). ^1^H NMR (500 MHz, CDCl_3_) δ: 7.75 (d, *J* = 7.5 Hz, 2H), 7.61 (d, *J* = 7.2 Hz, 2H), 7.38 (t, *J* = 7.4 Hz, 2H), 7.28 (t, *J* = 7.5 Hz, 2H), 7.20 (d, *J* = 8.5 Hz, 2H), 7.05 (d, *J* = 8.6 Hz, 2H), 6.84 (d, *J* = 8.7 Hz, 2H), 6.82–6.79 (m, 1H), 6.76 (d, *J* = 8.7 Hz, 2H), 6.38 (d, *J* = 8.0 Hz, 1H), 5.87–5.83 (m, 2H), 5.16–5.12 (m, 1H), 5.09–5.05 (m, 1H), 4.94 (dd, *J* = 9.7, 3.9 Hz, 1H), 4.73 (d, *J* = 11.9 Hz, 1H), 4.69 (d, *J* = 11.9 Hz, 1H), 4.41–4.31 (m, 2H), 4.38 (d, *J* = 8.4 Hz, 2H), 4.22 (t, *J* = 7.3 Hz, 1H), 3.77 (s, 3H), 3.74 (s, 3H), 3.54–3.41 (m, 2H), 3.35 (dd, *J* = 9.4, 5.9 Hz, 1H), 3.27 (dd, *J* = 9.4, 5.8 Hz, 1H), 3.17 (dd, *J* = 14.2, 6.0 Hz, 1H), 3.10 (dd, *J* = 14.2, 6.3 Hz, 1H), 2.68–2.63 (m, 1H), 2.58–2.52 (m, 1H), 2.51–2.43 (m, 2H), 2.13–2.08 (m, 1H), 1.78–1.71 (m, 2H), 1.57–1.52 (m, 1H), 0.95–0.92 (m, 9H). ^13^C NMR (125 MHz, CDCl_3_) δ: 172.3, 170.5, 165.4, 159.2, 158.8, 156.6, 144.1 (2C), 141.4 (2C), 139.8, 130.5 (2C), 130.3, 129.3 (2C), 127.8 (2C), 127.6, 127.1 (2C), 125.7 (2C), 125.4, 125.3, 120.0 (2C), 114.1 (2C), 113.9 (2C), 94.4, 75.3, 74.7, 72.8, 71.7, 71.2, 66.9, 55.3, 55.3, 53.3, 47.3, 39.5, 37.0, 36.8, 36.7, 34.7, 32.9, 24.8, 23.1, 21.6, 13.3. HR-ESI MS: *m*/*z* 1031.3031, calcd for C_52_H_59_Cl_3_N_2_O_12_Na. Found: 1031.3069.

#### 3.3.5. (3*S*,10*R*,16*S*,*E*)-3-Isobutyl-10-(4-methoxybenzyl)-16-((*R*)-1-((4-methoxybenzyl)oxy)propan-2-yl)-1,4-dioxa-8,11-diazacyclohexadec-13-ene-2,5,9,12-tetraone (**15**)

Piperidine (34.1 μL) was added to a solution of **7** (70 mg, 68.9 μmol) in DMF (2.3 mL), at rt, and stirred overnight. Then, H_2_O was added to the reaction solution and extracted with ethyl acetate. The organic layer was dried over Na_2_SO_4_ and was concentrated in vacuo. The crude material was purified by column chromatography (SiO_2_; *n*-hexane/AcOEt = 2:1) to afford **15** (37 mg, 84%) as a colorless amorphous solid.

${\left[\alpha \right]}_{D}^{23}$ = +49.2° (*c* = 1.12 in CHCl_3_). ^1^H NMR (500 MHz, CDCl_3_) δ: 7.21 (d, *J* = 8.6 Hz, 2H), 7.12 (d, *J* = 8.6 Hz, 2H), 7.04 (t, *J* = 5.7 Hz, 1H), 6.86 (d, *J* = 9.1 Hz, 2H), 6.80 (d, *J* = 8.7 Hz, 2H), 6.72–6.66 (m, 1H), 5.81–5.78 (m, 1H), 5.71 (dd, *J* = 15.1, 1.6 Hz, 1H), 5.19–5.15 (m, 1H), 4.91 (dd, *J* = 9.4, 4.0 Hz, 1H), 4.76–4.71 (m, 1H), 4.41 (d, *J* = 11.7 Hz, 1H), 4.37 (d, *J* = 11.7 Hz, 1H), 3.79 (s, 3H), 3.77 (s, 3H), 3.55–3.49 (m, 1H), 3.48–3.41 (m, 1H), 3.36 (dd, *J* = 9.4, 6.0 Hz, 1H), 3.28 (dd, *J* = 9.4, 6.0 Hz, 1H), 3.15 (dd, *J* = 14.5, 5.7 Hz, 1H), 3.02 (dd, *J* = 14.5, 7.7 Hz, 1H), 2.56 (dd, *J* =6.9, 4.7 Hz, 1H), 2.45–2.32 (m, 2H), 2.07–1.98 (m, 1H), 1.72–1.64(m, 2H), 1.42–1.36 (m, 1H), 0.97 (d, *J* = 6.9 Hz, 3H), 0.90 (d, *J* = 6.2 Hz, 3H), 0.86 (d, *J* = 6.3 Hz, 3H). ^13^C NMR (125 MHz, CDCl_3_) δ: 172.9, 170.9, 170.6, 165.7, 159.3, 158.6, 142.2, 130.3 (2C), 130.2, 129.3 (2C), 128.7, 124.9, 114.2 (2C), 113.9 (2C), 75.6, 72.9, 71.4, 71.2, 55.4, 55.3, 54.3, 39.8, 37.9, 35.4, 35.3, 34.3, 32.6, 24.6, 22.9, 21.6, 13.7. HR-ESI MS: *m/z* 639.3282, calcd for C_35_H_47_N_2_O_9_. Found: 639.3289.

#### 3.3.6. (3*S*,10*R*,16*S*,*E*)-16-((*R*)-1-Hydroxypropan-2-yl)-3-isobutyl-10-(4-methoxybenzyl)-1,4-dioxa-8,11-diazacyclohexadec-13-ene-2,5,9,12-tetraone (**16**)

Trifluoroacetic acid (1.5 mL, 10%) and dimethyl sulfide (21.3 mg, 0.343 mmol) were added to a solution of **15** (73 mg, 0.114 mmol) in CH_2_Cl_2_ (22.8 mL) at rt, and the whole mixture was stirred for 15 min. Then, saturated NaHCO_3_ solution was slowly added to the reaction solution at 0 °C and extracted with CHCl_3_. The organic layer was dried over Na_2_SO_4_ and was concentrated in vacuo. The crude material was purified by column chromatography (SiO_2_; CHCl_3_/MeOH = 40:1) to afford **16** (51.3 mg, 87%) as white powder.

mp: 98.2–100.5 °C. ^1^H NMR (500 MHz, CDCl_3_) δ: 7.11 (d, *J* = 8.8 Hz, 2H), 7.02 (t, *J* = 5.8 Hz, 1H), 6.80 (d, *J* = 8.7 Hz, 2H), 6.73–6.66 (m, 1H), 5.79 (d, *J* = 8.2 Hz, 1H), 5.74 (d, *J* = 15.2 Hz, 1H), 5.16–5.12 (m, 1H), 4.94 (dd, *J* = 10.0, 4.1 Hz, 1H), 4.73–4.69 (m, 1H), 3.77 (s, 3H), 3.60 (dd, *J* = 10.9, 5.5 Hz, 1H), 3.54 (dd, *J* = 11.0, 5.5 Hz, 1H), 3.51–3.42 (m, 2H), 3.15 (dd, *J* = 14.4, 5.8 Hz, 1H), 3.01 (dd, *J* = 14.4, 7.7 Hz, 1H), 2.55 (dd, *J* = 6.8, 4.7 Hz, 2H), 2.53–2.49 (m, 1H), 2.43–2.36 (m, 1H), 1.94–1.89 (m, 1H), 1.76–1.65 (m, 2H), 1.47–1.42 (m, 1H), 0.99 (d, *J* = 7.1 Hz, 3H), 0.92 (d, *J* = 6.6 Hz, 3H), 0.88 (d, *J* = 6.6 Hz, 3H). ^13^C NMR (125 MHz, CDCl_3_) δ: 172.8, 170.9, 170.8, 165.7, 158.6, 142.0, 130.3 (2C), 128.7, 125.1, 114.2 (2C), 75.7, 71.4, 64.2, 55.3, 54.4, 39.8 (2C), 35.7, 35.3, 34.3, 32.6, 24.6, 23.0, 21.6, 13.5. HR-ESI MS: *m*/*z* 519.2706, calcd for C_27_H_39_N_2_O_8_. Found: 519.2705.

#### 3.3.7. Arenastatin A (**1**)

Dess–Martin periodinane (57.2 mg, 0.13 mmol) was added to a solution of **8** (46.6 mg, 0.090 mmol) in CH_2_Cl_2_ (0.57 mL) at rt and stirred for 3 h. Then, sat. Na_2_S_2_O_3_ aq. and sat. NaHCO_3_ aq. were added at 0 °C to stop the reaction and extracted with AcOEt. After drying the organic layer with Na_2_SO_4_, the solvent was removed under reduced pressure to obtain the crude aldehyde **6** (46.2 mg, quant.). To a solution of **6** (46.2 mg, 0.089 mmol) and sulfonium salt **5** (50.7 mg, 0.13 mmol) in anhydrous CH_2_Cl_2_ (0.4 mL) under N_2_ atmosphere was slowly added phosphazene P_2_-Et (41.9 mg, 0.13 mmol) in CH_2_Cl_2_ (40 μL) at −78 °C, and the whole mixture was stirred at −78 °C for 3 h. After stirring, brine was added to stop the reaction and extracted with CHCl_3_. The organic layer was dried over Na_2_SO_4_ and was concentrated in vacuo. The crude material was purified by column chromatography (SiO_2_; CHCl_3_/MeOH = 90:1) to afford **1** (44.0 mg, 81%) as white powder.

mp: 263.1–263.8 °C. ^1^H NMR (500 MHz, CDCl_3_) δ: 7.37–7.32 (m, 3H), 7.23–7.21 (m, 2H), 7.09 (d, *J* = 8.8 Hz, 2H), 6.98 (t, *J* = 6.9 Hz, 1H), 6.79 (d, *J* = 8.8 Hz, 2H), 6.71–6.65 (m, 1H), 5.67 (dd, *J* = 15.1, 1.7 Hz, 1H), 5.53 (d, *J* = 7.3 Hz, 1H), 5.21–5.17 (m, 1H), 4.86 (dd, *J* = 10.1, 3.6 Hz, 1H), 4.73–4.69 (m, 1H), 3.76 (s, 3H), 3.66 (d, *J* = 2.0 Hz, 1H), 3.56–3.50 (m, 1H), 3.40–3.34 (m, 1H), 3.12 (dd, *J* = 14.4, 5.7 Hz, 1H), 3.01 (dd, *J* = 14.5, 7.5 Hz, 1H), 2.90 (dd, *J* = 7.7, 2.1 Hz, 1H), 2.56–2.50 (m, 3H), 2.45–2.38 (m, 1H), 1.79–1.75 (m, 1H), 1.69–1.63 (m, 2H), 1.28–1.25 (m, 1H), 1.13 (d, *J* = 7.0 Hz, 3H), 0.82 (d, *J* = 6.3 Hz, 3H), 0.80 (d, *J* = 6.3 Hz, 3H). ^13^C NMR (125 MHz, CDCl_3_) δ: 173.0, 170.8 (2C), 165.5, 158.7, 141.3, 136.8, 130.3 (2C), 128.9 (2C), 128.7, 128.5, 125.8 (2C), 125.3, 114.2 (2C), 75.9, 71.3, 63.2, 59.2, 55.4, 54.3, 40.8, 39.6, 36.8, 35.3, 34.2, 32.6, 24.5, 22.9, 21.3, 13.7. HR-ESI MS: *m/z* 607.3019, calcd for C_34_H_43_N_2_O_8_. Found: 607.3045.

#### 3.3.8. (*S*)-2-((3-((((9*H*-Fluoren-9-yl)methoxy)carbonyl)amino)propanoyl)oxy)-4-((*tert*-butyldiphenylsilyl)oxy)butanoic acid (**18**)

SOCl_2_ (0.29 mL, 4.0 mmol) was added to a solution of **13** (1.25 g, 4.0 mmol) in CH_2_Cl_2_ (6 mL) under N_2_ atmosphere at rt. After the reaction solution was sonicated for 1 h, DMAP (0.55 g, 4.5 mmol) was added and sonicated for another 15 min. Then, this reaction solution was slowly added to a cocktail containing (*S*)-4-((*tert*-butyldiphenylsilyl)oxy)-2-hydroxybutanoic acid (**17**) (0.75 g, 2.1 mmol) and DMAP (0.55 g, 4.5 mmol) in CH_2_Cl_2_ (16.3 mL) over 2 min at 0 °C. The mixture was stirred at rt for 4 h, then sat. NaHCO_3_ aq. and *n*-hexane were added to stop the reaction, and the aqueous layer was collected. This aqueous layer was acidified with 1 M HCl aq. and extracted with CHCl_3_. The organic layer was dried over Na_2_SO_4_ and was concentrated in vacuo. The crude material was purified by column chromatography (SiO_2_; *n*-hexane/AcOEt/AcOH = 60:10:1) to afford **18** (0.95 g, 69%) as colorless oil.

${\left[\alpha \right]}_{D}^{23}$ = −5.2° (*c* = 1.06 in CHCl_3_). ^1^H NMR (500 MHz, CDCl_3_) δ: 10.5–9.0 (br, 1H), 7.72 (d, *J* = 7.6 Hz, 2H), 7.65–7.61 (m, 4H), 7.55 (d, *J* = 7.6 Hz, 2H), 7.42–7.39 (m, 2H), 7.38–7.34 (m, 6H), 7.29–7.25 (m, 2H), 5.52 (t, *J* = 6.4 Hz, 1H), 5.37 (dd, *J* = 8.9, 4.0 Hz, 1H), 4.32 (d, *J* = 7.9 Hz, 2H), 4.17 (t, *J* = 7.3 Hz, 1H), 3.80–3.76 (m, 1H), 3.71–3.66 (m, 1H), 3.49–3.44 (m, 2H), 2.53–2.50 (m, 2H), 2.19–2.15 (m, 1H), 2.05–2.00 (m, 1H), 1.02 (s, 9H). ^13^C NMR (125 MHz, CDCl_3_) δ: 175.5, 171.8, 156.6, 144.0, 144.0, 141.3, 135.7 (5C), 133.3, 133.2, 129.9 (2C), 127.9 (4C), 127.8 (2C), 127.2 (2C), 125.3 (2C), 120.1 (2C), 69.0, 67.1, 59.0, 47.2, 36.7, 34.5, 33.6, 26.9 (3C), 19.2. HR-ESI MS: *m/z* 650.2574, calcd for C_38_H_40_NO_7_Si. Found: 650.2552.

#### 3.3.9. (2*R*,3*S*,*E*)-1-((4-Methoxybenzyl)oxy)-7-(((*R*)-3-(4-methoxyphenyl)-1-oxo-1-(2,2,2-trichloroethoxy)propan-2-yl)amino)-2-methyl-7-oxohept-5-en-3-yl (*S*)-2-((3-((((9*H*-fluoren-9-yl)methoxy)carbonyl)amino)propanoyl)oxy)-4-((*tert*-butyldiphenylsilyl)oxy)butanoate (**19**)

2,4,6-Trichlorobenzoyl chloride (0.41 mL, 2.6 mmol) and DMAP (40 mg, 0.33 mmol) were sequentially added to a solution of **8** (0.53 g, 0.88 mmol), **18** (0.97 g, 1.5 mmol), and NEt_3_ (0.37 mL, 2.6 mmol) in THF (2.9 mL) under N_2_ atmosphere at 0 °C, and the reaction solution was brought to rt and stirred overnight. Then, sat. citric acid aq. was added to the reaction solution and extracted with CHCl_3_. The organic layer was then washed with sat. NaHCO_3_ aq. and dried with Na_2_SO_4_. Removal of the solvent from the organic layer under reduced pressure gave the crude product, which was purified by column chromatography (SiO_2_; *n*-hexane/AcOEt = 2:1) to afford **19** (0.99 g, 92%) as colorless oil.

${\left[\alpha \right]}_{D}^{23}$ = −11.4° (*c* = 1.70 in CHCl_3_). ^1^H NMR (500 MHz, CDCl_3_) δ: 7.73 (d, *J* = 7.0 Hz, 2H), 7.64–7.60 (m, 4H), 7.60–7.54 (m, 2H), 7.42–7.39 (m, 2H), 7.38–7.34 (m, 7H), 7.28–7.25 (m, 2H), 7.18 (d, *J* = 8.8 Hz, 2H), 7.02 (d, *J* = 8.7 Hz, 2H), 6.81 (d, *J* = 8.7 Hz, 2H), 6.79–6.76 (m, 1H), 6.74 (d, *J* = 8.6 Hz, 2H), 6.35 (d, *J* = 8.0 Hz, 1H), 5.85–5.81 (m, 2H), 5.18 (dd, *J* = 10.0, 3.5 Hz, 1H), 5.13–5.11 (m, 1H), 5.08–5.03 (m, 1H), 4.71 (d, *J* = 11.9 Hz, 1H), 4.65 (d, *J* = 11.9 Hz, 1H), 4.37 (d, *J* = 11.6 Hz, 1H), 4.33 (d, *J* = 11.6 Hz, 1H), 4.28 (t, *J* = 7.3 Hz, 1H), 4.20 (t, *J* = 7.5 Hz, 1H), 3.76–3.72 (m, 2H), 3.73 (s, 3H), 3.69 (s, 3H), 3.48–3.38 (m, 2H), 3.34 (dd, *J* = 9.4, 5.9 Hz, 1H), 3.25 (dd, *J* = 9.5, 5.9 Hz, 1H), 3.14 (dd, *J* = 14.2, 6.2 Hz, 1H), 3.08 (dd, *J* = 14.1, 6.2 Hz, 1H), 2.59–2.55 (m, 1H), 2.49–2.44 (m, 3H), 2.10–2.04 (m, 2H), 1.92–1.86 (m, 1H), 1.04 (s, 9H), 0.92 (d, *J* = 6.9 Hz, 3H). ^13^C NMR (125 MHz, CDCl_3_) δ: 172.2, 170.6, 170.3, 165.4, 159.2, 158.8, 156.6, 144.1, 141.4, 139.8, 135.6 (4C), 133.4, 130.5 (2C), 130.3, 129.9 (2C), 129.3 (3C), 127.9 (4C), 127.7 (2C), 127.5, 127.1 (2C), 125.7, 125.4, 125.3, 120.0 (2C), 114.1 (2C), 113.9 (3C), 94.4, 75.4, 74.7, 72.8, 71.2, 69.8, 66.9, 59.2, 55.3 (2C), 55.2 (2C), 53.3, 47.3, 37.0, 36.8, 34.5, 33.8, 33.0, 26.9 (4C), 19.3, 13.3. HR-ESI MS: *m/z* 1235.4026, calcd for C_66_H_74_Cl_3_N_2_O_13_Si. Found: 1235.4009.

#### 3.3.10. (3*S*,10*R*,16*S*,*E*)-3-(2-((*tert*-Butyldiphenylsilyl)oxy)ethyl)-10-(4-methoxybenzyl)-16-((*R*)-1-((4-methoxybenzyl)oxy)propan-2-yl)-1,4-dioxa-8,11-diazacyclohexadec-13-ene-2,5,9,12-tetraone (**20**)

Piperidine (40 μL, 0.41 mmol) was added to a solution of **19** (0.10 g, 81.7 μmol) in DMF (2.7 mL) at rt, and the whole mixture was stirred overnight. Then, H_2_O was added to the reaction solution and extracted with AcOEt. The organic layer was dried over Na_2_SO_4_ and was concentrated in vacuo. The crude material was purified by column chromatography (SiO_2_; *n*-hexane/AcOEt = 1:2) to afford **20** (59.7 mg, 85%) as colorless amorphous solid.

${\left[\alpha \right]}_{D}^{23}$ = +37.3° (*c* = 1.77 in CHCl_3_). ^1^H NMR (500 MHz, CDCl_3_) δ: 7.61–7.57 (m, 4H), 7.43–7.39 (m, 2H), 7.37–7.33 (m, 4H), 7.20 (d, *J* = 8.8 Hz, 2H), 7.12 (d, *J* = 8.8 Hz, 2H), 6.99 (t, *J* = 6.0 Hz, 1H), 6.85 (d, *J* = 8.8 Hz, 2H), 6.82 (d, *J* = 8.8 Hz, 2H), 6.72–6.66 (m, 1H), 5.71 (d, *J* = 15.2 Hz, 1H), 5.63 (d, *J* = 8.3 Hz, 1H), 5.15–5.11 (m, 1H), 5.09 (dd, *J* = 9.9, 3.7 Hz, 1H), 4.79–4.74 (m, 1H), 4.38 (d, *J* = 11.6 Hz, 1H), 4.35 (d, *J* = 11.6 Hz, 1H), 3.77 (s, 3H), 3.76 (s, 3H), 3.70–3.66 (m, 2H), 3.52–3.47 (m, 1H), 3.43–3.37 (m, 1H), 3.36 (dd, *J* = 9.3, 5.9 Hz, 1H), 3.30 (dd, *J* = 9.3, 5.6 Hz, 1H), 3.11 (dd, *J* = 14.4, 5.6 Hz, 1H), 3.04 (dd, *J* = 14.6, 7.5 Hz, 1H), 2.45–2.32 (m, 4H), 2.04–1.99 (m, 1H), 1.88–1.79 (m, 2H), 1.03 (s, 9H), 0.96 (d, *J* = 7.0 Hz, 3H). ^13^C NMR (125 MHz, CDCl_3_) δ: 172.8, 170.9, 170.6, 165.7, 159.3, 158.6, 142.2, 135.7 (2C), 135.6 (2C), 133.4 (3C), 130.5, 130.2 (2C), 130.0 (2C), 129.4, 129.2, 128.7, 127.9 (2C), 124.8, 114.2, 114.1, 114.0 (2C), 113.8, 75.9, 73.0, 71.3, 69.8, 69.6, 59.0, 55.3, 54.1, 37.9, 35.5, 34.4, 34.0, 32.5, 26.9 (3C), 19.3 (2C), 13.8. HR-ESI MS: *m/z* 865.4095, calcd for C_49_H_61_N_2_O_10_Si. Found: 865.4089.

#### 3.3.11. (3*S*,10*R*,16*S*,*E*)-3-(2-((*tert*-Butyldiphenylsilyl)oxy)ethyl)-16-((*R*)-1-hydroxypropan-2-yl)-10-(4-methoxybenzyl)-1,4-dioxa-8,11-diazacyclohexadec-13-ene-2,5,9,12-tetraone (**21**)

Trifluoroacetic acid (1.1 mL, 10%) and dimethyl sulfide (16 mg, 0.25 mmol) were added to a solution of **20** (73 mg, 84.5 μmol) in CH_2_Cl_2_ (16.9 mL), and the whole mixture was stirred for 15 min. Then, sat. NaHCO_3_ aq. was slowly added to the reaction solution at 0 °C and extracted with CHCl_3_. The organic layer was dried over Na_2_SO_4_ and was concentrated in vacuo. The crude material was purified by column chromatography (SiO_2_; CHCl_3_/MeOH = 40:1) to afford **21** (52.1 mg, 83%) as white powder.

mp: 83.3–85.6 °C. ${\left[\alpha \right]}_{D}^{23}$ = +43.4° (*c* = 0.99 in CHCl_3_). ^1^H NMR (500 MHz, CDCl_3_) δ: 7.61–7.57 (m, 4H), 7.44–7.40 (m, 2H), 7.38–7.33 (m, 4H), 7.12 (d, *J* = 8.7 Hz, 2H), 7.00 (t, *J* = 5.5 Hz, 1H), 6.81 (d, *J* = 8.7 Hz, 2H), 6.73–6.67 (m, 1H), 5.75 (dd, *J* = 15.2, 1.6 Hz, 1H), 5.68 (d, *J* = 8.3 Hz, 1H), 5.12–5.07 (m, 2H), 4.76–4.72 (m, 1H), 3.77 (s, 3H), 3.74–3.68 (m, 2H), 3.58 (dd, *J* = 10.8, 5.4 Hz, 1H), 3.52 (dd, *J* = 10.8, 5.5 Hz, 1H), 3.50–3.47 (m, 1H), 3.42–3.37 (m, 1H), 3.16 (dd, *J* = 14.4, 5.7 Hz, 1H), 3.03 (dd, *J* = 14.5, 7.6 Hz, 1H), 2.53–2.49 (m, 1H), 2.44–2.38 (m, 3H), 1.97–1.88 (m, 3H), 1.03 (s, 9H), 0.98 (d, *J* = 7.0 Hz, 3H). ^13^C NMR (125 MHz, CDCl_3_) δ: 172.7, 170.9, 170.7, 165.7, 158.6, 142.0, 135.6 (4C), 133.4, 130.3 (2C), 129.9 (2C), 128.7, 127.8 (3C), 127.8 (2C), 125.0, 114.2 (2C), 76.0, 69.7, 64.2, 59.0, 55.3, 54.2, 39.8, 35.8, 35.3, 34.4, 34.0, 32.5, 26.9 (3C), 19.3, 13.6. HR-ESI MS: *m/z* 745.3520, calcd for C_41_H_53_N_2_O_9_Si. Found: 745.3532.

#### 3.3.12. (3*S*,10*R*,16*S*,*E*)-3-(2-((*tert*-Butyldiphenylsilyl)oxy)ethyl)-10-(4-methoxybenzyl)-16-((*S*)-1-((2*R*,3*R*)-3-phenyloxiran-2-yl)ethyl)-1,4-dioxa-8,11-diazacyclohexadec-13-ene-2,5,9,12-tetraone (**22**)

Dess–Martin periodinane (36 mg, 0.085 mmol) was added to a solution of **21** (57.9 mg, 0.078 mmol) in CH_2_Cl_2_ (0.8 mL) at 0 °C, and the whole mixture was stirred at rt for 3 h. Then, Na_2_S_2_O_3_ and NaHCO_3_ solutions were added at 0 °C to stop the reaction and extracted with CHCl_3_. The organic layer was dried over Na_2_SO_4_ and was concentrated in vacuo to give an intermediate aldehyde, which was used for the next reaction without further purification. Phosphazene P_2_-Et (31 mg, 0.092 mmol) in CH_2_Cl_2_ (0.1 mL) was slowly added to a solution of above aldehyde and sulfonium salt **5** (50 mg, 0.12 mmol) in anhydrous CH_2_Cl_2_ (0.51 mL) under N_2_ atmosphere at −78 °C, and the whole mixture was stirred at −78 °C for 3 h. Brine was added to stop the reaction and extracted with CHCl_3_. The organic layer was dried over Na_2_SO_4_ and was concentrated in vacuo. The crude material was purified by column chromatography (SiO_2_; CHCl_3_/MeOH = 100:1) to afford **22** (32.5 mg, 64%) as white powder.

mp: 81.9–83.2 °C. ${\left[\alpha \right]}_{D}^{23}$ = +37.2° (*c* = 0.63 in CHCl_3_). ^1^H NMR (500 MHz, CDCl_3_) δ: 7.61–7.59 (m, 2H), 7.57–7.55 (m, 2H), 7.44–7.29 (m, 9H), 7.24–7.22 (m, 2H), 7.10 (d, *J* = 8.8 Hz, 2H), 6.98 (t, *J* = 5.8 Hz, 1H), 6.81 (d, *J* = 8.7 Hz, 2H), 6.72–6.66 (m, 1H), 5.70 (dd, *J* = 15.1, 1.5 Hz, 1H), 5.55 (d, *J* = 8.2 Hz, 1H), 5.16–5.13 (m, 1H), 5.04 (dd, *J* = 10.1, 3.5 Hz, 1H), 4.77–4.73 (m, 1H), 3.77 (s, 3H), 3.67 (d, *J* = 2.0 Hz, 1H), 3.63–3.60 (m, 2H), 3.53–3.48 (m, 1H), 3.35–3.31 (m, 1H), 3.14 (dd, *J* = 14.4, 5.7 Hz, 1H), 3.03 (dd, *J* = 14.4, 7.6 Hz, 1H), 2.90 (dd, *J* = 7.8, 2.1 Hz, 1H), 2.57–2.53 (m, 1H), 2.47–2.33 (m, 3H), 1.79–1.73 (m, 3H), 1.14 (d, *J* = 6.9 Hz, 3H), 1.03 (s, 9H). ^13^C NMR (125 MHz, CDCl_3_) δ: 172.9, 170.8, 165.4, 158.7, 141.2, 136.8, 135.6 (4C), 133.3, 130.3 (2C), 130.0, 129.9, 128.8 (2C), 128.6, 128.5, 127.9 (3C), 127.8 (3C), 125.7 (2C), 125.2, 114.2 (2C), 76.2, 69.6, 63.4, 59.4, 58.8, 55.3, 54.1, 40.9, 36.8, 35.3, 34.3, 33.8, 32.4, 27.0 (3C), 19.3, 13.9. HR-ESI MS: *m/z* 833.3833, calcd for C_48_H_57_N_2_O_9_Si. Found: 833.3831.

#### 3.3.13. 2-((3*S*,10*R*,16*S*,*E*)-10-(4-Methoxybenzyl)-2,5,9,12-tetraoxo-16-((*S*)-1-((2*R*,3*R*)-3-phenyloxiran-2-yl)ethyl)-1,4-dioxa-8,11-diazacyclohexadec-13-en-3-yl)ethyl 4-methylbenzenesulfonate (**24**)

Tetra-*n*-butylammonium fluoride (*ca*. 1 mol/L in THF, 0.22 mL, 0.22 mmol) and acetic acid (13.4 mg, 0.22 mmol) were added to a solution of **22** (61.9 mg, 0.074 mmol) in THF (7.4 mL) under N_2_ atmosphere at 0 °C, and the whole mixture was stirred for 5 h. Then, sat. NH_4_Cl aq. was added to the reaction solution at 0 °C and extracted with CHCl_3_. The organic layer was dried over Na_2_SO_4_ and was concentrated in vacuo. The crude material containing **23** was dissolved in CH_2_Cl_2_ (1.4 mL) under N_2_ atmosphere, and *p*-toluenesulfonyl chloride (15.4 mg, 0.081 mmol) and pyridine (12.8 mg, 0.16 mmol) were added at 0 °C. The whole mixture was stirred overnight at rt, then sat. NaHCO_3_ aq. was added to the reaction solution at 0 °C and extracted with CHCl_3_. The organic layer was dried over Na_2_SO_4_ and was concentrated in vacuo. The crude material was purified by column chromatography (SiO_2_; CHCl_3_/MeOH = 90:1) to afford **24** (24.1 mg, 44% in 2 steps) as a colorless amorphous solid.

${\left[\alpha \right]}_{D}^{23}$ = +45.2° (*c* = 0.32 in CHCl_3_). ^1^H NMR (500 MHz, CDCl_3_) δ: 7.73 (d, *J* = 8.5 Hz, 2H), 7.34–7.30 (m, 5H), 7.24–7.22 (m, 2H), 7.10 (d, *J* = 8.8 Hz, 2H), 6.93 (t, *J* = 5.8 Hz, 1H), 6.81 (d, *J* = 8.7 Hz, 2H), 6.69–6.63 (m, 1H), 5.68 (dd, *J* = 15.1, 1.6 Hz, 1H), 5.55 (d, *J* = 8.2 Hz, 1H), 5.12–5.08 (m, 1H), 4.90 (t, *J* = 6.7 Hz, 1H), 4.74–4.70 (m, 1H), 3.93–3.86 (m, 2H), 3.77 (s, 3H), 3.66 (d, *J* = 2.0 Hz, 1H), 3.51–3.45 (m, 1H), 3.36–3.30 (m, 1H), 3.14 (dd, *J* = 14.6, 5.7 Hz, 1H), 3.01 (dd, *J* = 14.5, 7.6 Hz, 1H), 2.87 (dd, *J* = 7.9, 2.0 Hz, 1H), 2.61–2.57 (m, 1H), 2.44 (s, 3H), 2.41–2.34 (m, 3H), 1.79–1.74 (m, 3H), 1.13 (d, *J* = 6.9 Hz, 3H). ^13^C NMR (125 MHz, CDCl_3_) δ: 172.3, 170.8, 169.6, 165.4, 158.7, 145.3, 141.1, 136.7, 132.5, 130.3 (2C), 130.0 (2C), 128.8 (2C), 128.7, 128.5, 128.1 (2C), 125.8 (2C), 125.3, 114.2 (2C), 76.7, 68.5, 64.9, 63.6, 59.4, 55.3, 54.3, 40.8, 36.9, 35.3, 34.1, 32.3, 29.9, 21.8, 13.9. HR-ESI MS: *m/z* 749.2744, calcd for C_39_H_45_N_2_O_11_S. Found: 749.2746.

#### 3.3.14. (3*S*,10*R*,16*S*,*E*)-3-(2-(Ethylthio)ethyl)-10-(4-methoxybenzyl)-16-((*S*)-1-((2*R*,3*R*)-3-phenyloxiran-2-yl)ethyl)-1,4-dioxa-8,11-diazacyclohexadec-13-ene-2,5,9,12-tetraone (**25**)

Sodium hydride (144.5 mg, 6.02 mmol) was added to a solution of ethanethiol (0.67 mL, 9.03 mmol) in THF (10 mL) under N_2_ atmosphere, and the whole mixture was stirred for 1 h to prepare sodium ethanethiolate solution. An aliquot of the thiolate solution (73.6 μL) was added to a solution of **24** (11.9 mg, 0.016 mmol) in anhydrous DMF (0.54 mL) at 0 °C, and the whole mixture was stirred for 4 h. Then, H_2_O was added to the reaction mixture at 0 °C and extracted with CHCl_3_/MeOH = 4/1. The organic layer was dried over Na_2_SO_4_ and was concentrated in vacuo. The crude material was purified by reversed-phase HPLC (Cholester; CH_3_CN/H_2_O = 6:4) to afford **25** (4.95 mg, 49%) as white powder.

mp: 235.1–237.0 °C. ${\left[\alpha \right]}_{D}^{23}$ = +40.7° (*c* = 0.38 in CHCl_3_). ^1^H NMR (500 MHz, CDCl_3_) δ: 7.38–7.33 (m, 3H), 7.24–7.23 (m, 2H), 7.09 (d, *J* = 8.8 Hz, 2H), 6.97 (t, *J* = 5.5 Hz, 1H), 6.80 (d, *J* = 8.7 Hz, 2H), 6.72–6.66 (m, 1H), 5.69 (d, *J* = 15.2 Hz, 1H), 5.56 (d, *J* = 8.0 Hz, 1H), 5.17 (dd, *J* = 11.4, 5.0 Hz, 1H), 4.99 (dd, *J* = 9.6, 2.6 Hz, 1H), 4.75–4.71 (m, 1H), 3.77 (s, 3H), 3.68 (s, 1H), 3.56–3.51 (m, 1H), 3.42–3.37 (m, 1H), 3.13 (dd, *J* = 14.2, 6.1 Hz, 1H), 3.02 (dd, *J* = 14.2, 7.4 Hz, 1H), 2.90 (d, *J* = 7.5 Hz, 1H), 2.58–2.52 (m, 4H), 2.49–2.41 (m, 4H), 1.99–1.92 (m, 1H), 1.83–1.77 (m, 2H), 1.22 (t, *J* = 7.4 Hz, 3H), 1.13 (d, *J* = 6.9 Hz, 3H). ^13^C NMR (125 MHz, CDCl_3_) δ: 172.8, 170.8, 170.0, 165.4, 158.7, 141.2, 136.8, 130.3 (2C), 128.8 (2C), 128.7, 128.5, 125.6 (2C), 125.3, 114.2 (2C), 76.3, 71.3, 63.3, 59.0, 55.3, 54.1, 40.6, 36.8, 35.3, 34.3, 32.5, 30.9, 27.1, 26.0, 14.8, 13.7. HR-ESI MS: *m/z* 639.2740, calcd for C_34_H_43_N_2_O_8_S. Found: 639.2719.

#### 3.3.15. *N*-(2-((2-((3*S*,10*R*,16*S*,*E*)-10-(4-Methoxybenzyl)-2,5,9,12-tetraoxo-16-((*S*)-1-((2*R*,3*R*)-3-phenyloxiran-2-yl)ethyl)-1,4-dioxa-8,11-diazacyclohexadec-13-en-3-yl)ethyl)thio)ethyl)acetamide (**26**)

Sodium hydride (17.3 mg, 0.72 mmol) was added to a solution of *N*-acetylcysteamine (111.5 mg, 0.94 mmol) in DMF (3.6 mL) under N_2_ atmosphere, and the whole mixture was stirred for 1 h to prepare the corresponding thiolate solution. An aliquot of the thiolate solution (0.14 mL) was added to a solution of **24** (10.2 mg, 0.014 mmol) in anhydrous DMF (0.38 mL) at 0 °C, and the whole mixture was stirred for 4 h. Then, H_2_O was added to the reaction solution at 0 °C and extracted with CHCl_3_/MeOH = 4:1. The organic layer was dried over Na_2_SO_4_ and was concentrated in vacuo. The crude material was purified by reversed-phase HPLC (Cholester; CH_3_CN/H_2_O = 4:6) to afford **26** (6.82 mg, 72%) as white powder.

mp: 203.0–204.5 °C. ${\left[\alpha \right]}_{D}^{23}$ = +42.7° (*c* = 0.58 in CHCl_3_/MeOH = 5:1). ^1^H NMR (500 MHz, CDCl_3_) δ: 7.38–7.32 (m, 3H), 7.24–7.23 (m, 2H), 7.09 (d, *J* = 8.8 Hz, 2H), 6.95 (t, *J* = 5.9 Hz, 1H), 6.80 (d, *J* = 8.7 Hz, 2H), 6.72–6.66 (m, 1H), 5.81 (s, 1H), 5.69 (d, *J* = 15.2 Hz, 1H), 5.61 (d, *J* = 8.2 Hz, 1H), 5.18–5.14 (m, 1H), 4.97 (dd, *J* = 9.2, 3.6 Hz, 1H), 4.74–4.70 (m, 1H), 3.76 (s, 3H), 3.68 (d, *J* = 2.2 Hz, 1H), 3.55–3.50 (m, 1H), 3.44–3.40 (m, 1H), 3.37 (dd, *J* = 12.7, 6.5 Hz, 2H), 3.13 (dd, *J* = 14.5, 5.8 Hz, 1H), 3.01 (dd, *J* = 14.5, 7.6 Hz, 1H), 2.92 (dd, *J* = 7.2, 2.1 Hz, 1H), 2.60–2.53 (m, 6H), 2.45–2.38 (m, 2H), 1.95–1.89 (m, 4H), 1.86–1.79 (m, 2H), 1.12 (d, *J* = 6.9 Hz, 3H). ^13^C NMR (125 MHz, CDCl_3_) δ: 172.7, 170.8, 170.3, 169.9, 165.5, 158.6, 141.2, 136.8, 130.3 (2C), 128.9 (2C), 128.7, 128.5, 125.7 (2C), 125.3, 114.2 (2C), 76.4, 71.1, 63.3, 58.8, 55.3, 54.2, 40.4, 38.5, 36.9, 35.2, 34.2, 32.5, 31.9, 30.9, 27.0, 23.4, 13.6. HR-ESI MS: *m/z* 696.2955, calcd for C_36_H_46_N_3_O_9_S. Found: 696.2950.

## 4. Conclusions

In summary, a short-step stereospecific total synthesis of arenastatin A (**1**) was accomplished in this study. The convergent assembly of four segments, deprotection-triggered macrocyclization, and the stereoselective Corey–Chaykovsky reaction yielded the natural product in the longest linear sequence of seven steps, starting with a known compound. In addition, the preparation of some segment B analogs using a late-stage functionalization approach was successful. As other diversification methods mediated by thiols have been reported [39], further synthesis and evaluation of various analogs will lead to the development of more potent and selective anticancer drug candidates, which will be undertaken in due course.

## Figures and Tables

**Table 1:** Cytotoxicity of arenastatin A (**1**) and analogs against cancer cells.

Compound	IC_50_ (nM) ^1^
**1**	0.14
**25**	1.8
**26**	16.0

^1^ IC_50_ against KB3-1 cells.

## Data Availability

Data are contained within the article and Supplementary Materials.

## Supplementary Materials

The following supporting information can be downloaded from https://www.mdpi.com/article/10.3390/molecules29174058/s1. Supplementary Data S1: NMR spectra of the new compounds.
