# Supplementary material for: Concise and Stereospecific Total Synthesis of Arenastatin A and Its Segment B Analogs
Source: Molecules. 2024 Aug 27;29(17):4058. doi: 10.3390/molecules29174058 (PMC11396571; doi:10.3390/molecules29174058)

# Supporting Information

## Concise and Stereospecific Total Synthesis of Arenastatin A and Its Segment B Analogs

Yurina Mihara, Haruki Kadoya, Soki Kakihana and Naoyuki Kotoku *

College of Pharmaceutical Sciences, Ritsumeikan University, 1-1-1 Noji-Higashi,
Kusatsu, Shiga 525-8577, Japan;

Contents

$^{1}H$ NMR, $^{13}C$ NMR, and HRMS spectra for new compounds: pages S2-S31

**$^{1}$H NMR** (500 MHz, DMSO-$d_6$)

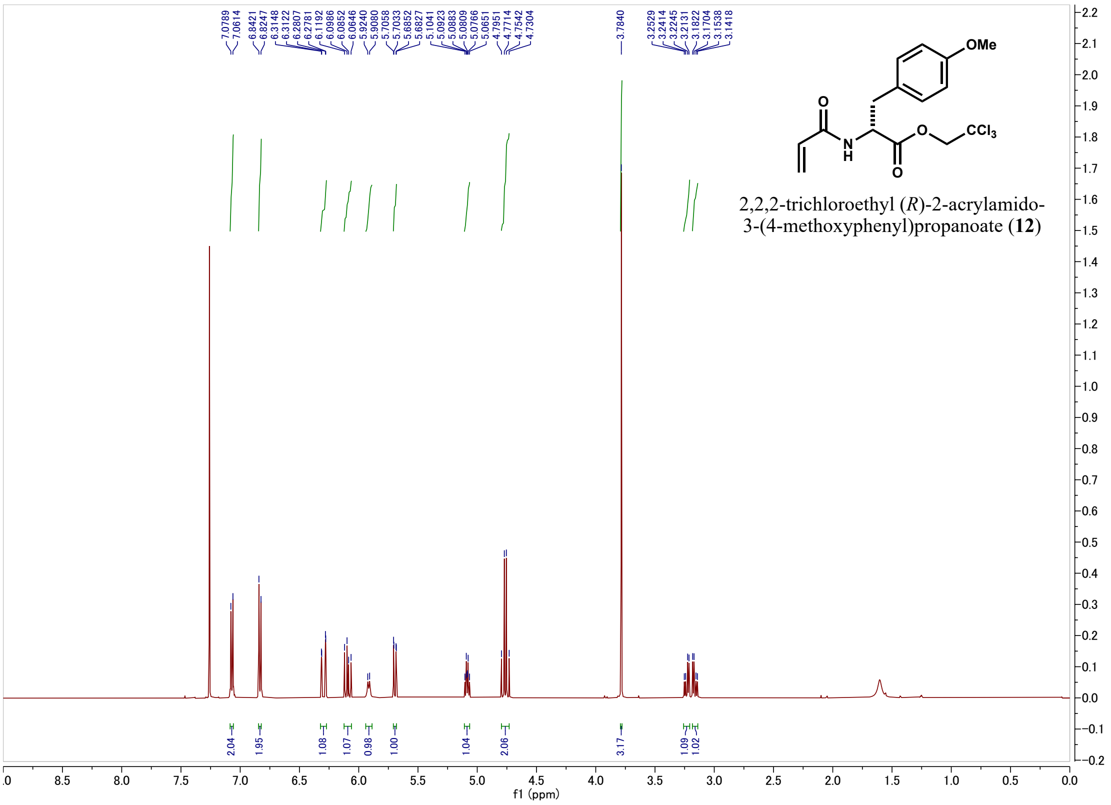


2,2,2-trichloroethyl (*R*)-2-acrylamido-3-(4-methoxyphenyl)propanoate (**12**)

**$^{13}$C NMR** (125 MHz, $CDCl_3$)

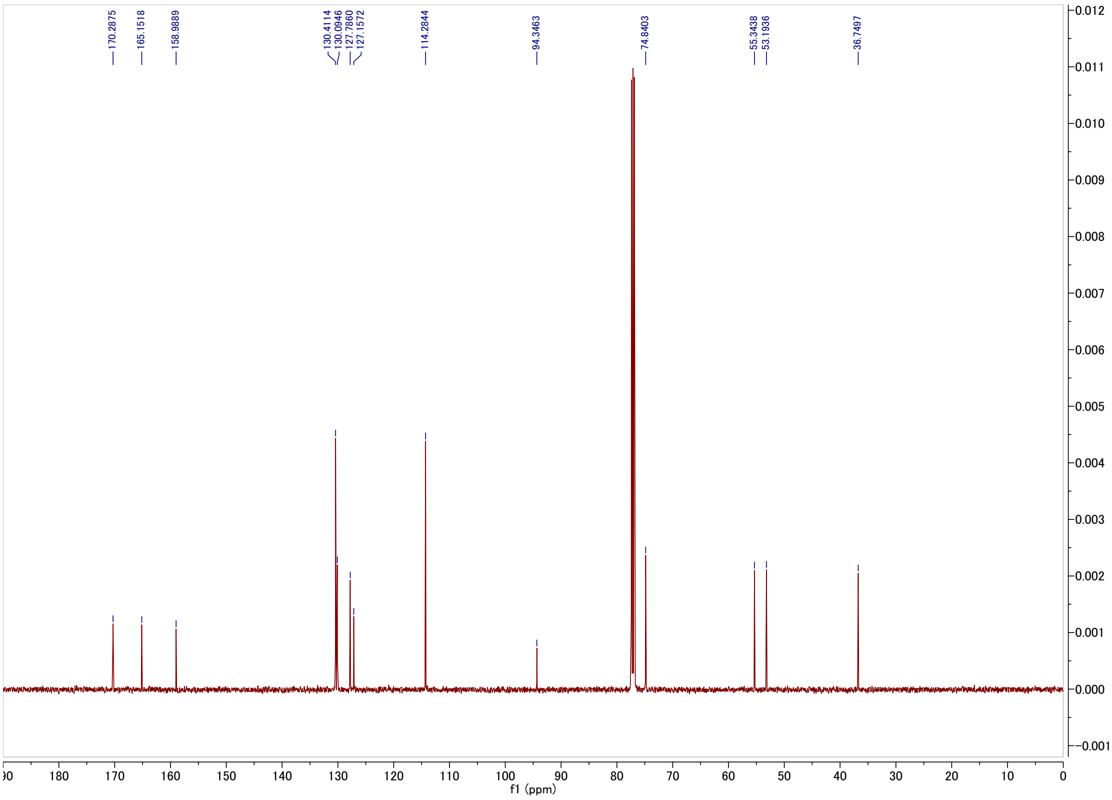

HRMS spectrum of **12**

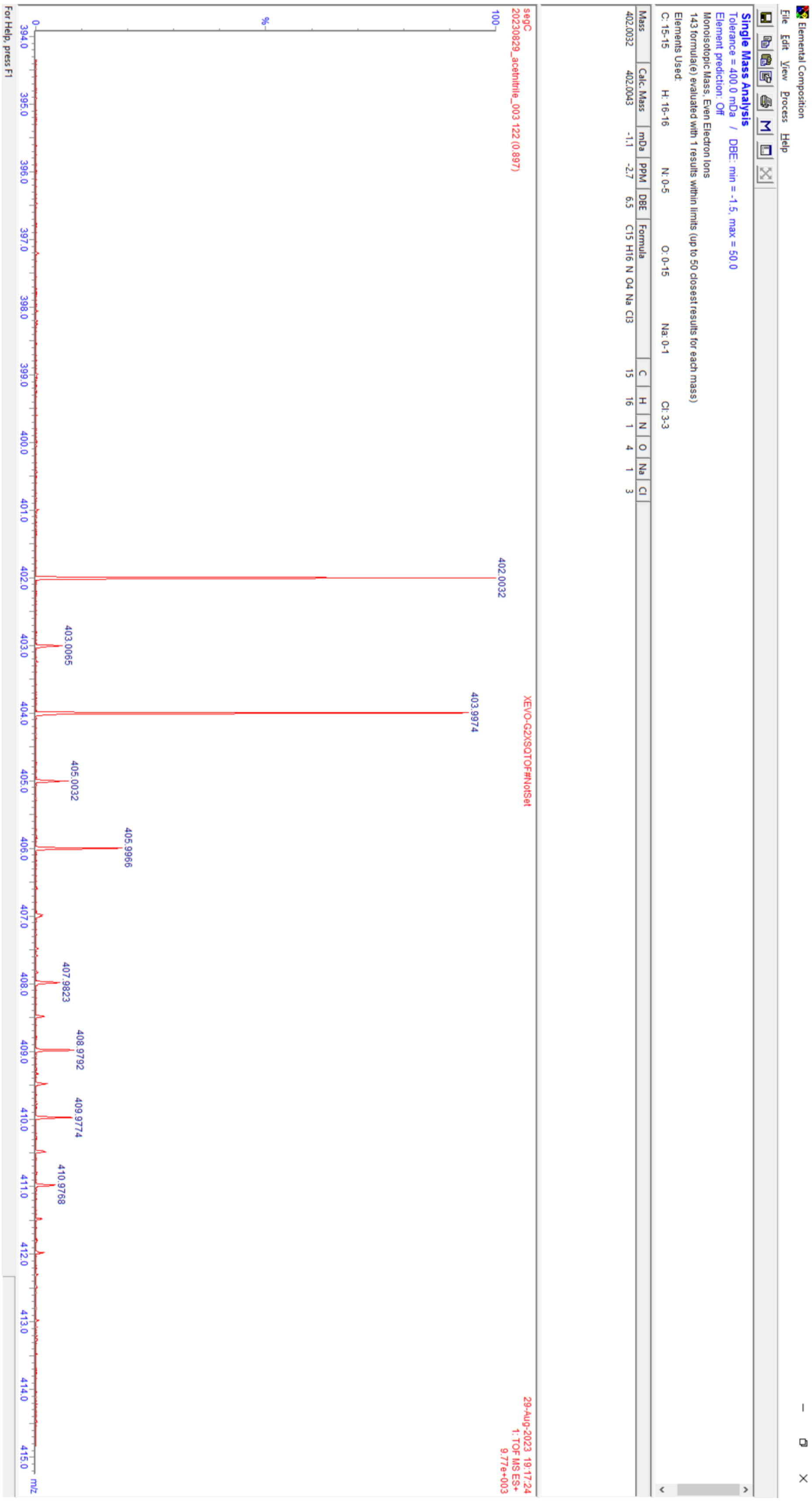

**$^{1}$H NMR** (500 MHz, DMSO-$d_6$)

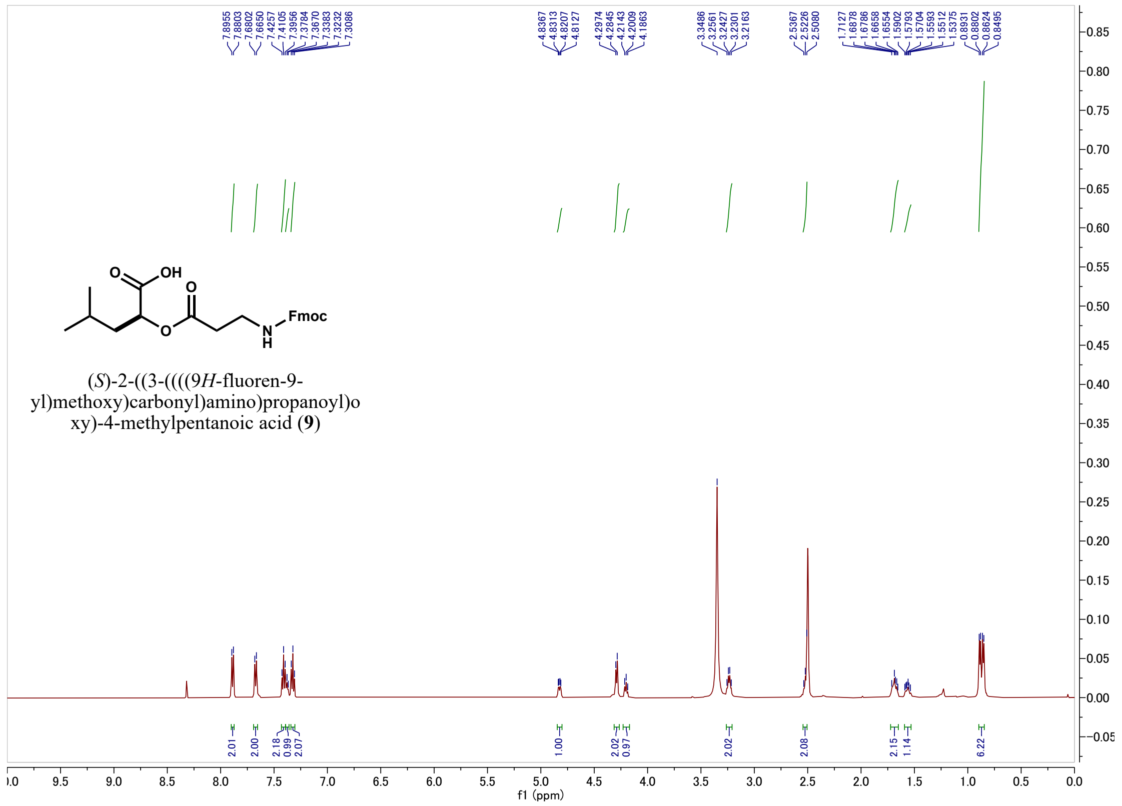

(*S*)-2-((3-((((9*H*-fluoren-9-yl)methoxy)carbonyl)amino)propanoyl)oxy)-4-methylpentanoic acid (**9**)

**$^{13}$C NMR** (125 MHz, $CDCl_3$)

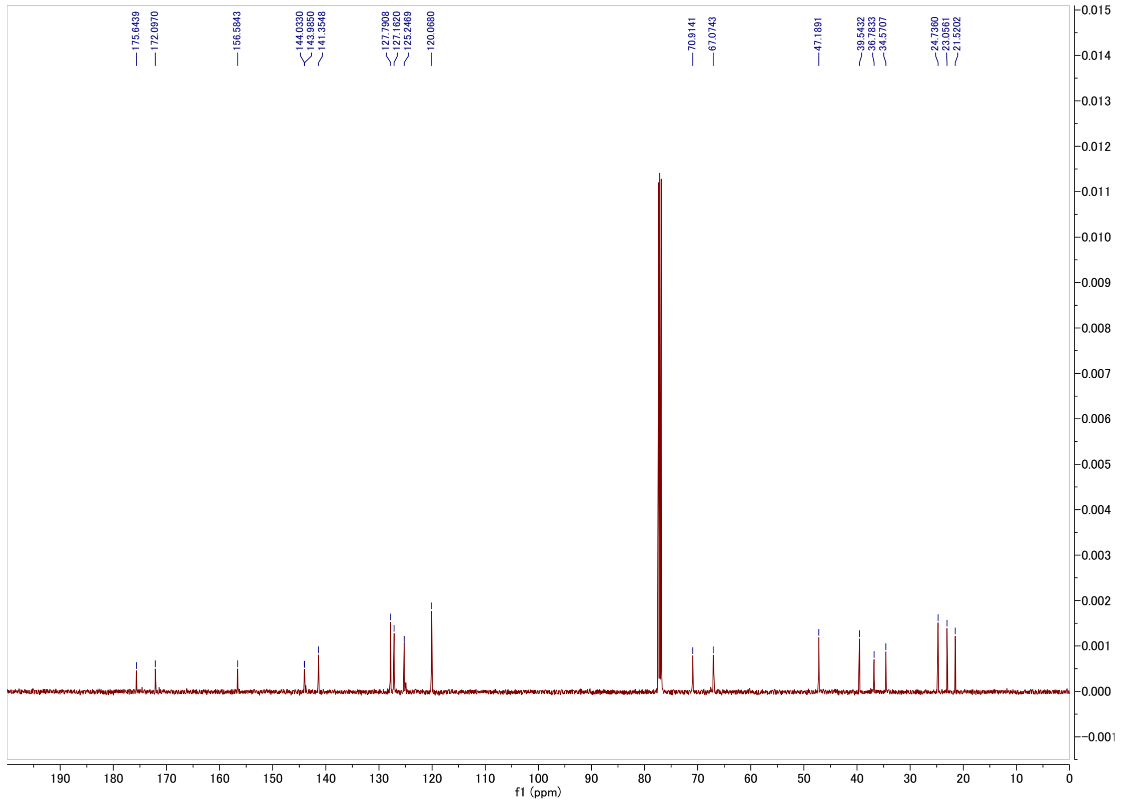

HRMS spectrum of **9**

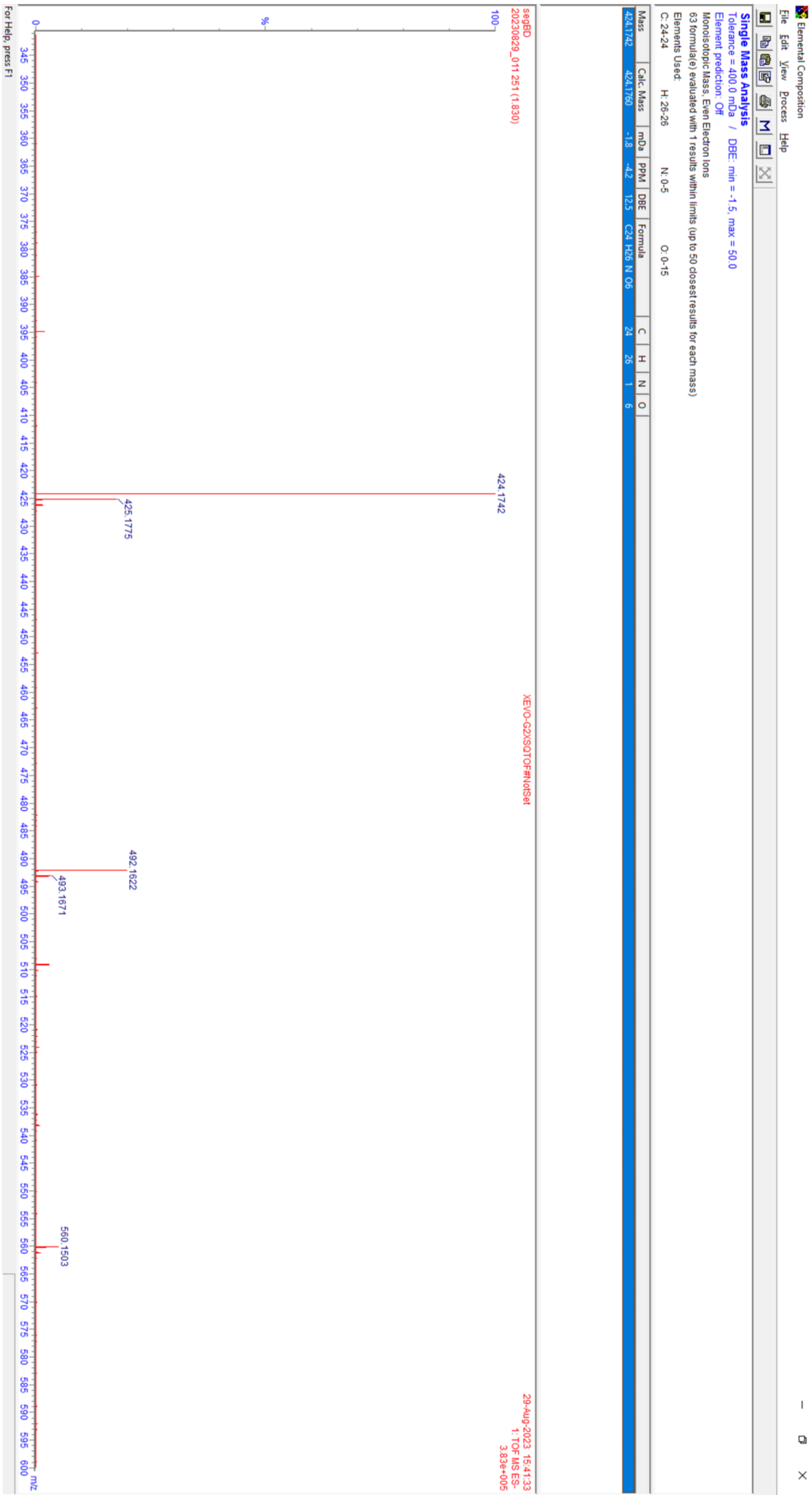

**$^{1}$H NMR** (500 MHz, $CDCl_3$)

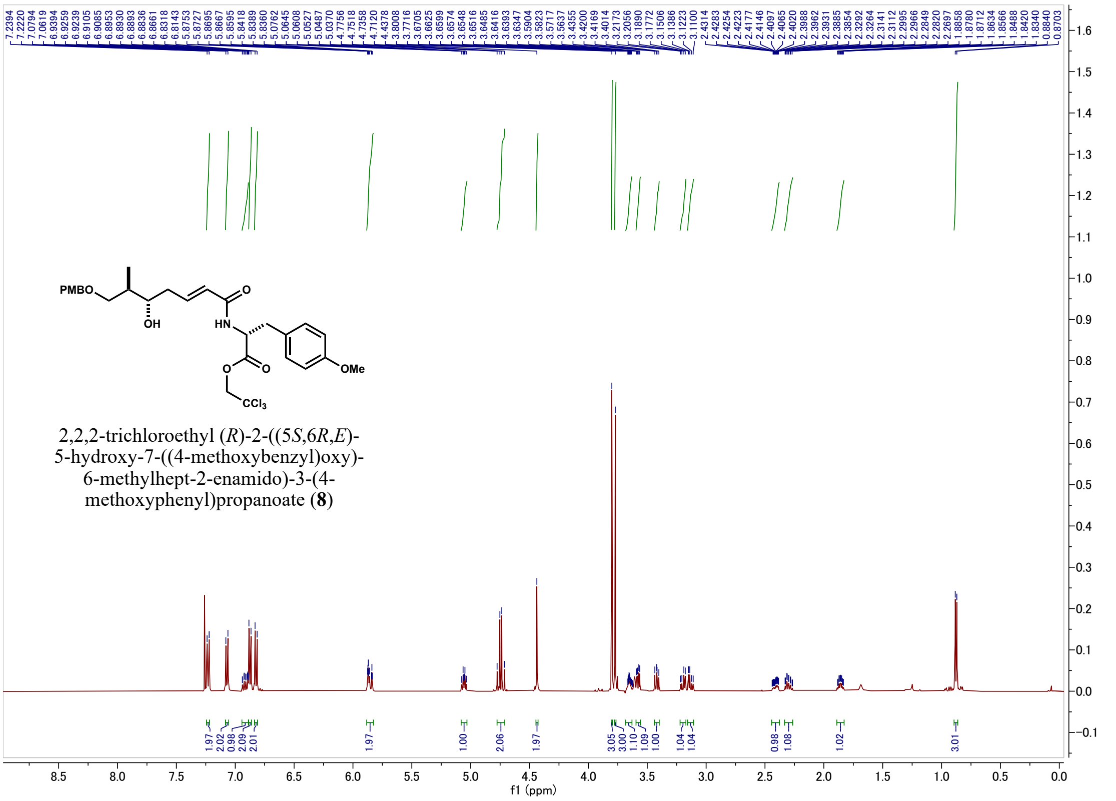

2,2,2-trichloroethyl (*R*)-2-((5*S*,6*R*,*E*)-5-hydroxy-7-((4-methoxybenzyl)oxy)-6-methylhept-2-enamido)-3-(4-methoxyphenyl)propanoate (**8**)

**$^{13}$C NMR** (125 MHz, $CDCl_3$)

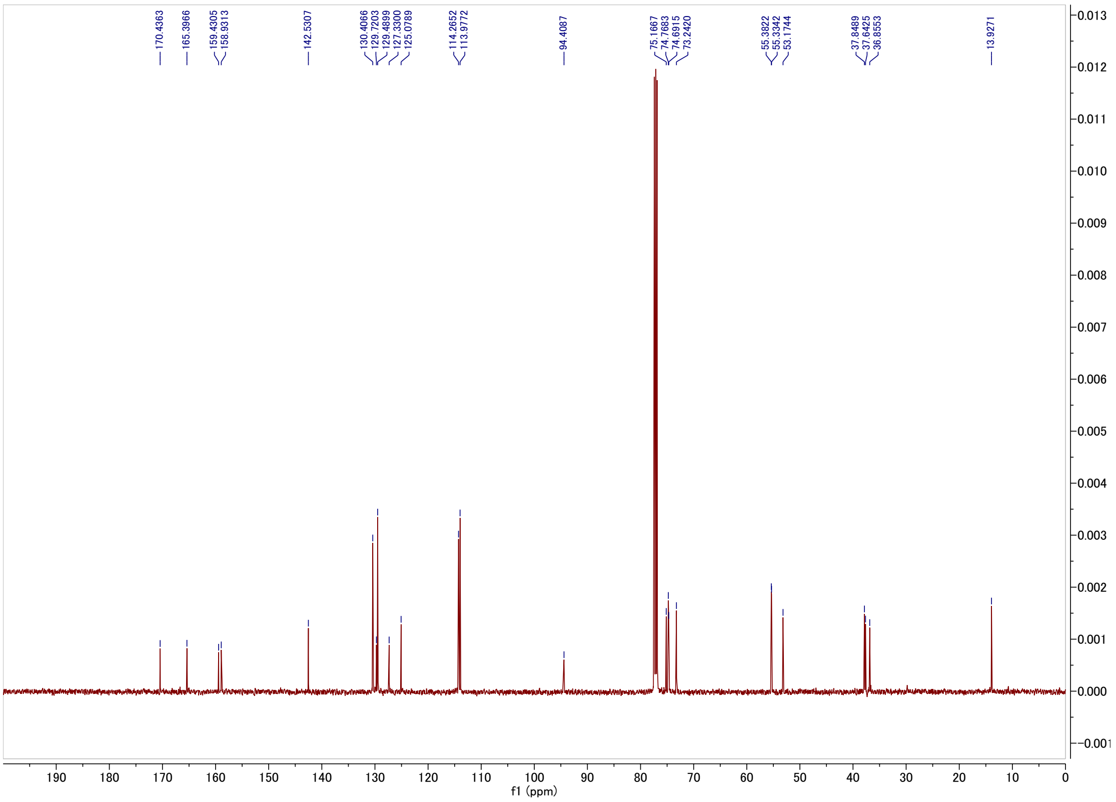

HRMS spectrum of **8**

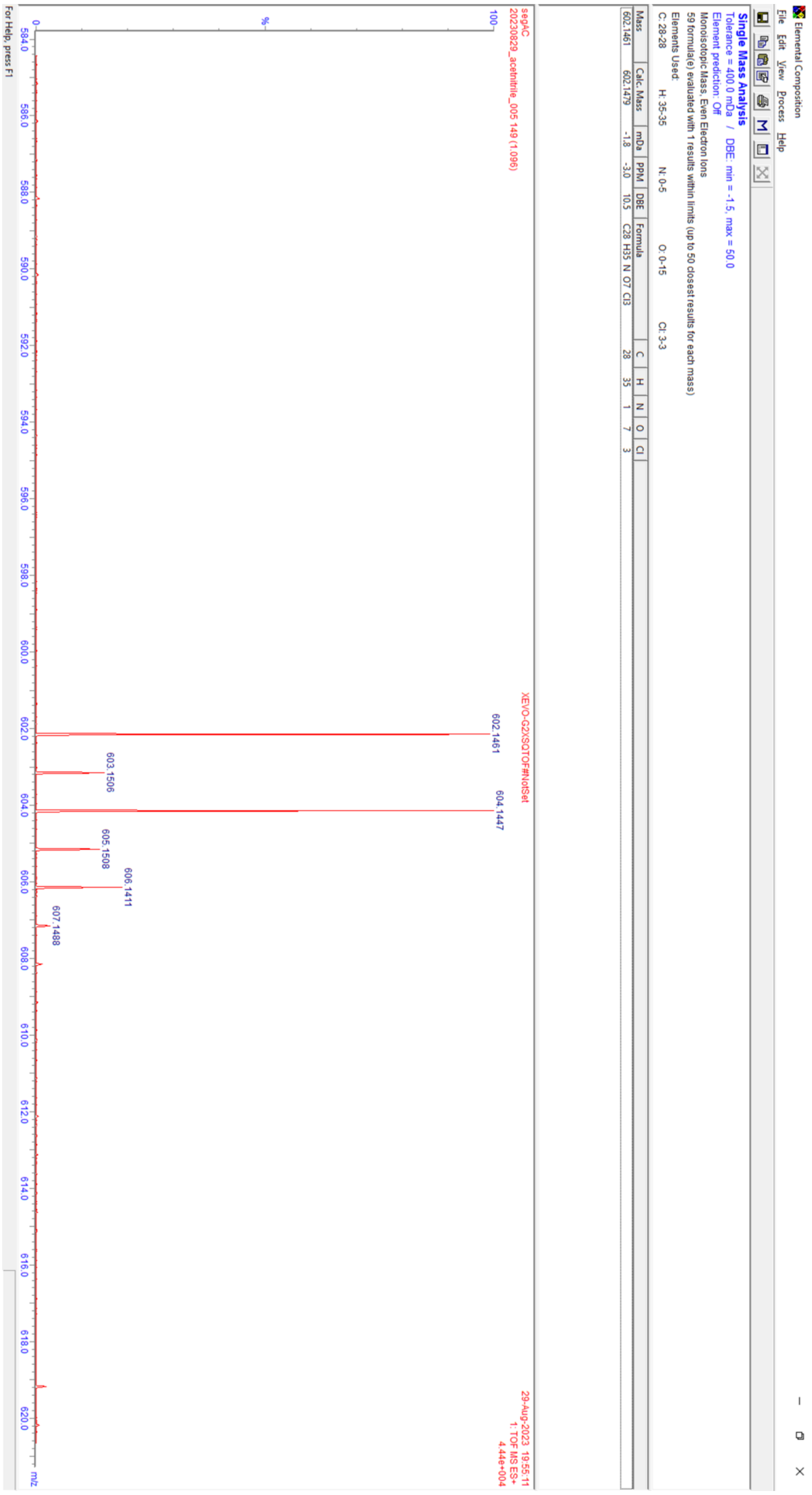

**$^{1}$H NMR** (500 MHz, $CDCl_3$)

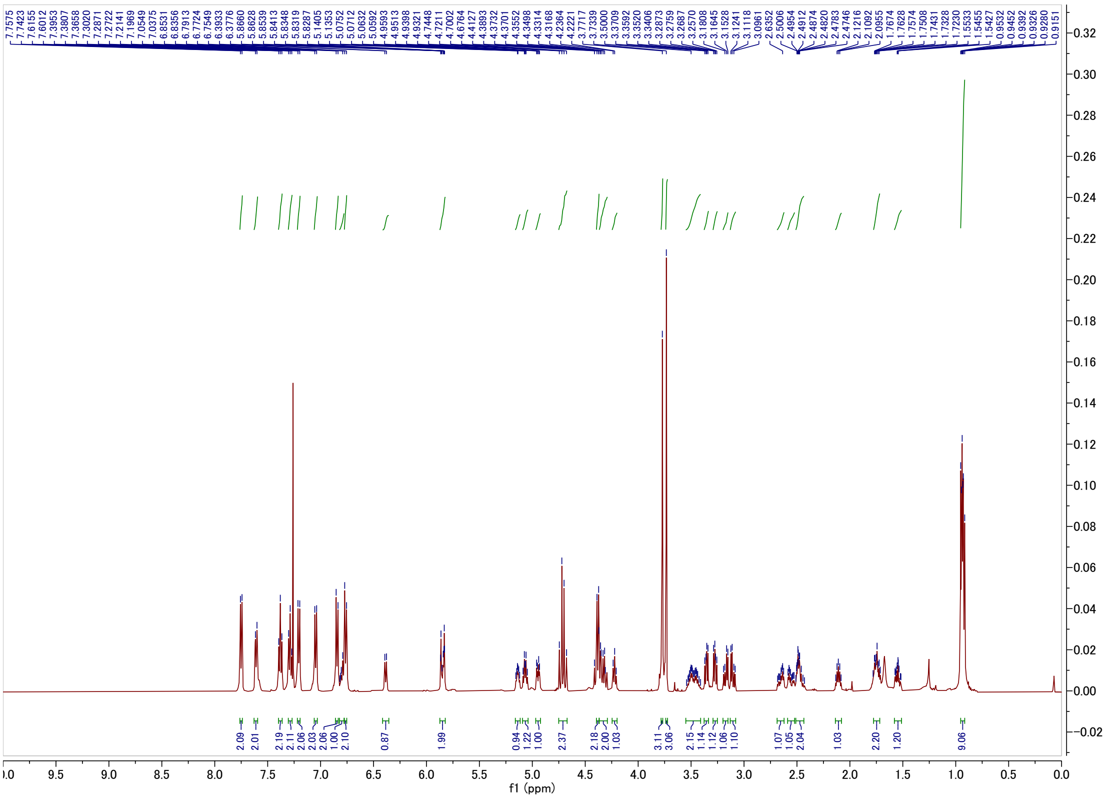


**$^{13}$C NMR** (125 MHz, $CDCl_3$)

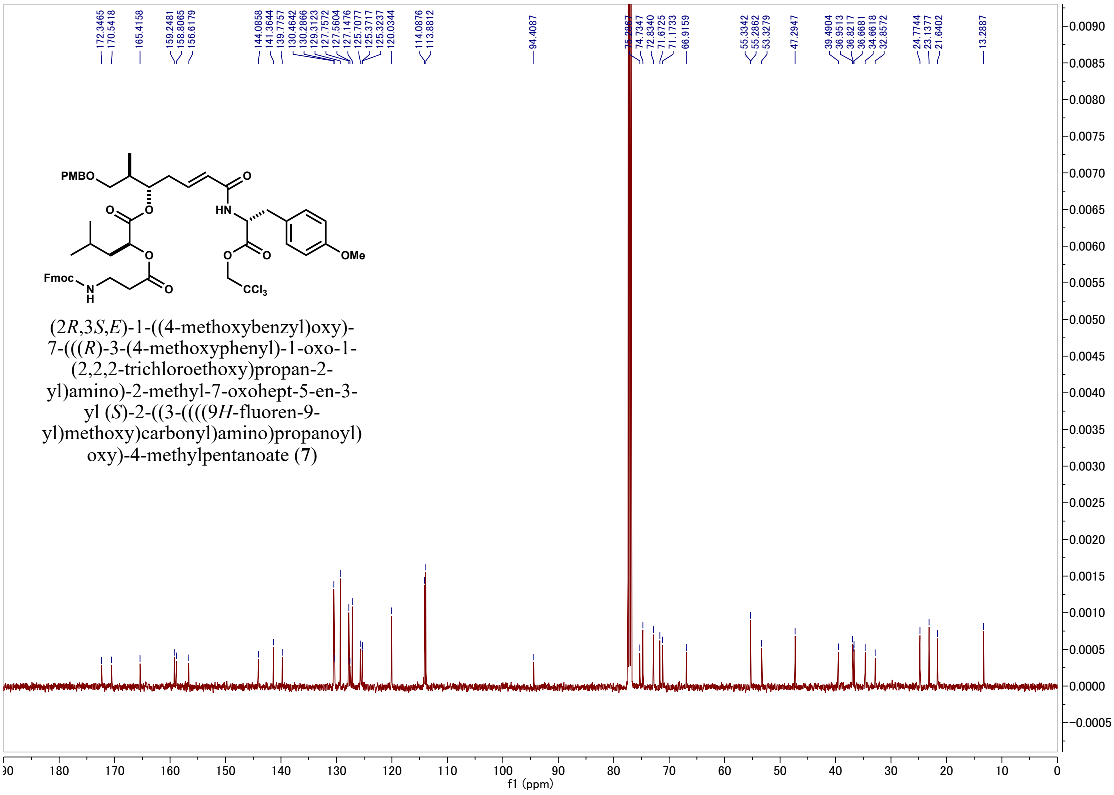


(2*R*,3*S*,*E*)-1-((4-methoxybenzyl)oxy)-7-(((*R*)-3-(4-methoxyphenyl)-1-oxo-1-(2,2,2-trichloroethoxy)propan-2-yl)amino)-2-methyl-7-oxohept-5-en-3-yl (*S*)-2-((3-((((9*H*-fluoren-9-yl)methoxy)carbonyl)amino)propanoyl)oxy)-4-methylpentanoate (**7**)

HRMS spectrum of **7**

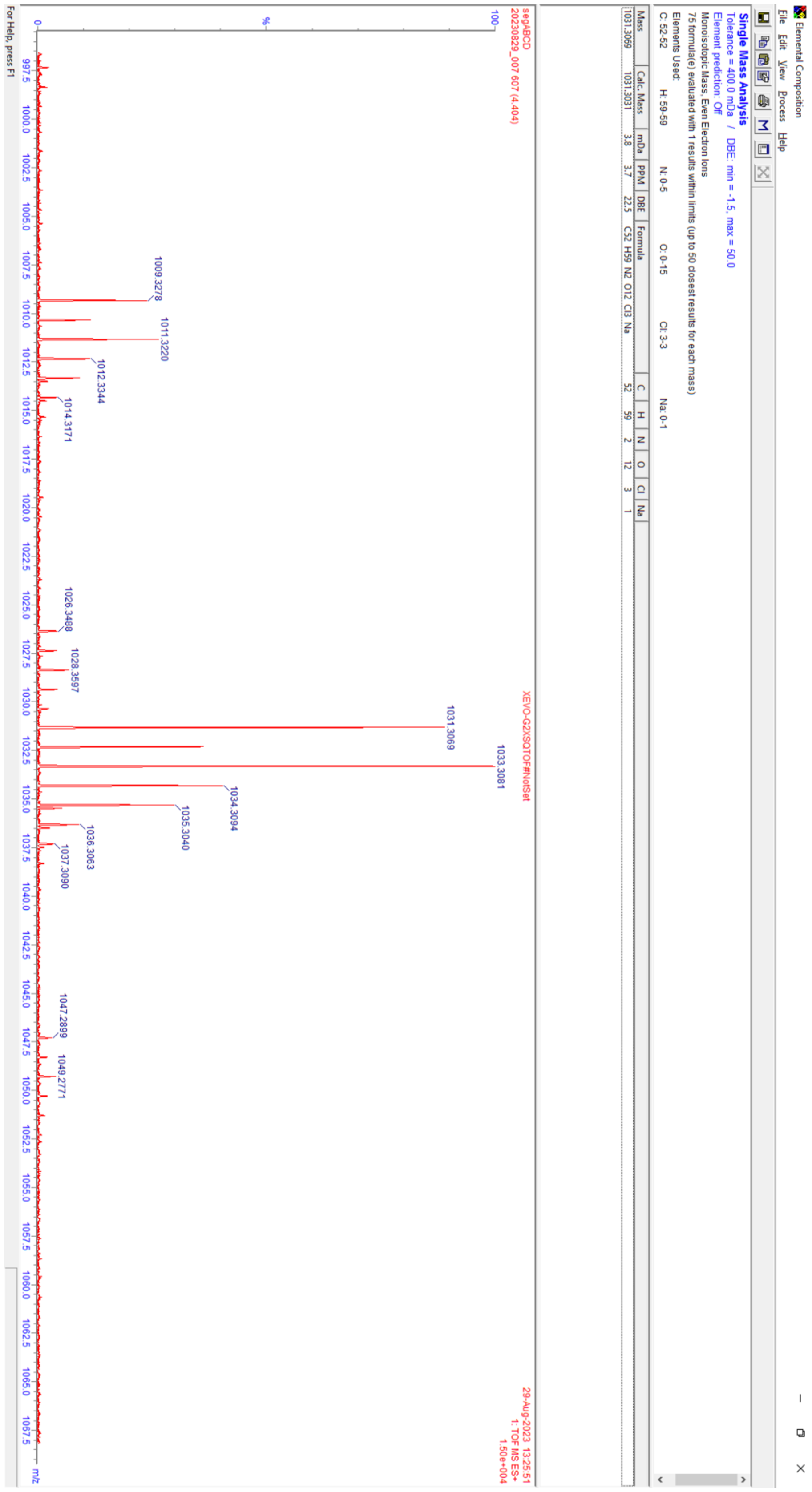

**$^{1}$H NMR** (500 MHz, $CDCl_3$)

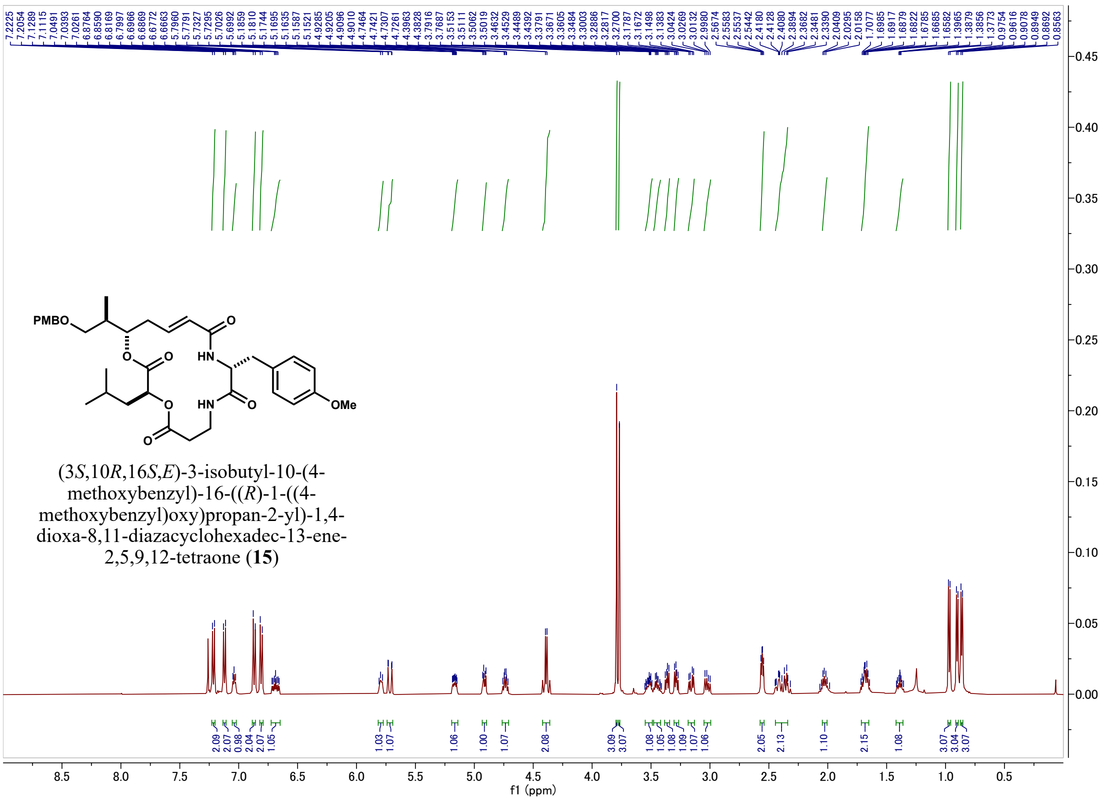


**$^{13}$C NMR** (125 MHz, $CDCl_3$)

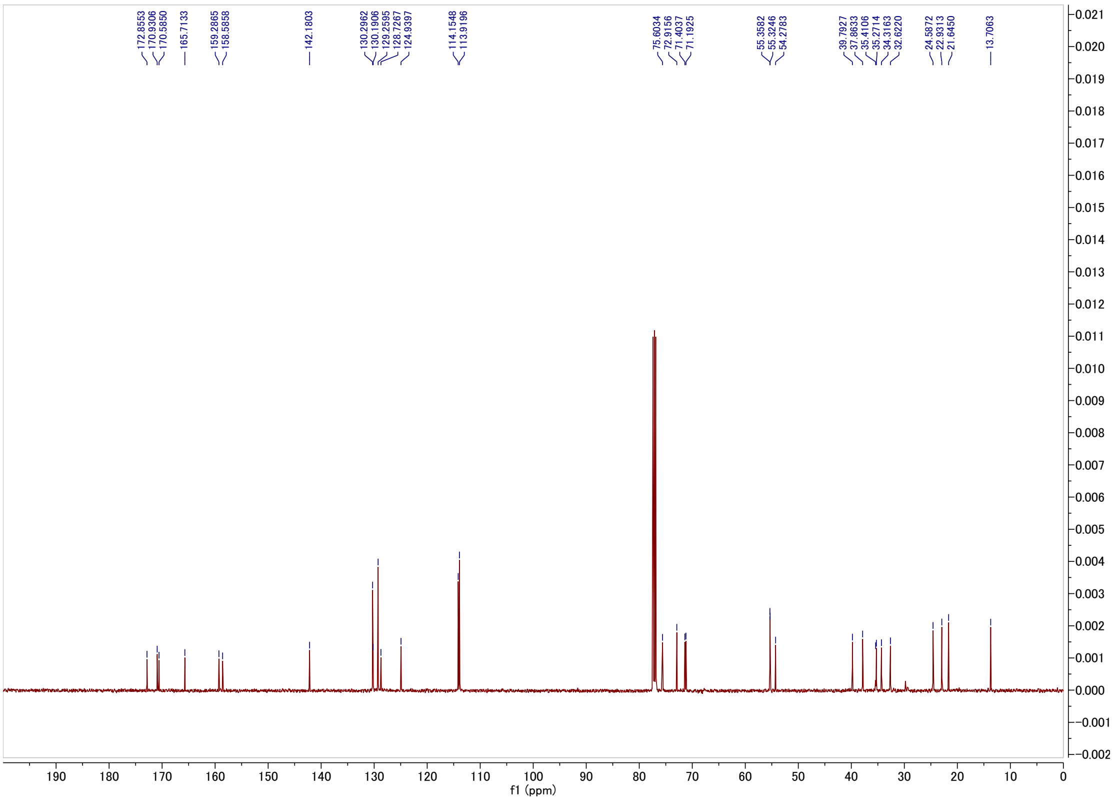

HRMS spectrum of **15**

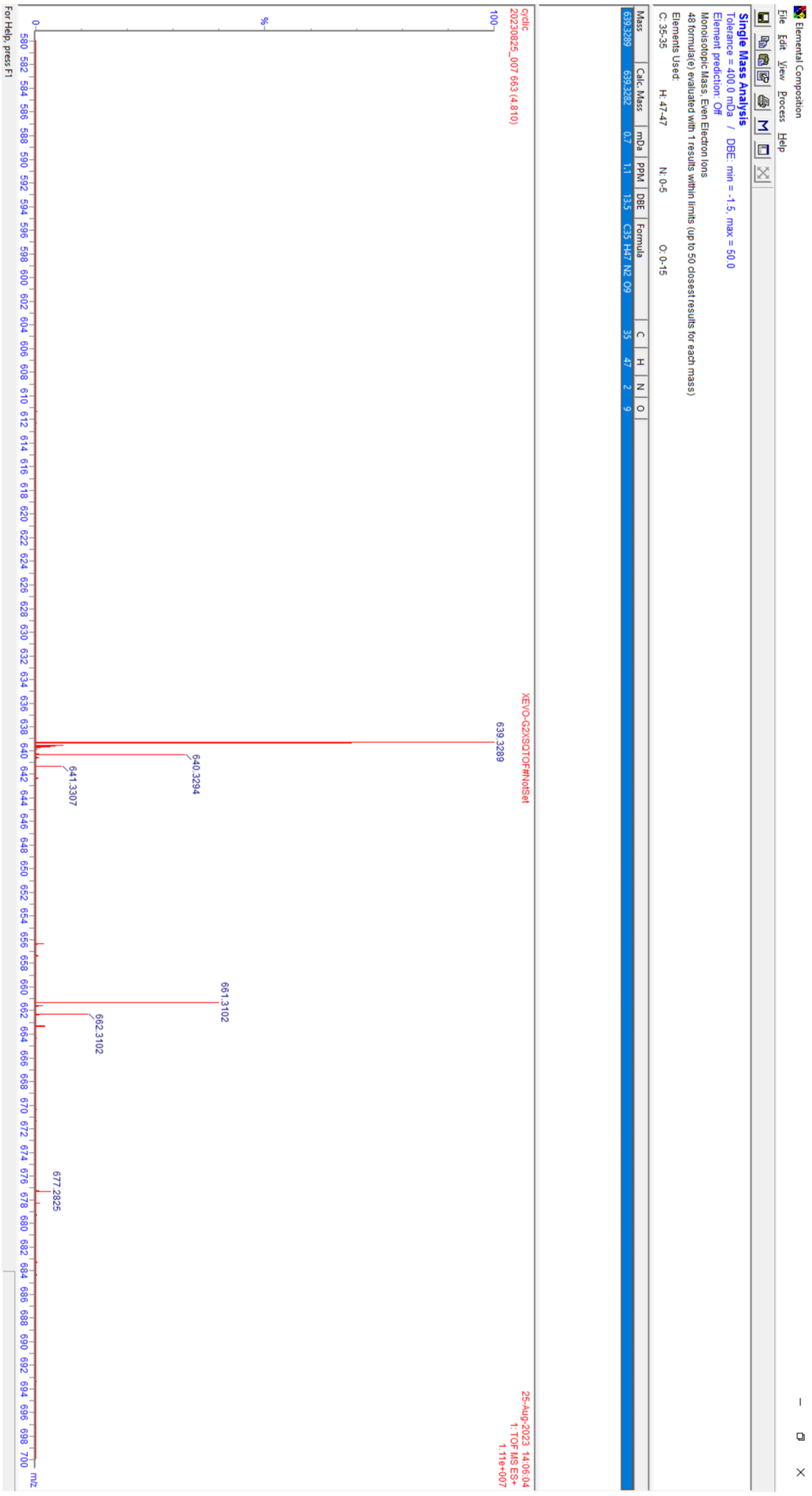

**$^{1}$H NMR** (500 MHz, $CDCl_3$)

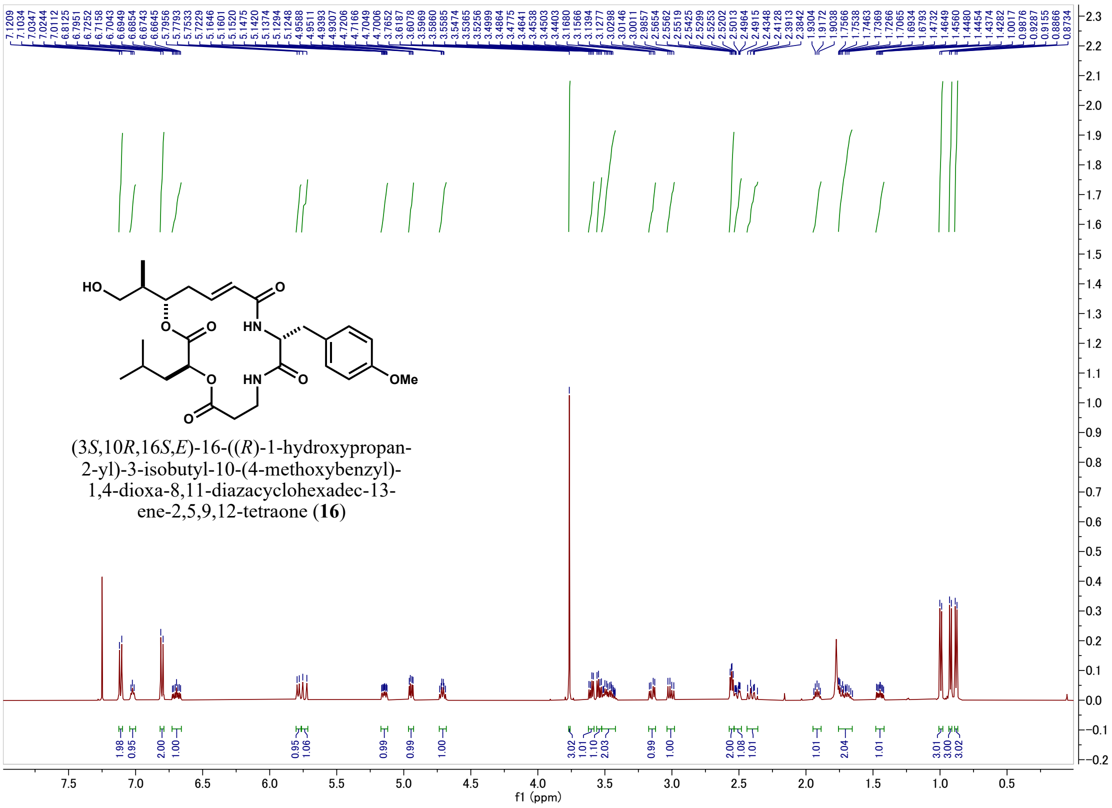


**$^{13}$C NMR** (125 MHz, $CDCl_3$)

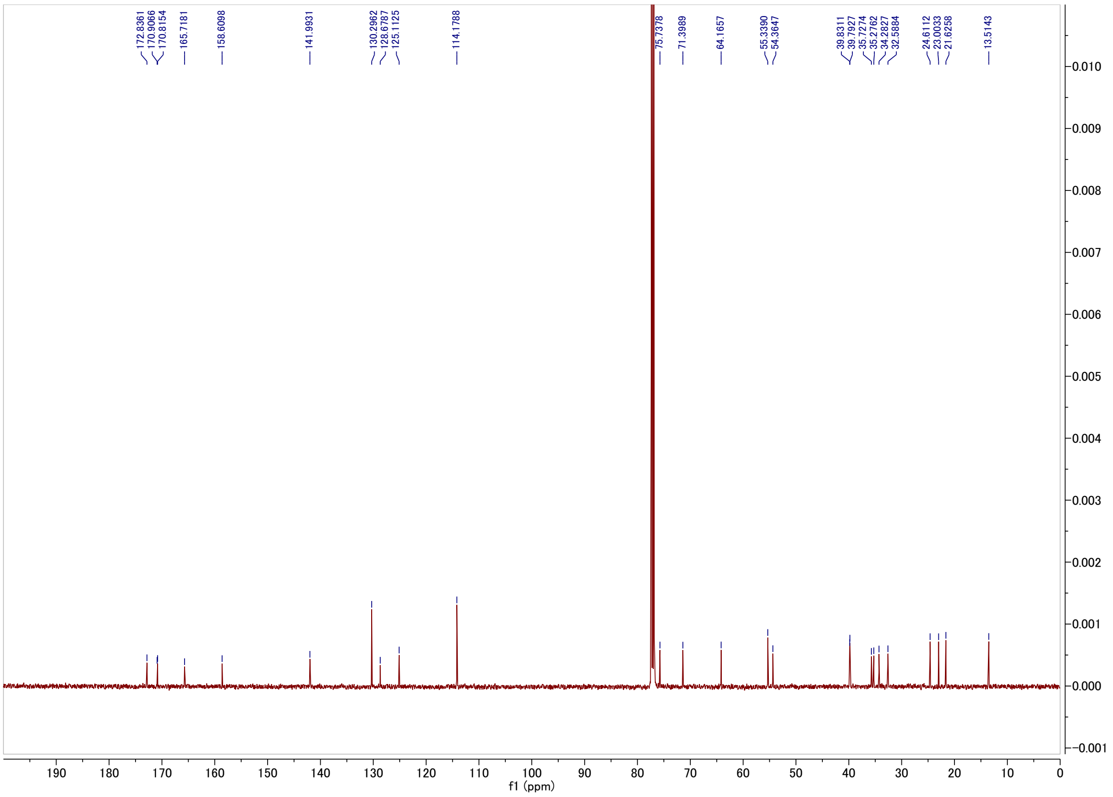

HRMS spectrum of **16**

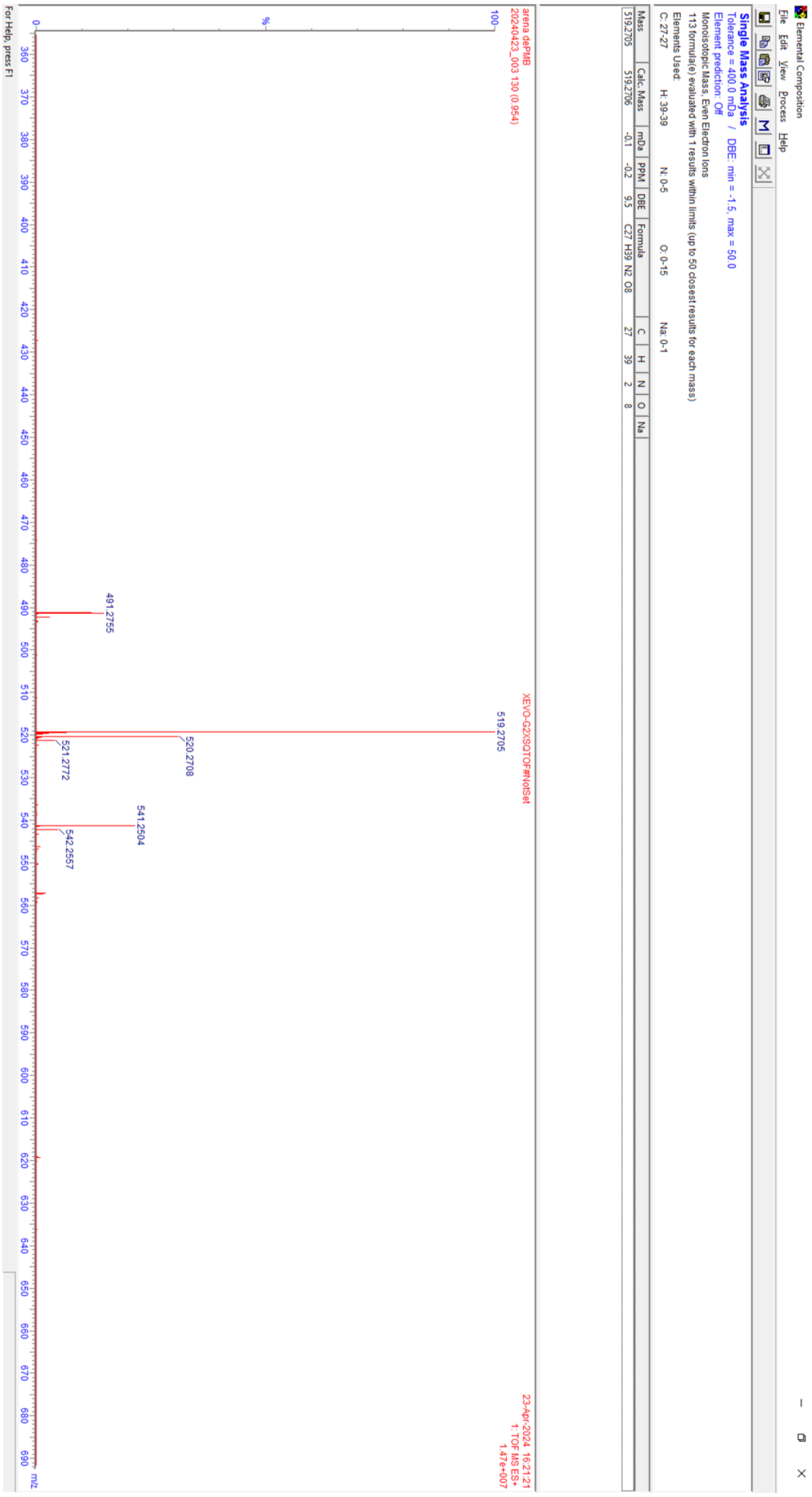

**$^{1}$H NMR** (500 MHz, $CDCl_3$)

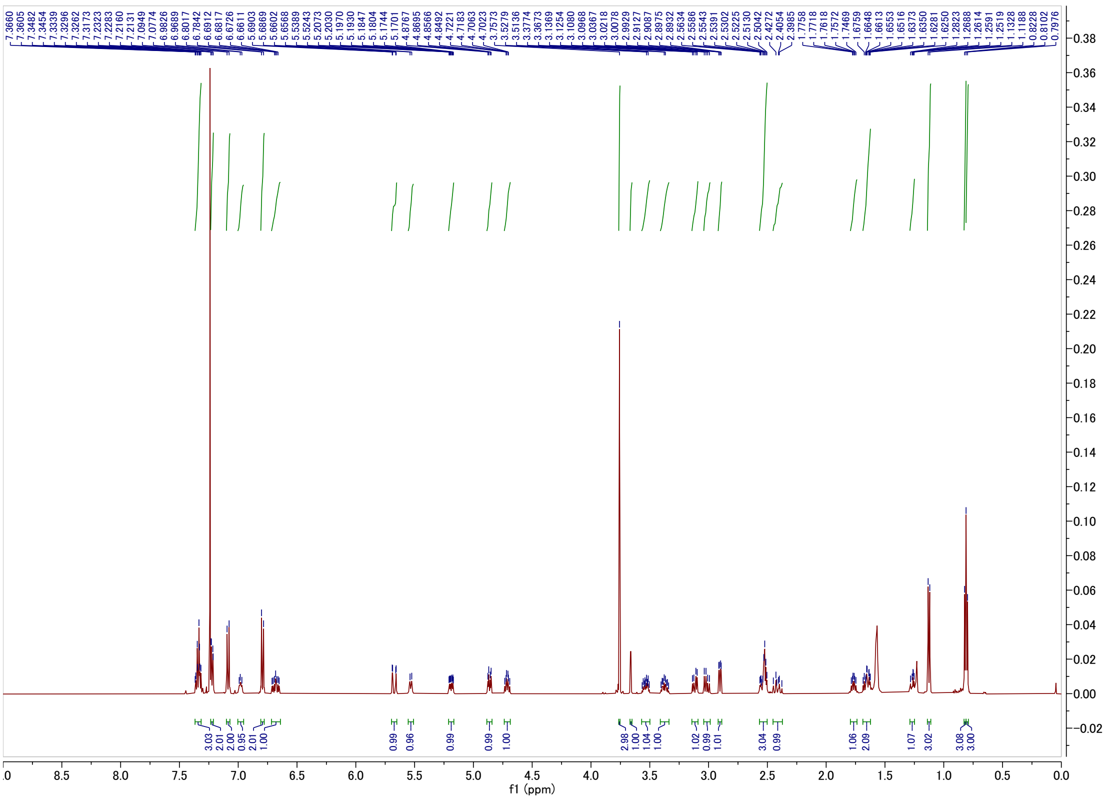


**$^{13}$C NMR** (125 MHz, $CDCl_3$)

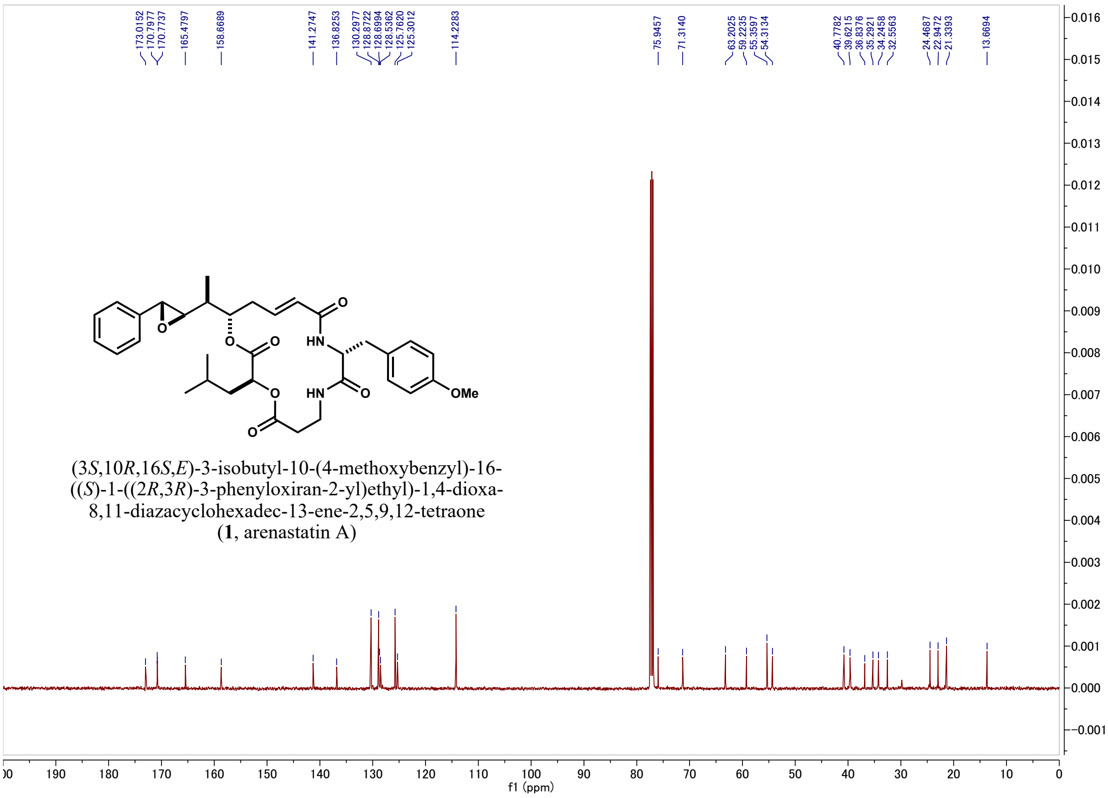


(3*S*,10*R*,16*S*,*E*)-3-isobutyl-10-(4-methoxybenzyl)-16-((*S*)-1-((2*R*,3*R*)-3-phenyloxiran-2-yl)ethyl)-1,4-dioxa-8,11-diazacyclohexadec-13-ene-2,5,9,12-tetraone
(**1**, arenastatin A)

HRMS spectrum of **1**

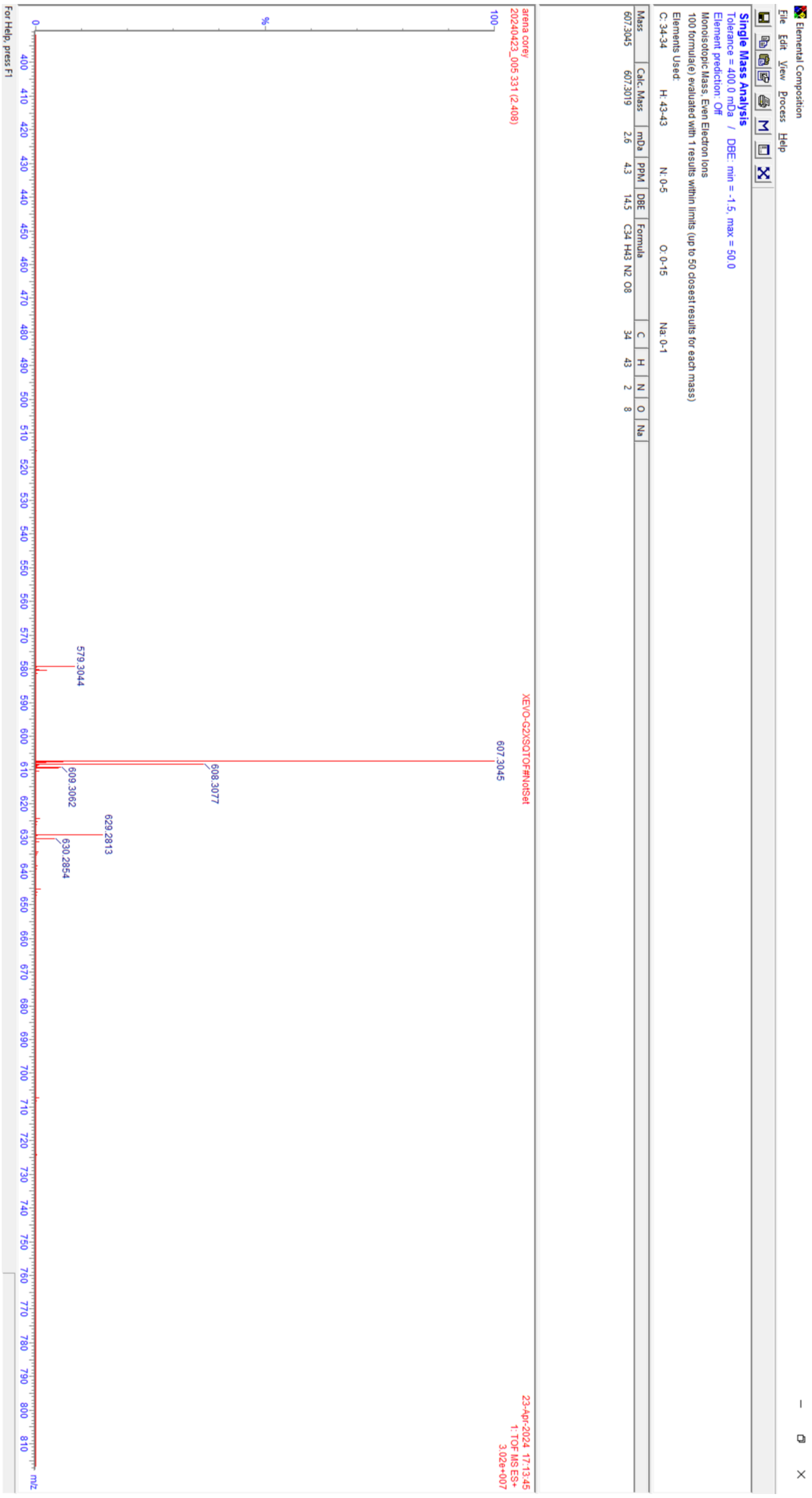

**$^1$H NMR** (500 MHz, CDCl$_3$)

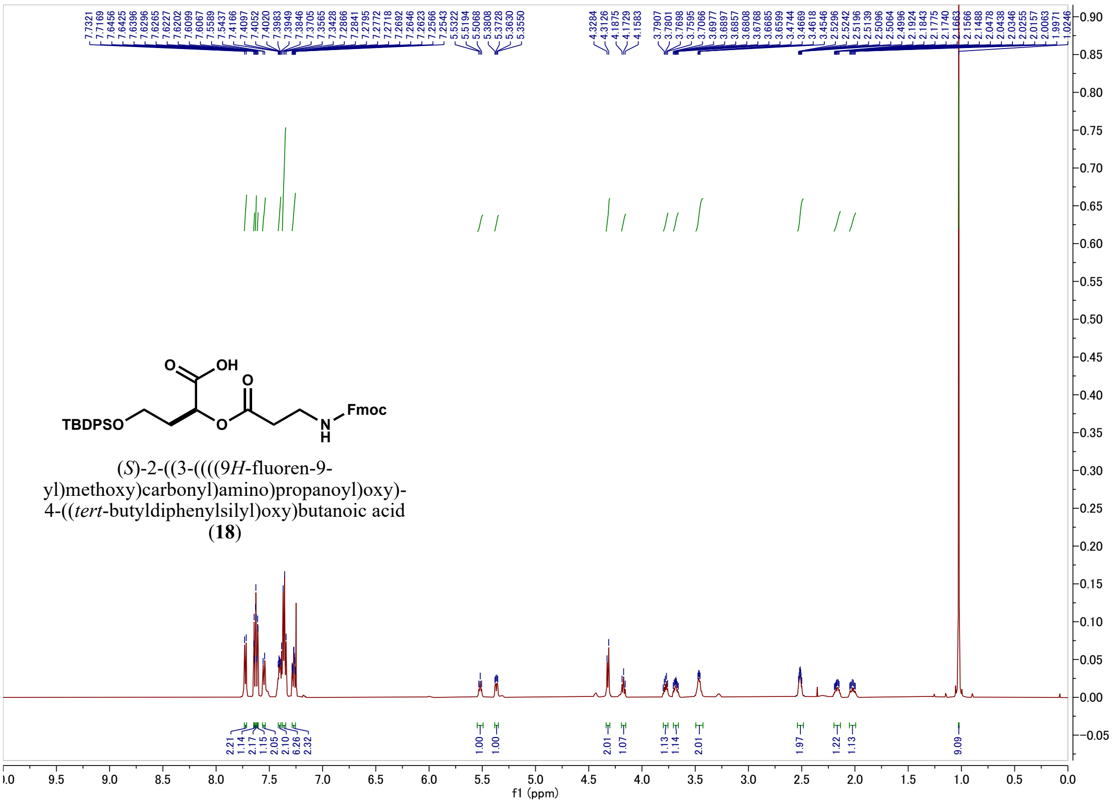

(*S*)-2-((3-((((9*H*-fluoren-9-yl)methoxy)carbonyl)amino)propanoyl)oxy)-4-((*tert*-butyldiphenylsilyl)oxy)butanoic acid (**18**)

**$^{13}$C NMR** (125 MHz, CDCl$_3$)

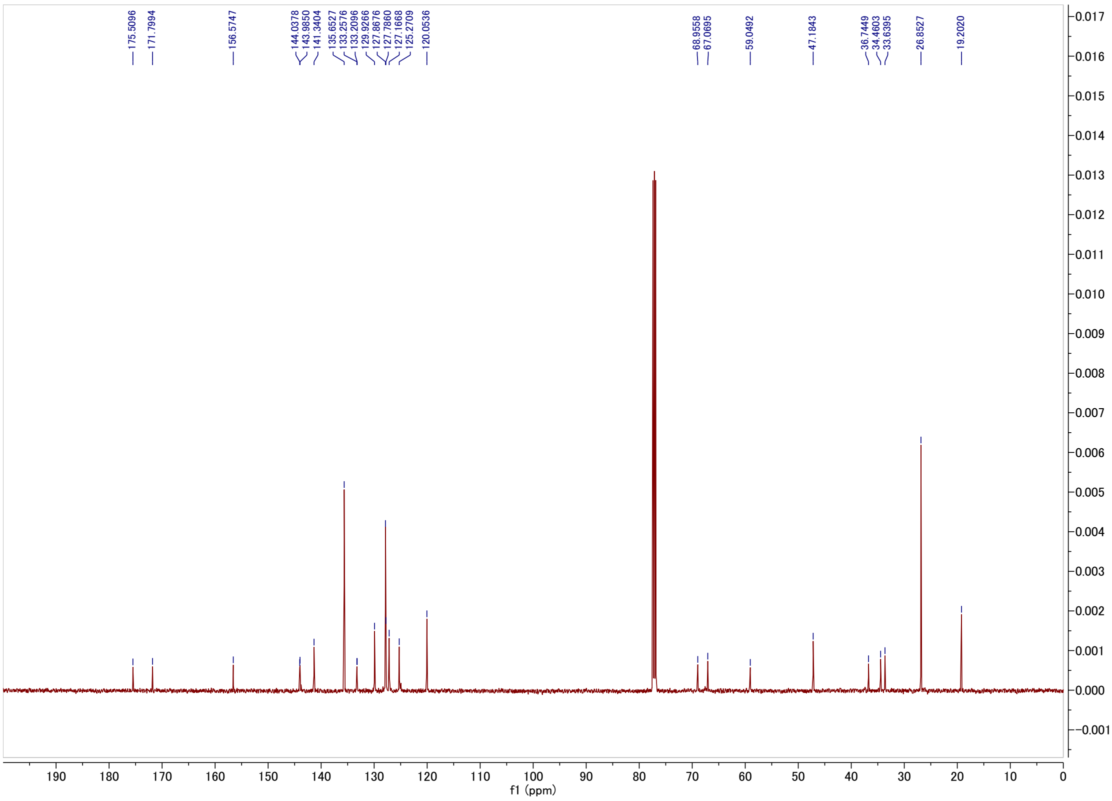

HRMS spectrum of **18**

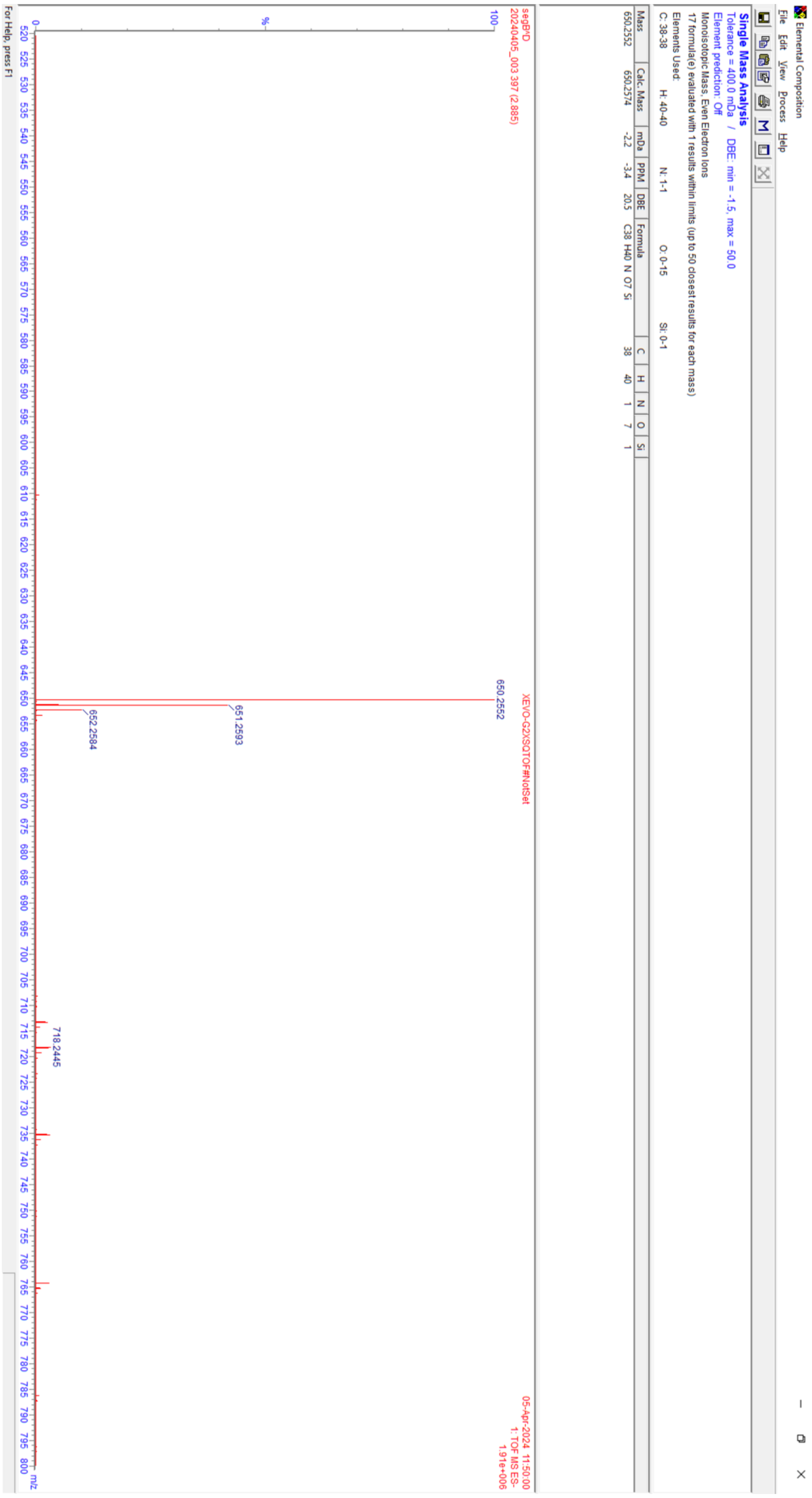

**$^{1}$H NMR** (500 MHz, $CDCl_3$)

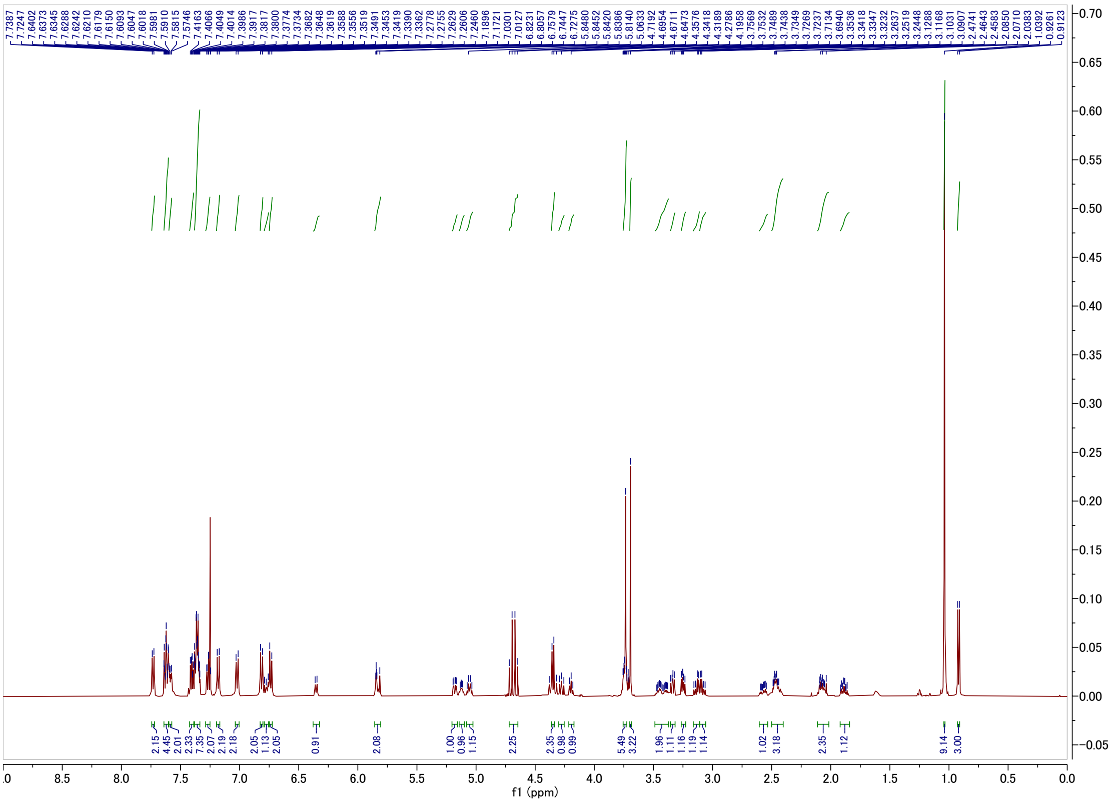


**$^{13}$C NMR** (125 MHz, $CDCl_3$)

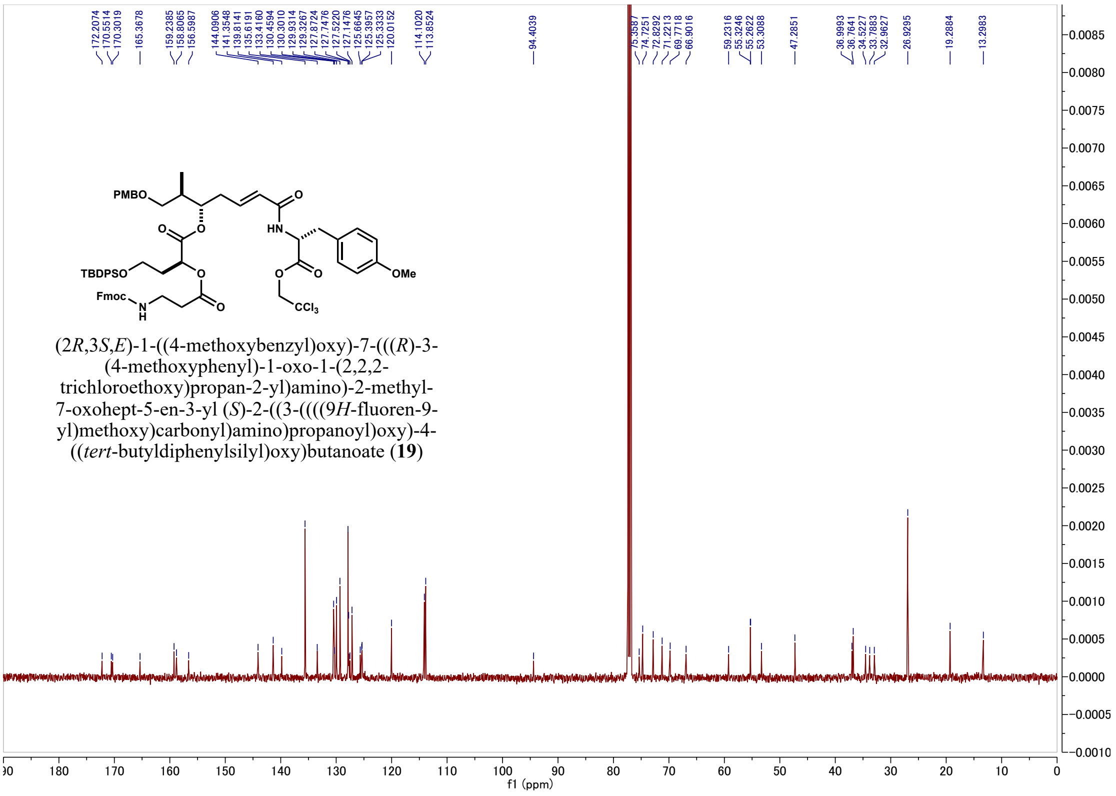


(2*R*,3*S*,*E*)-1-((4-methoxybenzyl)oxy)-7-(((*R*)-3-(4-methoxyphenyl)-1-oxo-1-(2,2,2-trichloroethoxy)propan-2-yl)amino)-2-methyl-7-oxohept-5-en-3-yl (*S*)-2-((3-((((9*H*-fluoren-9-yl)methoxy)carbonyl)amino)propanoyl)oxy)-4-((*tert*-butyldiphenylsilyl)oxy)butanoate (**19**)

HRMS spectrum of **19**

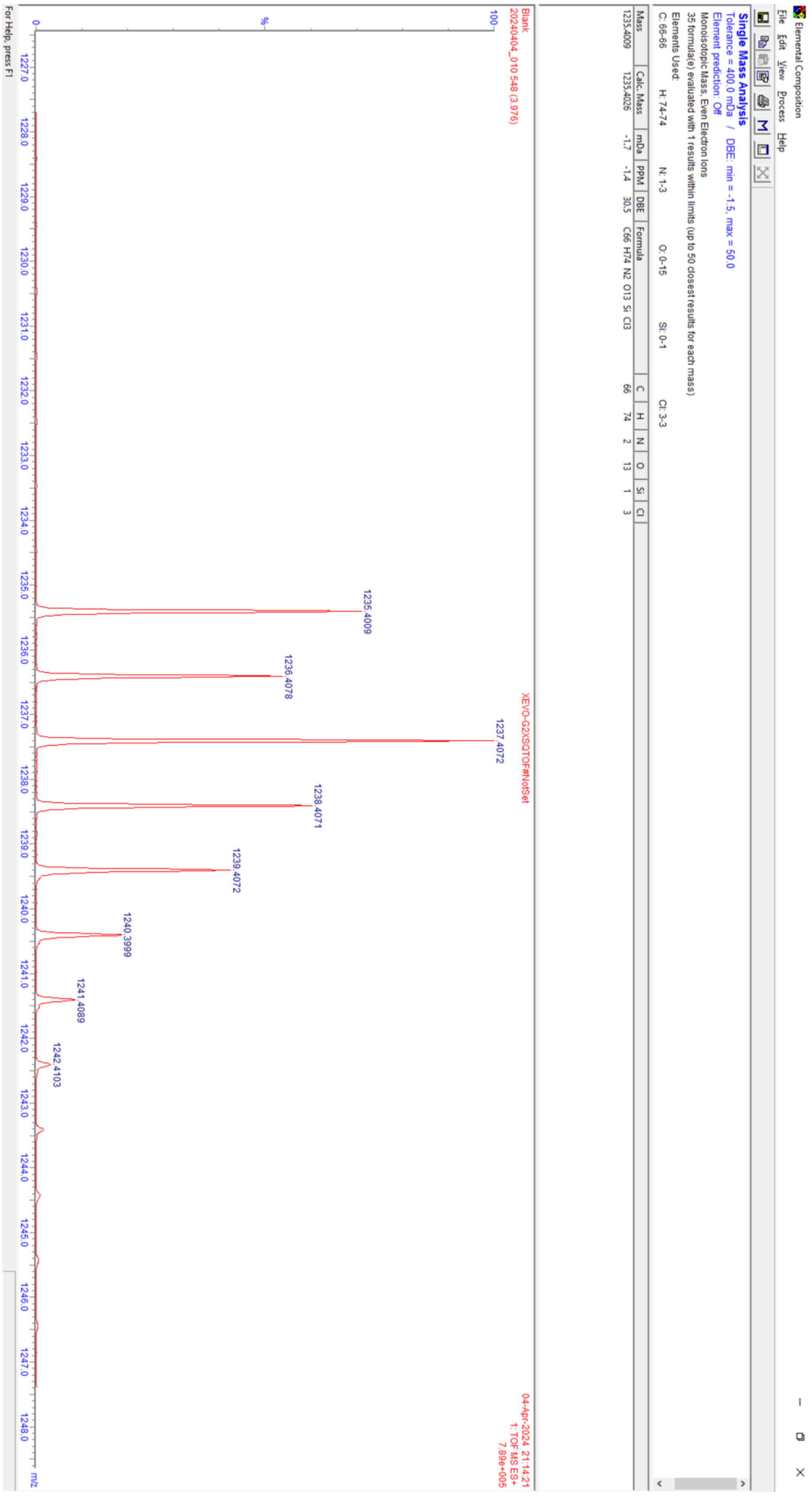

**$^{1}$H NMR** (500 MHz, $CDCl_3$)

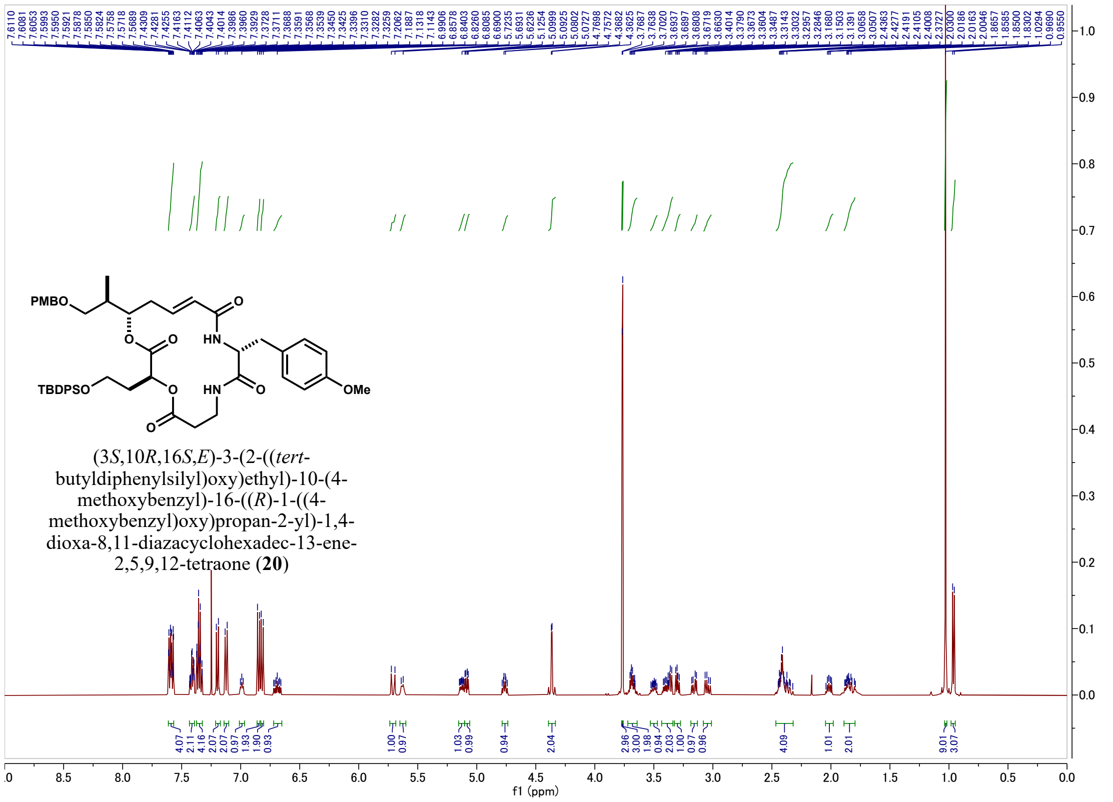

(3*S*,10*R*,16*S*,*E*)-3-(2-((*tert*-butyldiphenylsilyl)oxy)ethyl)-10-(4-methoxybenzyl)-16-((*R*)-1-((4-methoxybenzyl)oxy)propan-2-yl)-1,4-dioxa-8,11-diazacyclohexadec-13-ene-2,5,9,12-tetraone (**20**)

**$^{13}$C NMR** (125 MHz, $CDCl_3$)

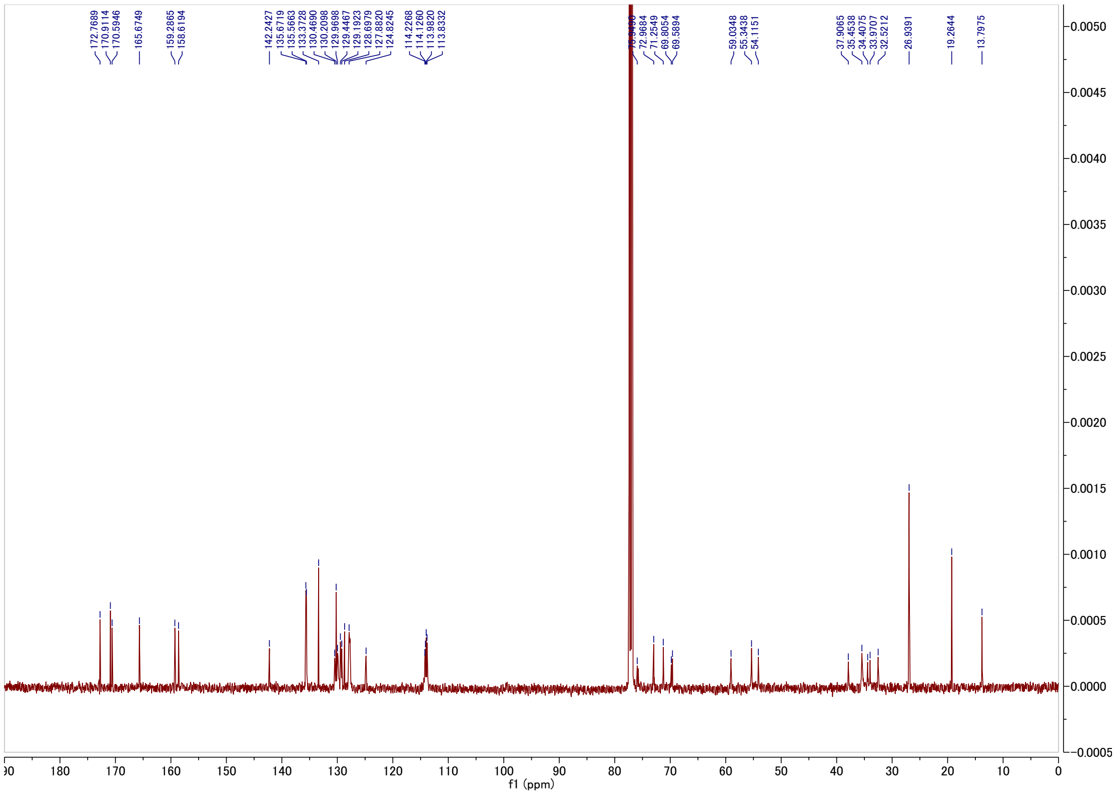

HRMS spectrum of **20**

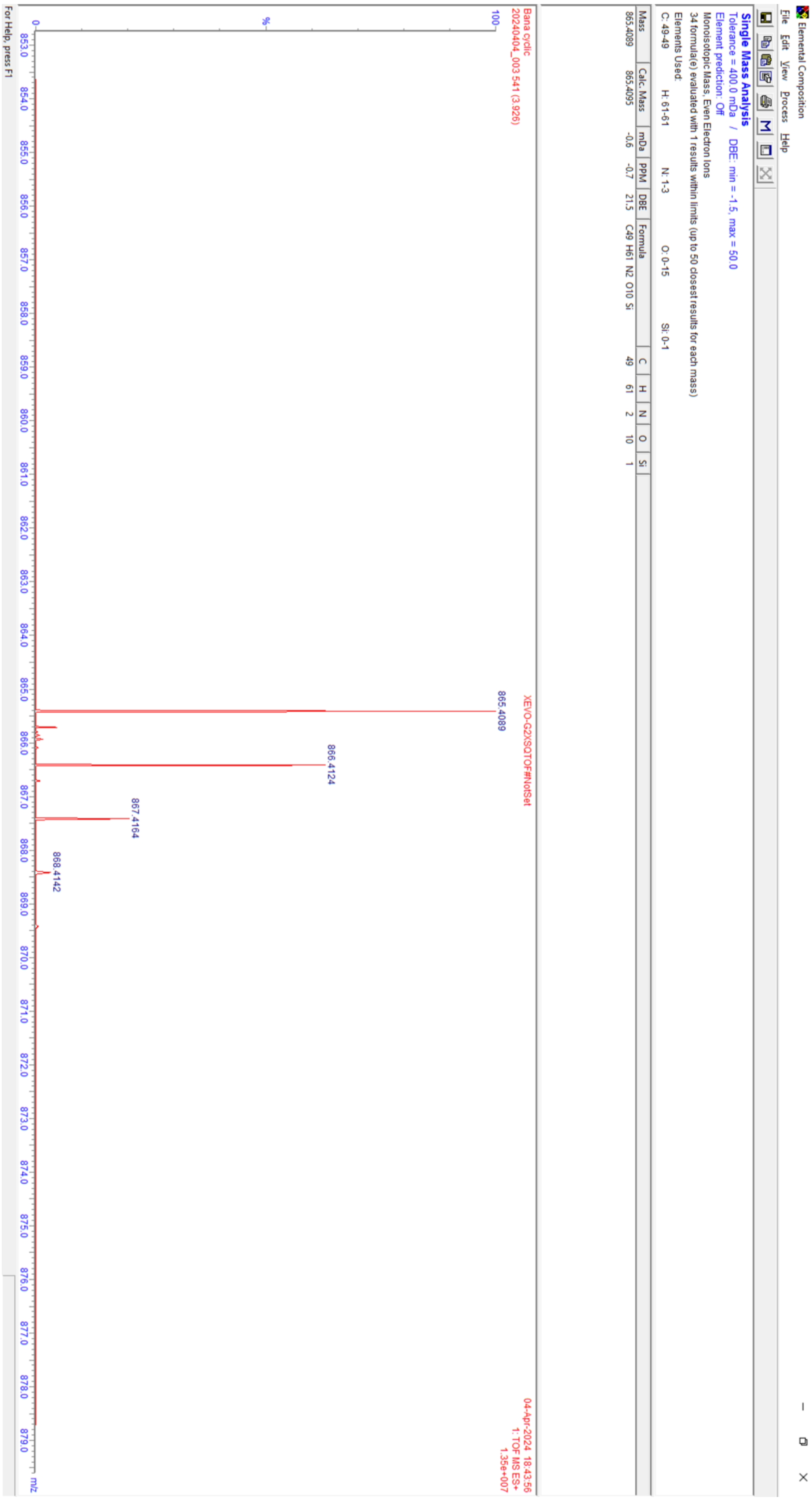

**$^{1}$H NMR** (500 MHz, $CDCl_3$)

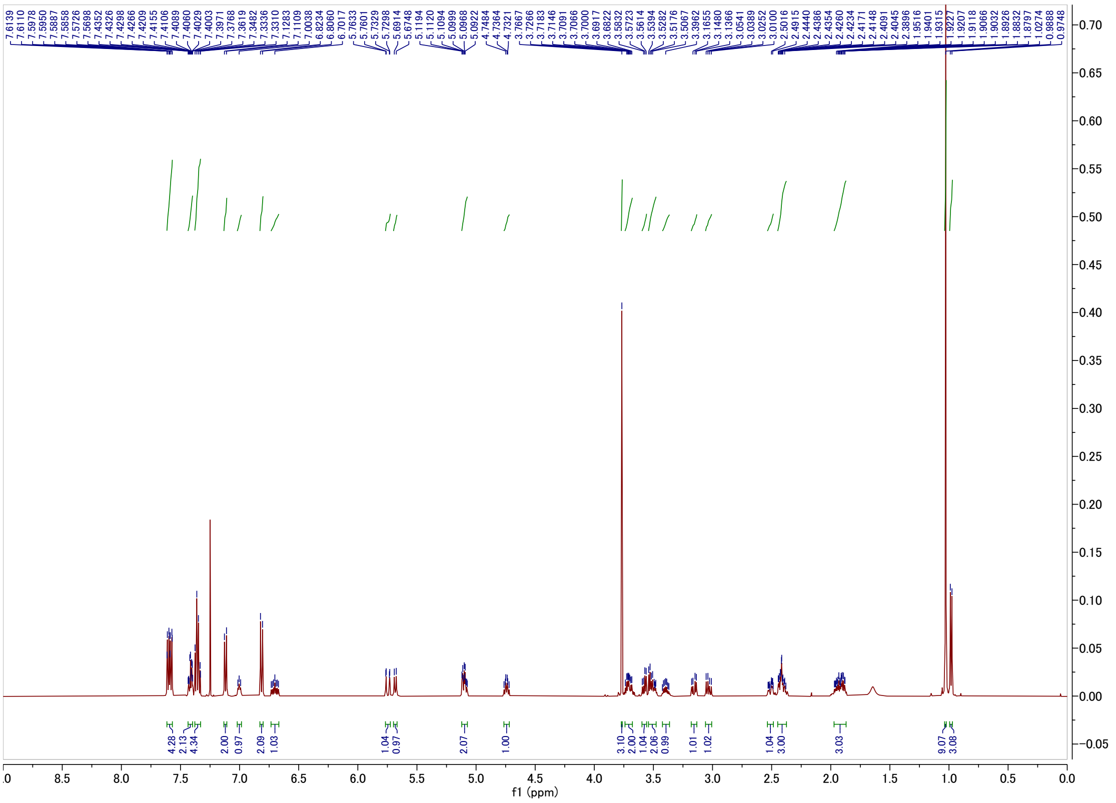


**$^{13}$C NMR** (125 MHz, $CDCl_3$)

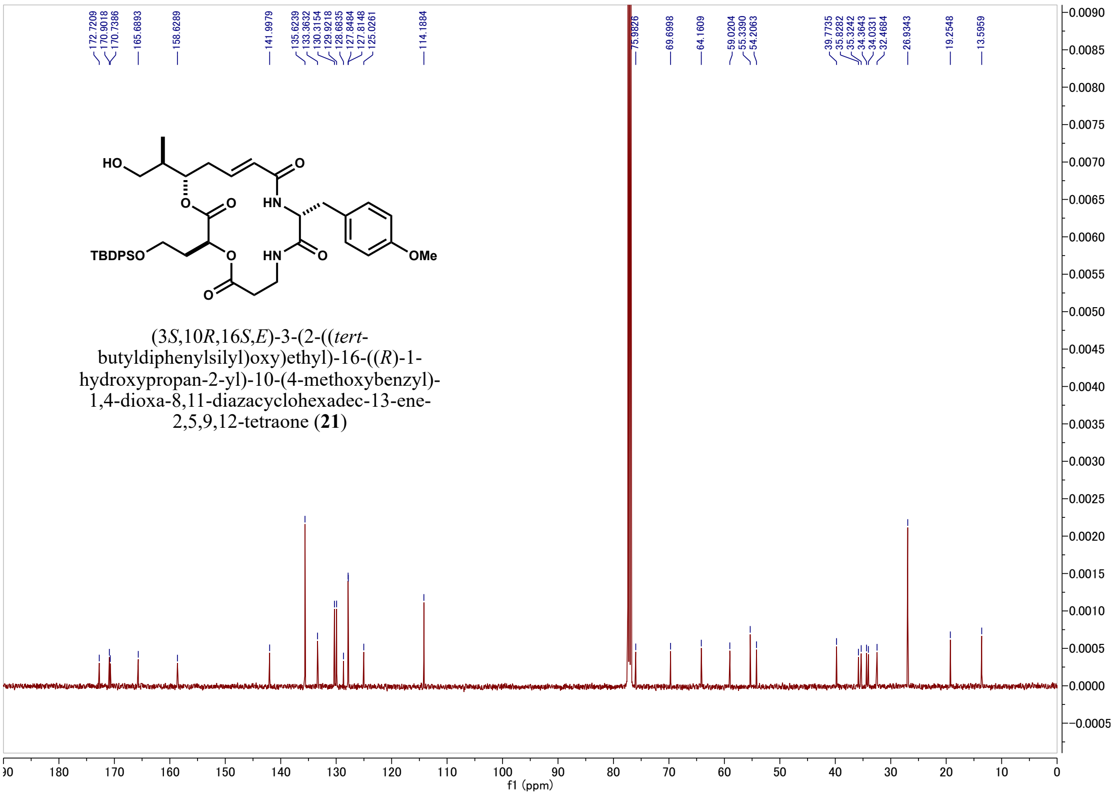


(3*S*,10*R*,16*S*,*E*)-3-(2-((*tert*-butyldiphenylsilyl)oxy)ethyl)-16-((*R*)-1-hydroxypropan-2-yl)-10-(4-methoxybenzyl)-1,4-dioxa-8,11-diazacyclohexadec-13-ene-2,5,9,12-tetraone (**21**)

HRMS spectrum of **21**

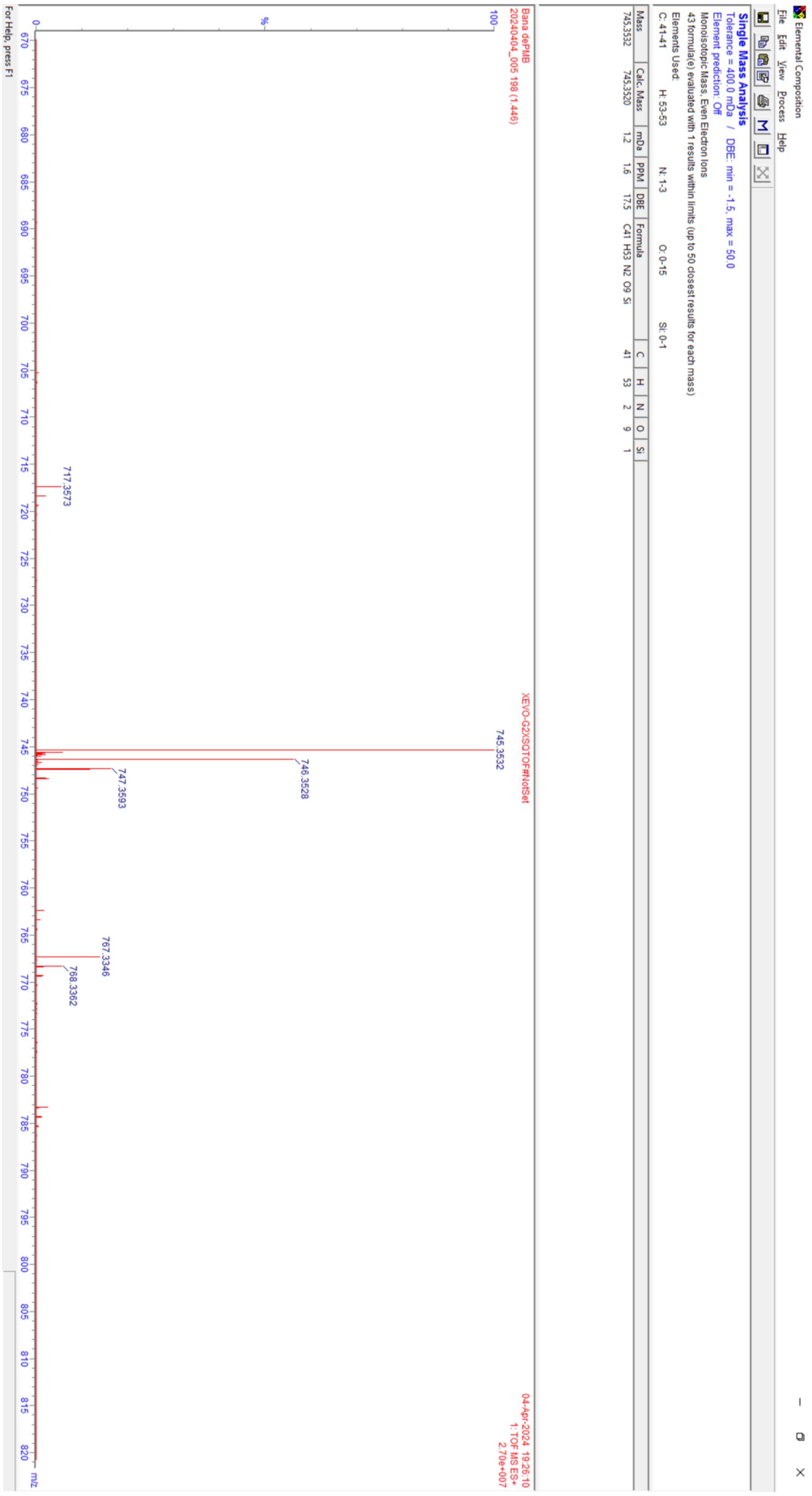

## $^{1}$H NMR (500 MHz, CDCl$_3$)

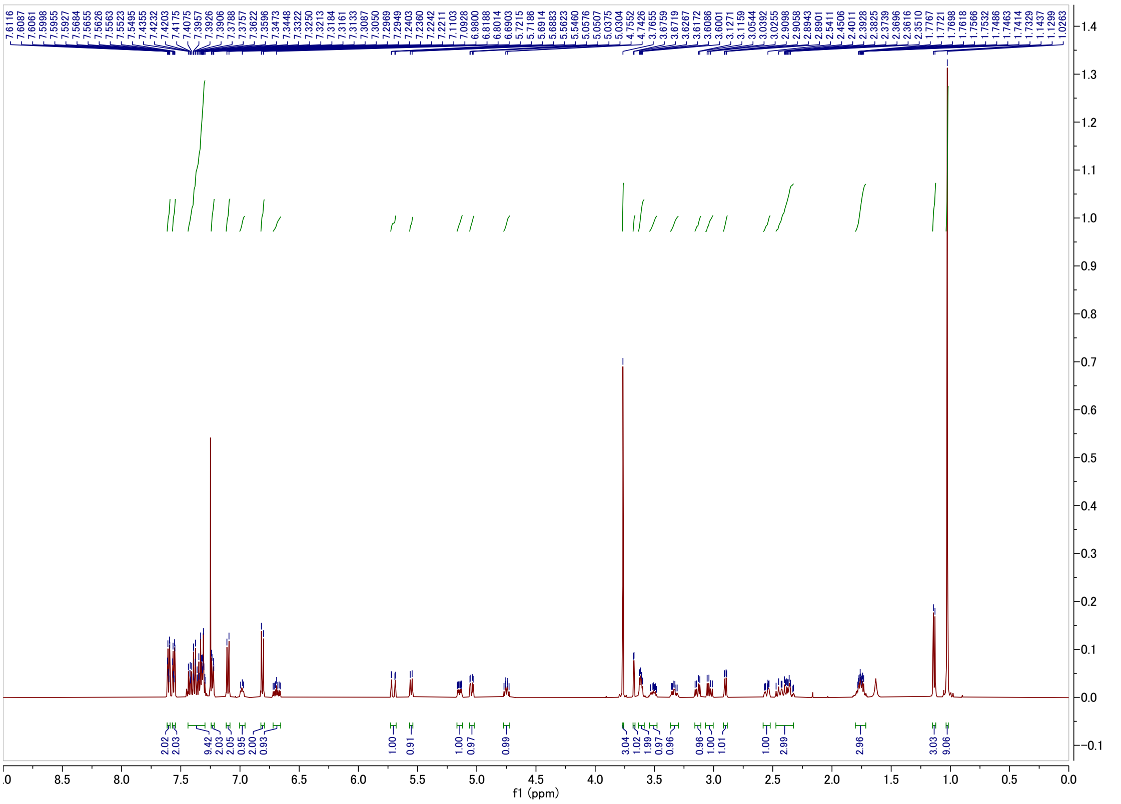


## $^{13}$C NMR (125 MHz, CDCl$_3$)

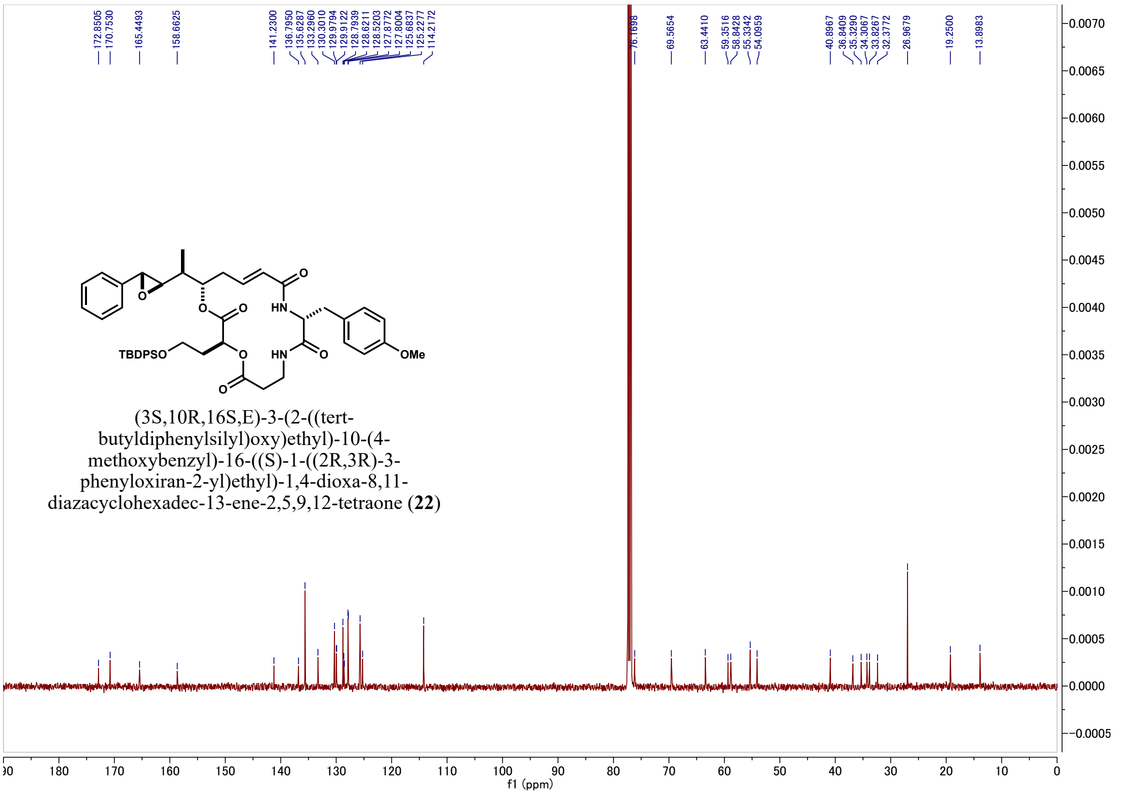


(3S,10R,16S,E)-3-(2-((tert-butyldiphenylsilyl)oxy)ethyl)-10-(4-methoxybenzyl)-16-((S)-1-((2R,3R)-3-phenyloxiran-2-yl)ethyl)-1,4-dioxa-8,11-diazacyclohexadec-13-ene-2,5,9,12-tetraone (**22**)

HRMS spectrum of **22**

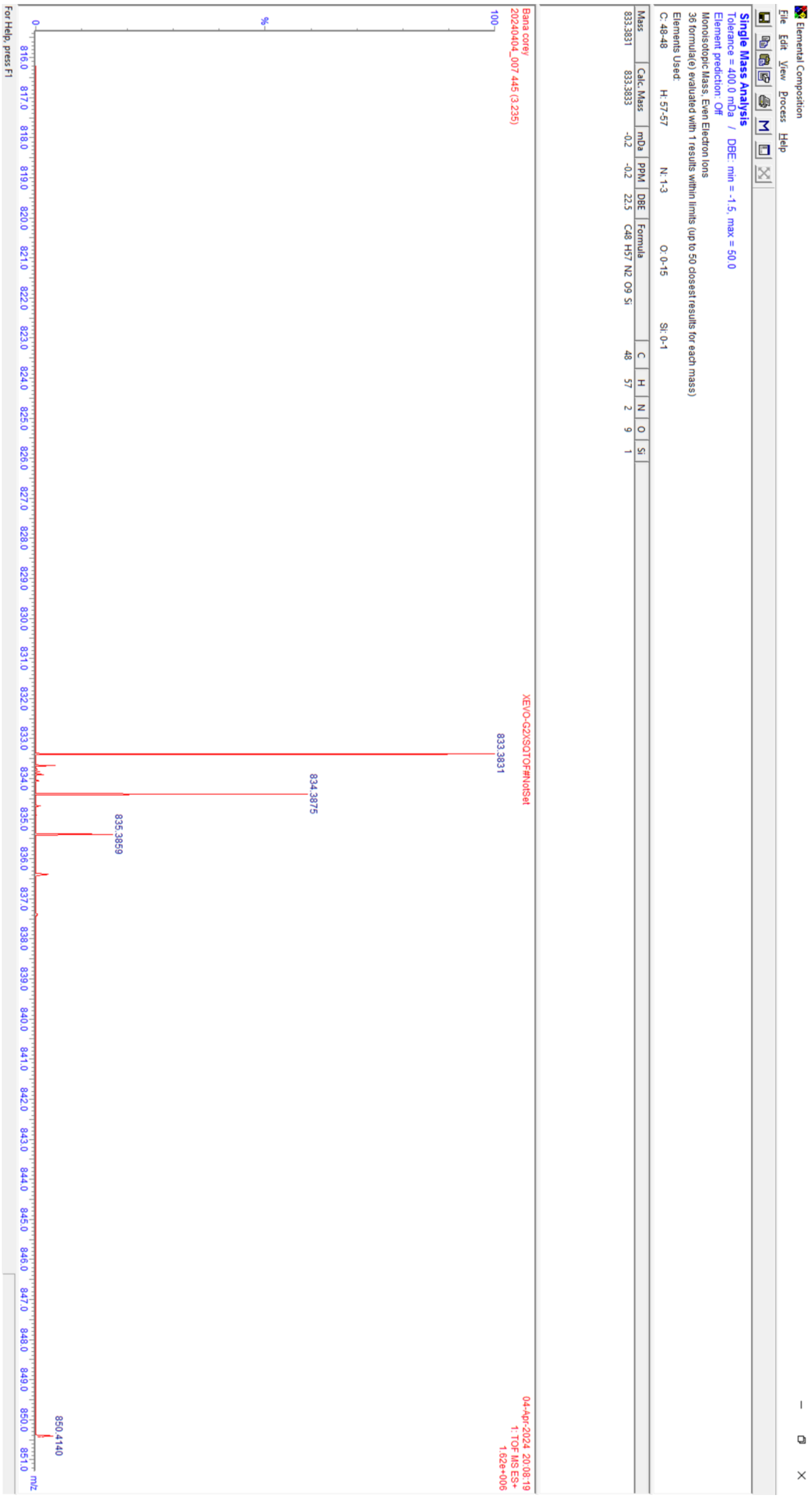

**$^{1}$H NMR** (500 MHz, $CDCl_3$)

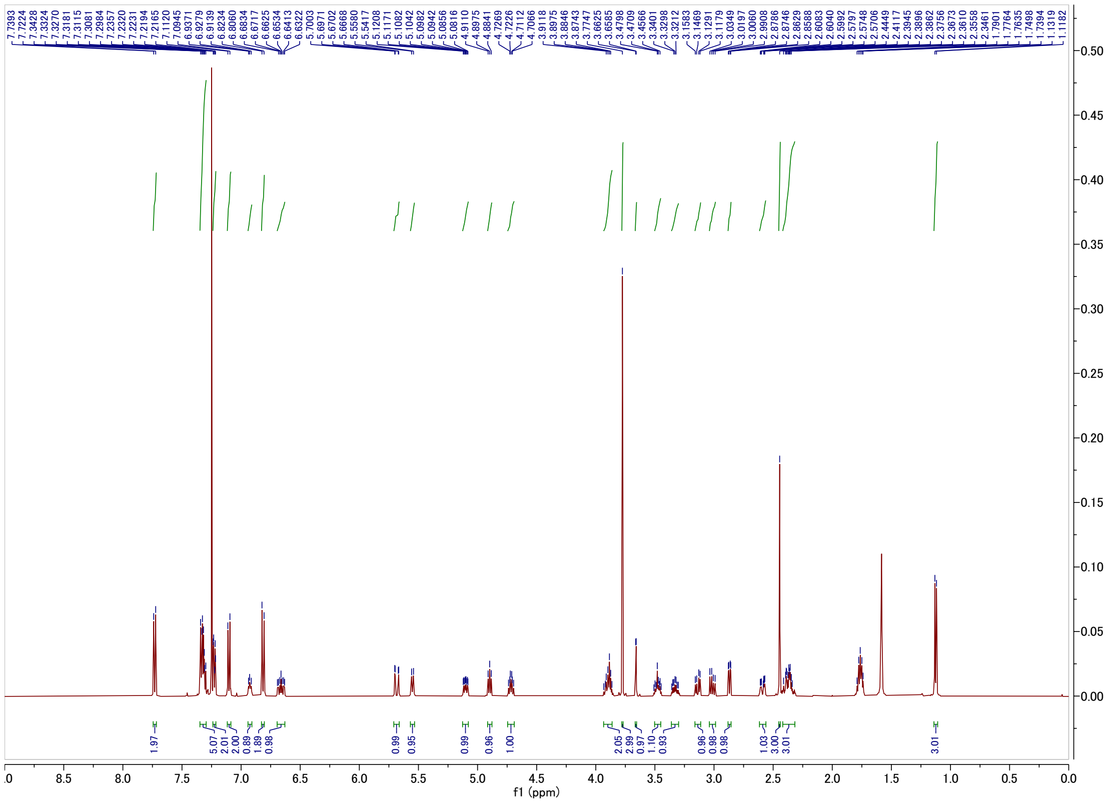

**$^{13}$C NMR** (125 MHz, $CDCl_3$)

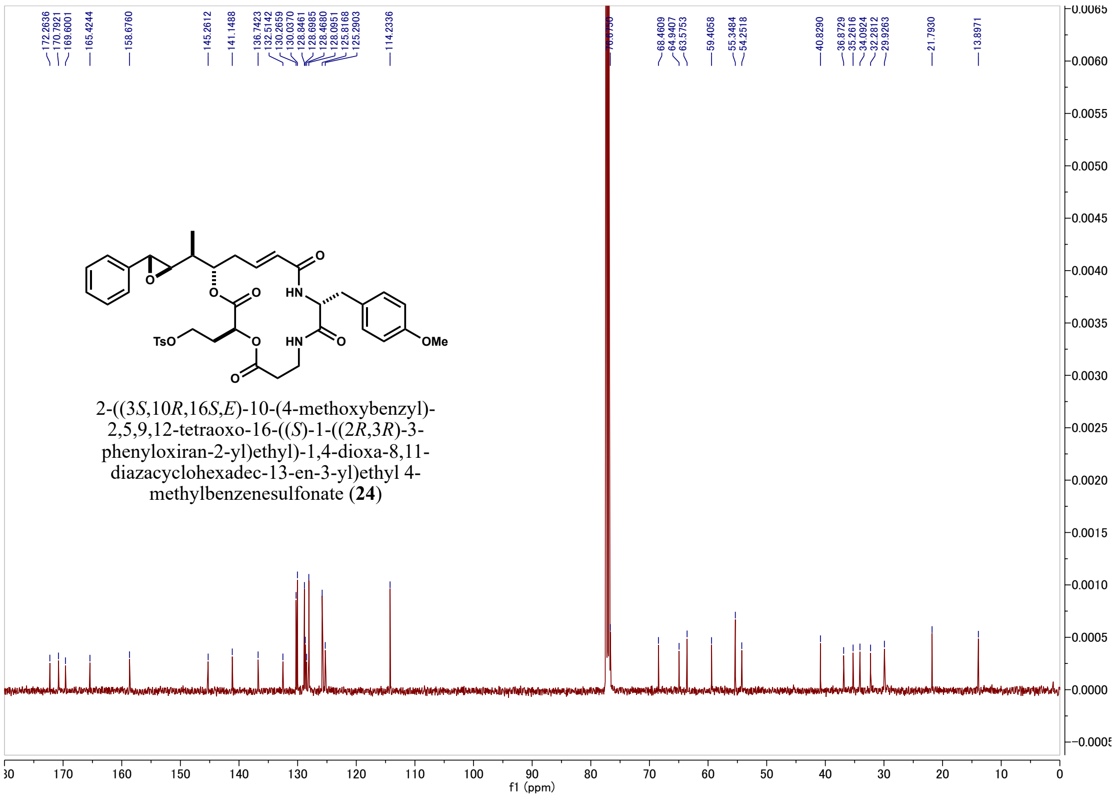

HRMS spectrum of **24**

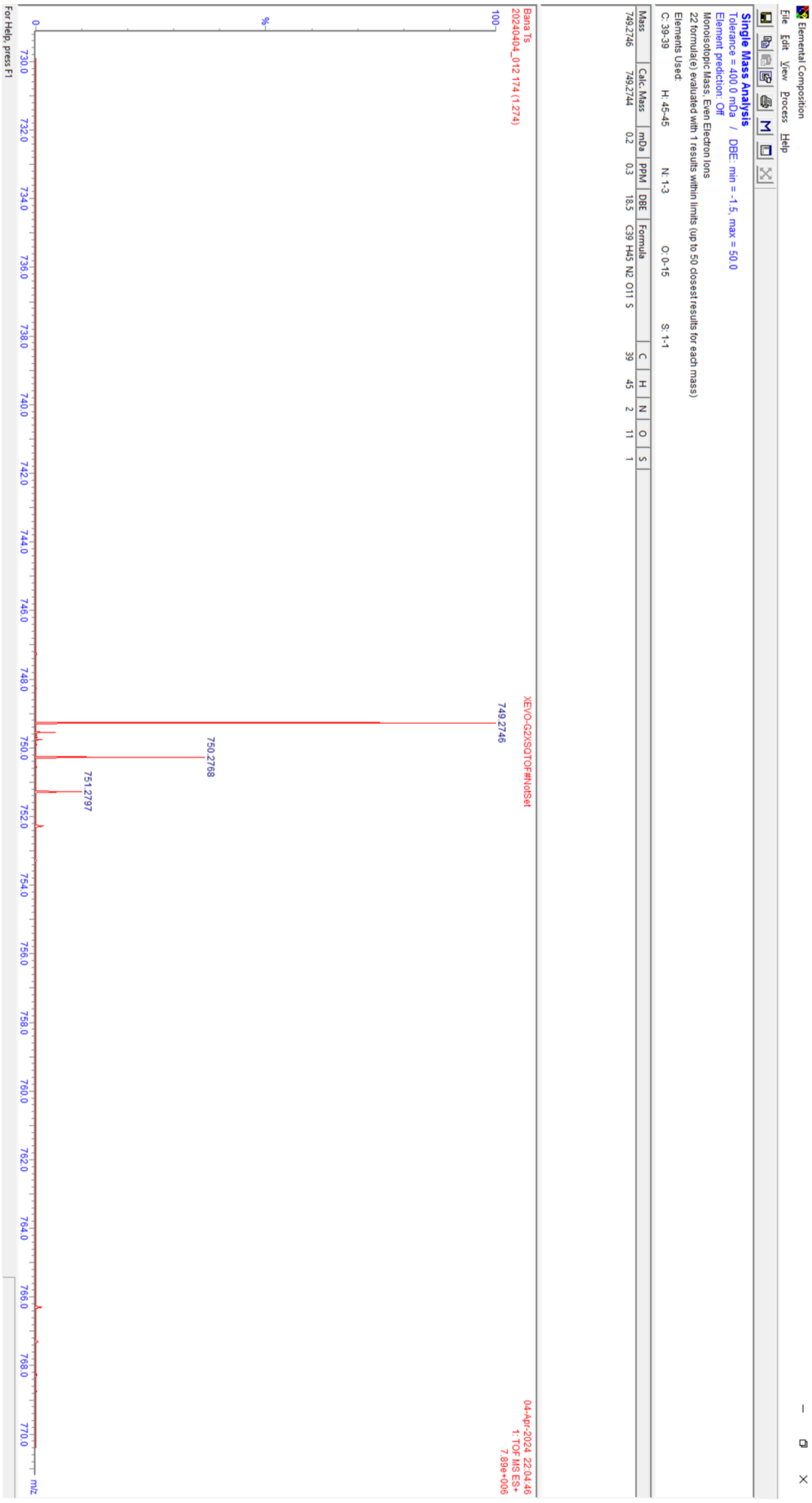

**$^{1}$H NMR** (500 MHz, $CDCl_3$)

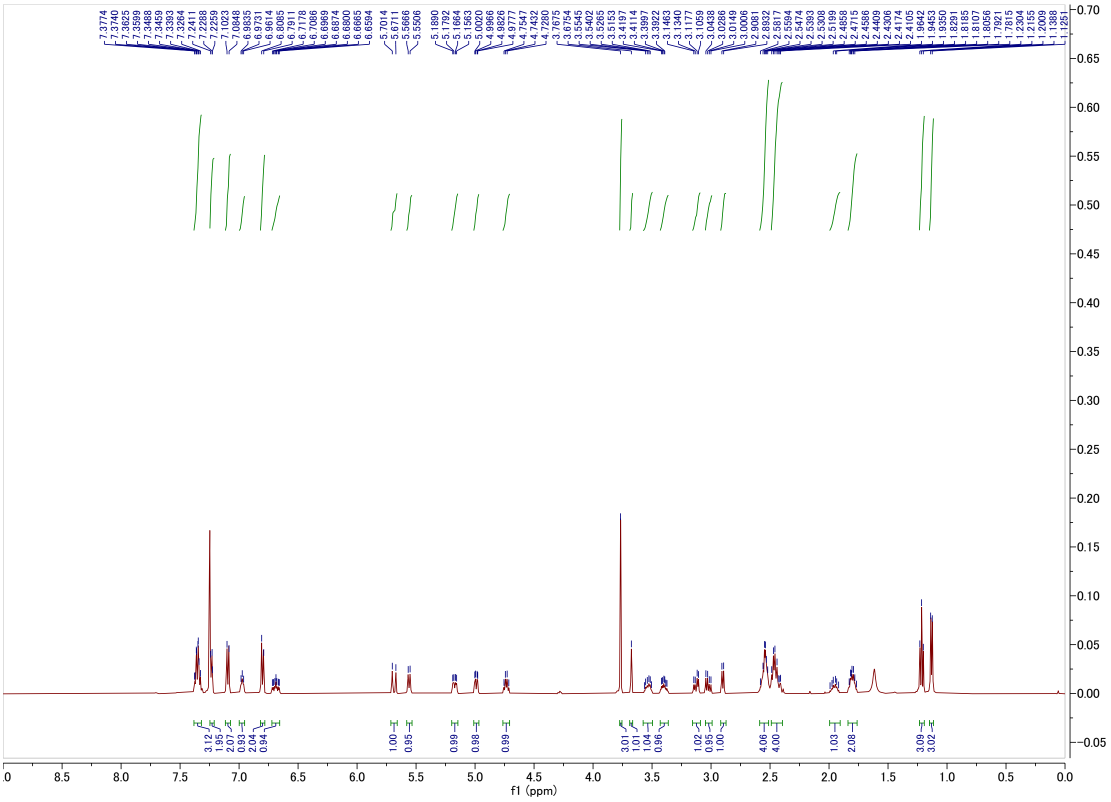


**$^{13}$C NMR** (125 MHz, $CDCl_3$)

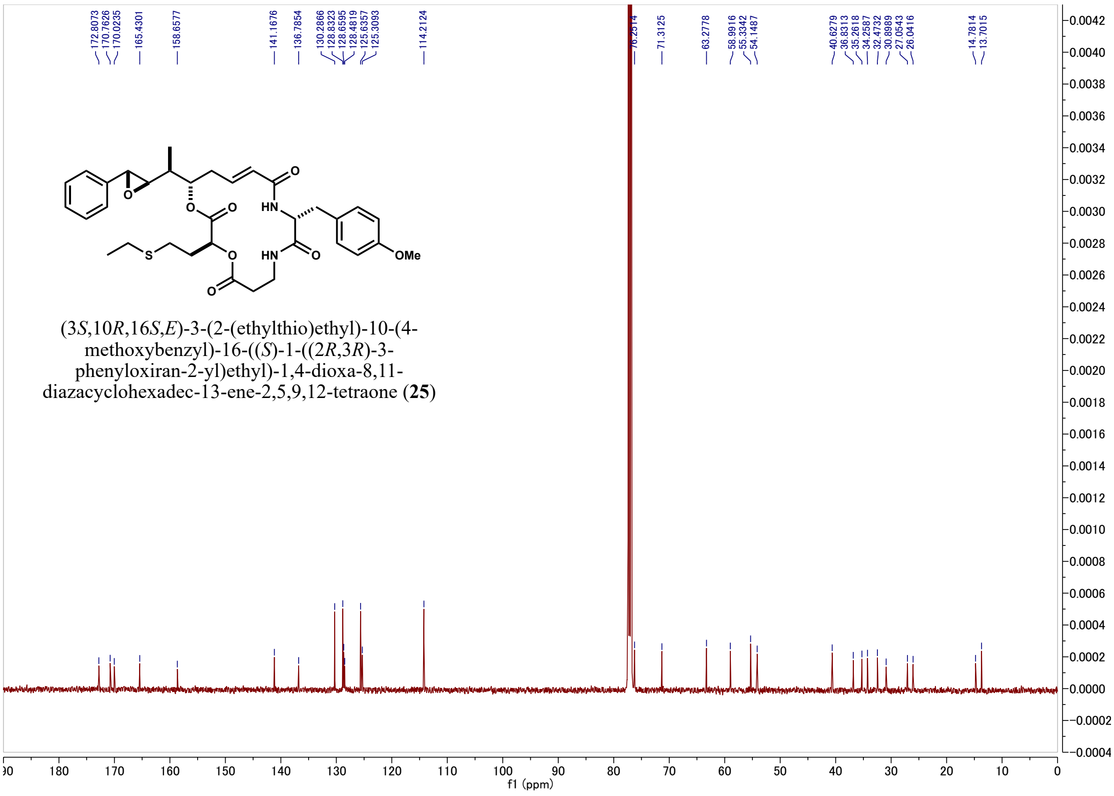


(3*S*,10*R*,16*S*,*E*)-3-(2-(ethylthio)ethyl)-10-(4-methoxybenzyl)-16-((*S*)-1-((2*R*,3*R*)-3-phenyloxiran-2-yl)ethyl)-1,4-dioxa-8,11-diazacyclohexadec-13-ene-2,5,9,12-tetraone (**25**)

HRMS spectrum of **25**

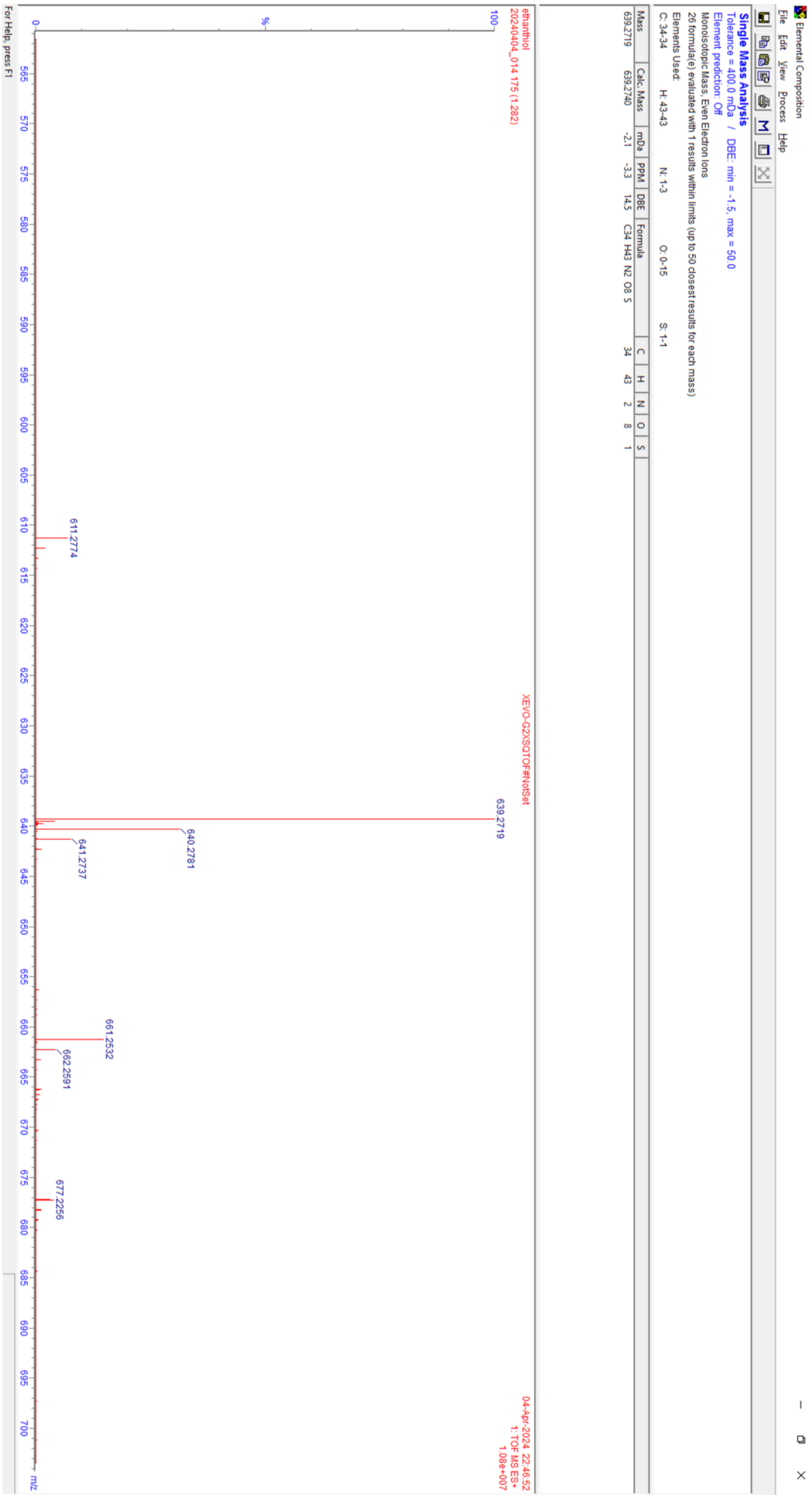

**$^{1}$H NMR** (500 MHz, $CDCl_3$)

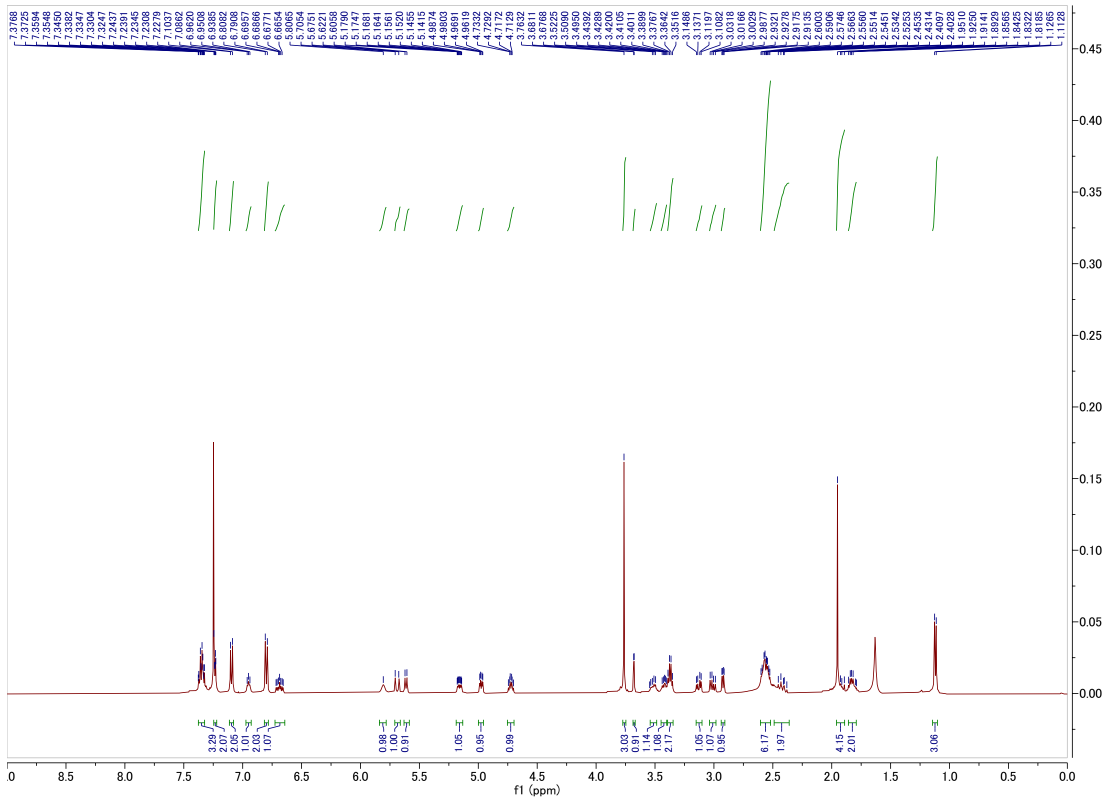

**$^{13}$C NMR** (125 MHz, $CDCl_3$)

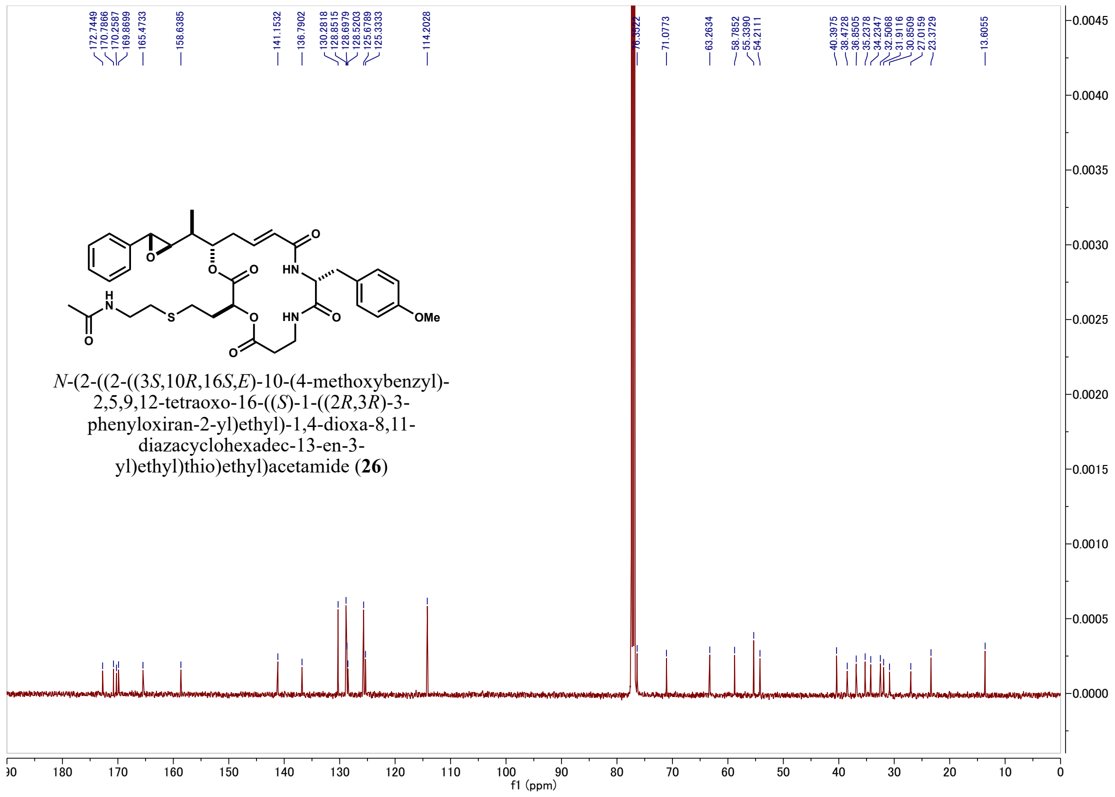

*N*-(2-((2-((3*S*,10*R*,16*S*,*E*)-10-(4-methoxybenzyl)-2,5,9,12-tetraoxo-16-((*S*)-1-((2*R*,3*R*)-3-phenyloxiran-2-yl)ethyl)-1,4-dioxa-8,11-diazacyclohexadec-13-en-3-yl)ethyl)thio)ethyl)acetamide (**26**)

HRMS spectrum of **26**

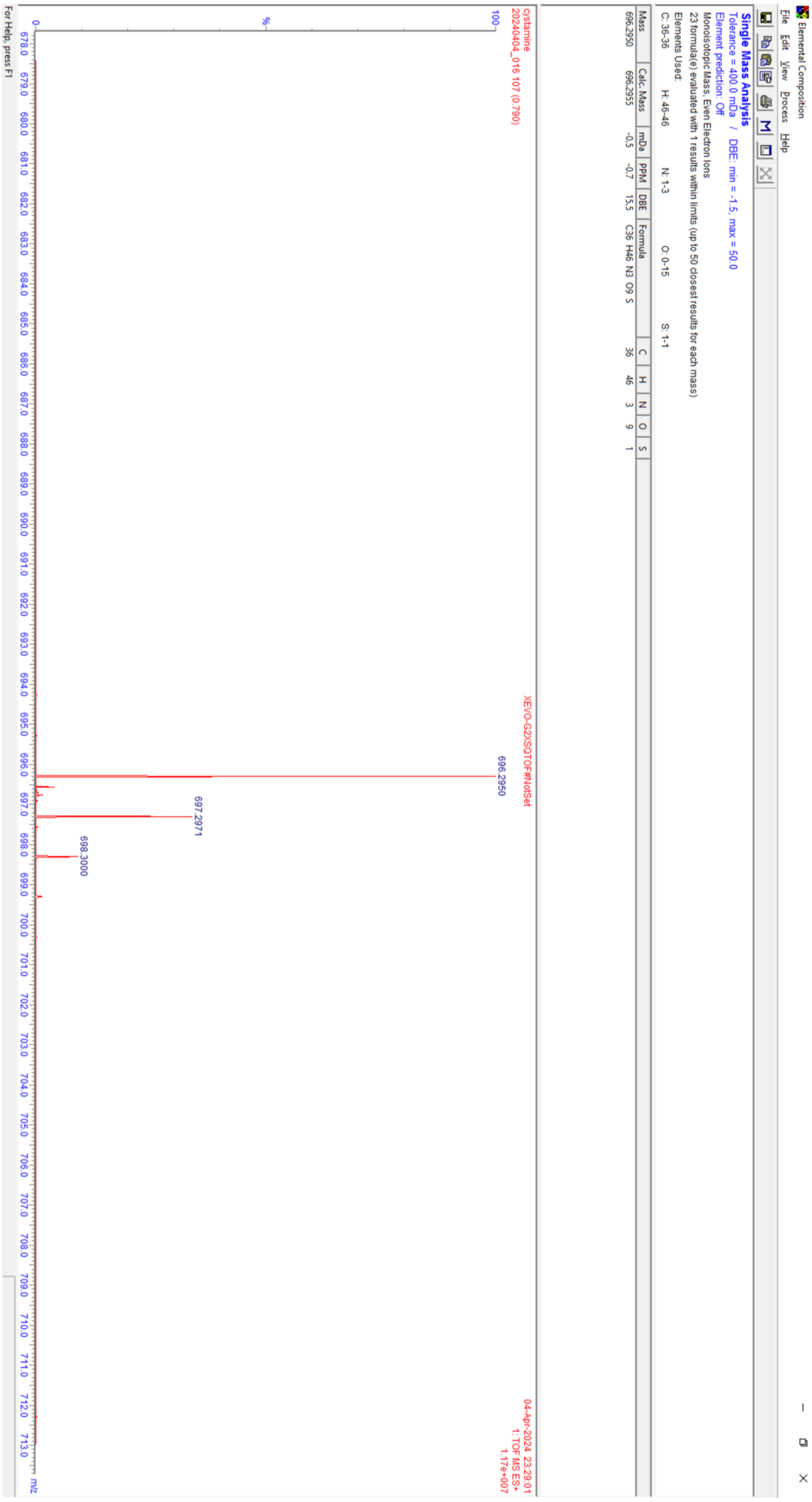

Supplement: Supplementary file 1 [file molecules-29-04058-s001.zip › molecules-3160584-supplementary.pdf]
